# Towards a DNA barcode library for Madagascar’s threatened ichthyofauna

**DOI:** 10.1371/journal.pone.0271400

**Published:** 2022-08-11

**Authors:** Miguel Vences, Dominik Stützer, Noromalala Rasoamampionona Raminosoa, Thomas Ziegler

**Affiliations:** 1 Zoological Institute, Braunschweig, Germany; 2 Mention Zoologie et Biodiversité Animale, Université d’Antananarivo, Antananarivo, Madagascar; 3 AG Zoologischer Garten Köln, Köln, Germany; Nanjing Agricultural University, CHINA

## Abstract

In order to improve the molecular resources available for conservation management of Madagascar’s threatened ichthyofauna, we elaborated a curated database of 2860 mitochondrial sequences of the mitochondrial *COI*, *16S* and *ND2* genes of Malagasy fishes, of which 1141 sequences of freshwater fishes were newly sequenced for this data set. The data set is mostly composed of *COI* (2015 sequences) while *16S* and *ND2* sequences from partly the same samples were used to match the *COI* sequences to reliably identified reference sequences of these genes. We observed *COI* uncorrected pairwise genetic distances of 5.2‒31.0% (mean 20.6%) among species belonging to different genera, and 0.0‒22.4% (mean 6.4%) for species belonging to the same genus. Deeply divergent mitochondrial lineages of uncertain attribution were found among Malagasy freshwater eleotrids and gobiids, confirming these groups are in need of taxonomic revision. DNA barcodes assigned to introduced cichlids (tilapias) included *Coptodon rendallii*, *C*. *zillii*, *Oreochromis aureus* (apparently a new country record), *O*. cf. *mossambicus*, *O*. *niloticus*, and one undetermined species of *Oreochromis*, with sequences of up to three species found per location. In aplocheiloid killifishes of the genus *Pachypanchax*, most species from northern Madagascar had only low mitochondrial divergences, three of these species (*P*. *omalonotus*, *P*. *patriciae*, and *P*. *varatraza*) were not reciprocally monophyletic, and one genetically deviant lineage was discovered in a northern locality, suggesting a need for partial taxonomic revision of this genus. While the lack of voucher specimens for most of the samples sequenced herein precludes final conclusions, our first step towards a DNA barcoding reference library of Madagascar’s fishes already demonstrates the value of such a data set for improved taxonomic inventory and conservation management. We strongly suggest further exploration of Madagascar’s aquatic environments, which should include detailed photographic documentation and tissue sampling of large numbers of specimens, and collection of preserved voucher specimens as well as of living fish for the buildup of ex situ assurance populations of threatened species complying with the One Plan Approach proposed by the IUCN SSC Conservation Breeding Specialist Group (CBSG).

## Introduction

Madagascar, one of the globe’s most important hotspots for biodiversity conservation [1], is characterized by a unique freshwater biota [2]. It harbors a rich marine ichthyofauna [3] but given the island’s isolation from other landmasses for over 65 million years, and the limited overseas dispersal capacity of many groups of freshwater organisms, only few clades of fishes have been able to colonize its freshwater habitats [4]. Based on cladistic considerations, Gondwanan origins of Malagasy freshwater fishes have been hypothesized [5, 6] but molecular clock analyses and Bayesian analysis of fossil occurrences, suggest that the Malagasy clades are younger and may have originated by overseas dispersal [7–11].

Despite the presence of only three clades of primary freshwater fishes (cichlids, family Cichlidae; Madagascar rainbowfishes, Bedotiidae; Old World killifishes, Aplocheilidae), Madagascar’s inland waters harbor a species richness comparable to other landmasses of similar size [12]. Early researchers interpreted this freshwater ichthyofaunal assemblage as depauperate [13] but Sparks and Stiassny [12] already listed as many as 143 native Malagasy freshwater fish species, and 26 new species have been scientifically named since [14, 15]. The latest comprehensive checklist [3] lists 90 native freshwater species (defined as species where adults live mainly in this environment), as well as 28 introduced species (all from freshwater or transitional habitats), thus totaling 118 fish species occurring in Madagascar’s freshwaters. The lower number of species in the list of Fricke et al. [3] compared to that of Sparks and Stiassny [12], despite the discovery of numerous additional species since 2003, probably reflects (i) the comparatively poor taxonomic knowledge of a substantial part of this fauna, (ii) misidentifications especially in historical work, as well as (iii) difficulties in assigning euryhaline fishes to the freshwater vs. marine categories. The entire known (freshwater + marine) ichthyofauna of Madagascar comprises a total of 1,798 species in 247 families [3]. Due to the combined effects of deforestation, overfishing, and introduction of exotic species, Madagascar’s freshwater biota, including fishes, is highly threatened [16]. In fact freshwater fishes are often considered to be the most threatened group of vertebrates on Madagascar. Riverine fishes are severely affected by a dramatic loss of forest habitats which causes sedimentation of spawning beds and alters water flow, quality and nutrient input [17]. Secondary vegetation growing on hills cleared by slash-and-burn agriculture is typically insufficient to anchor soils [18], leading to continuous erosion and thus sedimentation of aquatic habitats. In Madagascar’s highlands, approximately 60% of wetlands and 37% of riparian forests were lost in the second half of the 20th century [19]. Furthermore, exotic fishes have displaced native species at many sites, and most fish assemblages in Madagascar are now dominated by exotics [12].

Of the 173 fish species listed from Malagasy freshwater habitats by Leiss et al. [15], 123 are exclusive freshwater inhabitants, 79 of these endemic to Madagascar, and 50 classified in one of the threatened categories of the International Union for Conservation of Nature and Natural Resources [20]; of the threatened species, 6 are categorized as Vulnerable, 30 as Endangered, and 14 as Critically Endangered [20] (summarized in [15]). Of the marine species, 37 are considered to be endemic to Madagascar, and an additional 23 subendemic, that is, their distribution is restricted to Madagascar and adjacent archipelagos or submerged ridges [3]. Conservation of Madagascar’s marine ecosystems has recently received increased attention as they are facing unprecedented threats from climate change, habitat destruction and overfishing [21, 22].

In Madagascar, comprehensive DNA barcoding data sets have been published, among other organismal groups, for amphibians, reptiles, ants, and moths [23–28]. Globally, fishes are an important group in DNA barcoding efforts [29] due to their economic importance as human food resource and the potential of this molecular approach to detect consumer fraud, aid fisheries management, and inform ecological and taxonomic research [30, 31].

DNA barcoding has been applied to Madagascar’s ichthyofauna to gather life history traits [32], to reveal cryptic diversity of coral-reef fishes [33, 34] and cave fishes [35], to identify farmed stocks of non-native species [36], and to guide conservation breeding [14], but the standard animal barcode marker cytochrome oxidase subunit I (*COI*) has not usually been sequenced for Malagasy freshwater fishes (but see [37]), and a comprehensive reference library is so far lacking. Here we present a curated database of 2860 mitochondrial sequences of the mitochondrial *COI*, *16S* and *ND2* genes, 1141 of which were newly sequenced for this data set, as a first step towards a DNA barcoding reference library of Madagascar’s fishes. The newly compiled data set allowed for (i) insights into levels of divergence, (ii) identification of groups of potentially overlooked diversity, (iii) a first assessment of mitochondrial relationships in the genus *Pachypanchax*, as well as (iv) information on the mitochondrial identity of introduced tilapias on the island.

## Materials and methods

The dataset compiled herein focuses on the mitochondrial gene for Cytochrome Oxidase Subunit 1 (*COI*) but also includes fragments of the mitochondrial gene for 16S rRNA (*16S*), and the mitochondrial gene for NADH-Dehydrogenase 2 (*ND2*). The latter two gene fragments were primarily used to verify species identification for samples in groups where reliably identified *COI* sequences from previous studies were not available; in particular tilapias (*ND2*) and native cichlids and bedotiids (*16S*). For *COI* and *16S*, we retrieved sequences from the nucleotide archive of GenBank combining the search term (i) “Madagascar” successively with (ii) “Actinopterygii”, “Chondrichthyes”, “Bedotiidae”, “Cichlidae”, and “*Pachypanchax*”, and (iii) “*COI*”, “*cox1*”, and “*16S*”. Sequences were downloaded as GenBank flatfiles and transformed into a tab-delimited file using DNAconvert as implemented in iTaxoTools [38]. Furthermore, we downloaded in tsv format one data set [35] from the BOLD data portal (https://www.boldsystems.org/) and added it to the table. This table was then curated in Microsoft Excel by (i) removing duplicates and sequences not matching the target fragments, (ii) removing all sequences not originating from Madagascar, (iii) merging rows for samples for which both *COI* and *16S* had been sequenced, (iv) merging geographic information from the "country" and "isolation_source" columns, and (v) updating taxonomy. For *ND2* we separately compiled a set of reliably identified reference sequences primarily from a comprehensive reference work [39] on tilapias which we used to match the sequences obtained from our Madagascar samples.

Own samples were collected during numerous field expeditions in Madagascar over the past 20 years, with an emphasis of a sampling of cichlids on the small island of Nosy Be, a sampling of various fishes in Manombo Special Reserve, and a large number of fishes from a transect spanning from the central highlands to the North West, Sambirano, North and North East regions (geographical regions herein are named following [40]).

All research methods reported in this paper complied with the guidelines for field research compiled by the American Society of Ichthyologists and Herpetologists (ASIH) and adhered to the legal requirements of Malagasy authorities. Approval for this study by an Institutional Animal Care and Use Committee (IACUC) was not required by Malagasy law. Approval of sampling procedures was included in the fieldwork permits issued by the Ministry of Environment, Direction of the System of Protected Areas (fieldwork permits N°238-MINENV.EF/SG/DGEF/DPB/SCBLF/RECH dated 22 December 2004 and 300/06/MINENV.EF/SG/DGEF/DPB/SCBLF/RECH dated 22 December 2006). A limited number of representative fish individuals were preserved as voucher specimens. These were sedated and subsequently euthanized by immersion in MS-222 solution, in compliance with the American Veterinary Medical Association guidelines. Subsequently, they were fixed in 95% ethanol, preserved in 70% ethanol. About 40 specimens from field campaigns in 2000–2004 were deposited in the Zoologische Staatssammlung München while specimens collected in 2011 were unfortunately lost. From other specimens obtained dead from local fishermen, fin clips were made. Tissue samples were preserved in 100% ethanol immediately upon collection, transported and stored without cooling under environmental temperatures for a variable amount of time (up to 6 months), and eventually stored at -20°C. The large number of samples collected between August and November 2011 were transported to the laboratory and processed for DNA extraction 1–4 months after collection. Most specimens during the field campaigns were digitally photographed, but photos from the 2011 campaign unfortunately were lost along with the voucher specimens collected in that year.

DNA was extracted from tissue samples using a salt protocol [41] and mitochondrial DNA fragments amplified using standard cycling conditions with the following primers: For the *COI* Folmer fragment we used dgLCO1490 (GGTCAACAAATCATAAAGAYATYGG) and dgHCO2198 (TAAACTTCAGGGTGACCAAARAAYCA) [42], or COI-Chmf4 (TYTCWACWAAYCAYAAAGAYATCGG) and COI-Chmr4 (ACYTCRGGRTGRCCRAARAATCA) [43]; for the 3’ fragment of the *16S* rRNA gene we used 16SA-L (5’-CGCCTGTTTATCAAAAACAT-3’) and 16SB-Hnew (5’-AGTCTGGCCTCATTAGGTCC-3’) modified from Palumbi et al. [44]; for *ND2* we used ND2Met (CATACCCCAAACATGTTGGT) and ND2Trp (GTSGSTTTTCACTCCCGCTTA) [45]. PCR products purified by using ExoSAP and directly sequenced on ABI capillary systems, with forward primers for *COI* and *16S*, and with both primers for *ND2*. Sequences were error-checked and trimmed in CodonCode Aligner (CodonCode Corp.), and added along with their metadata to the table with sequences retrieved from databases. No suspicious frameshifts or stop codons were identified that could characterize sequences as nuclear pseudogenes (NUMTs). All new sequences were submitted to GenBank (accession numbers ON584792‒ON585028, ON604004‒ON604633 and ON611849‒ON612106). The full data set with sequences and metadata in different spreadsheet formats, fasta files with all sequences per gene, and tree files, is available from Zenodo (https://doi.org/10.5281/zenodo.6792379). A basic spreadsheet with these data is provided as S1 Table.

Sequences were aligned using MAFFT [46], and exploratory maximum likelihood trees under the K2P substitution model constructed in MEGA7 [47]. Uncorrected pairwise distances between sequences were calculated using TaxI2, a program developed as part of the iTaxoTools project [38]. This distance measure was chosen over corrected distances (e.g., K2P) to ensure direct comparability with DNA barcoding results of other Malagasy vertebrates [25, 26]. Phylogenetic analysis of the full data sets were performed with FastTree [48], and average sequence lengths calculated with Concatenator, both implemented in iTaxoTools.

## Results and discussion

### A first step towards a comprehensive DNA barcode library for Madagascar’s ichthyofauna

The DNA barcoding database presented herein consists of 2015 *COI* sequences, 605 *16S* sequences, and 240 *ND2* sequences (Table 1). Average sequence length was 597 bp (range 245–700 bp) for *COI*, 540 bp (200–618 bp) for *16S*, and 614 bp (190–927 bp) for *ND2*. A total of 678 of these sequences are from introduced species, the remainder of native species. 1505 sequences are from freshwater fishes, 1193 sequences from marine fishes, and 162 from facultative freshwater inhabitants–this latter category includes anadromous and catadromous species as well as inhabitants of brackish waters and marine species that occasionally are found in freshwaters. The data set contains 419 species of 79 families (Table 1), of which 81 represent freshwater, 307 marine, and 31 facultative freshwater inhabitants. 207 samples and 19 species are chimaeras, rays and sharks, (Chondrichthyes), the remainder are ray-finned fishes (Actinopterygii). Newly obtained for this dataset were 630 *COI*, 271 *16S*, and 270 *ND2* sequences, all of them exclusively from obligate or facultative freshwater species, and the *ND2* sequences exclusively from tilapia samples. For these newly obtained sequences from own samples, amplification and sequencing success was very good, despite partly long storage. In 2020, we processed 92 samples; for 86 of these, DNA was newly extracted from the ethanol-preserved tissues that were collected between 2000 and 2006, while for another set of six samples collected in 2011 we used previous DNA extractions done in 2012. For *COI* (primers dgLCO1490 and dgHCO2198) 10 of the newly extracted samples failed in PCR (no or very weak bands on the agarose gel), while for *16S*, only five samples failed (PCR success rates of 89% and 95%, respectively). All successfully amplified samples yielded usable sequences.

**Table 1:** Counts of sequences in our DNA barcode library of Malagasy fishes for three mitochondrial DNA fragments (*COI*, *16S* and *ND2*).

Family	Environment	*COI*	*16S*	*ND2*
Anabantidae	F	2	2	0
Aplocheilidae	F	101	38	0
Arapaimidae	F	0	1	0
Bedotiidae	F	39	46	0
Channidae	F	39	4	0
Cichlidae	F	491	170	240
Cyprinidae	F	13	0	0
Milyeringidae	F	144	2	0
Poeciliidae	F	32	0	0
Ambassidae	FF	12	3	0
Anguillidae	FF	21	17	0
Atherinidae	FF	7	5	0
Clupeidae	FF	0	3	0
Gerreidae	FF	7	1	0
Kuhliidae	FF	7	6	0
Monodactylidae	FF	4	0	0
Mugilidae	FF	1	3	0
Scatophagidae	FF	0	1	0
Terapontidae	FF	0	1	0
Gobiidae	MF	132	17	0
Eleotridae	MF	58	24	0
Ariidae	M	0	1	0
Acanthuridae	M	16	0	0
Apogonidae	M	104	0	0
Balistidae	M	6	0	0
Blenniidae	M	30	2	0
Bothidae	M	1	0	0
Bythitidae	M	3	0	0
Caesionidae	M	12	0	0
Callionymidae	M	3	0	0
Carangidae	M	2	0	0
Carapidae	M	5	10	0
Carcharhinidae	M	134	0	0
Chaetodontidae	M	39	27	0
Chimaeridae	M	2	0	0
Chlopsidae	M	3	0	0
Cirrhitidae	M	8	0	0
Congridae	M	4	0	0
Haemulidae	M	9	0	0
Hemigaleidae	M	3	0	0
Holocentridae	M	10	1	0
Kyphosidae	M	1		0
Labridae	M	111	95	0
Leiognathidae	M	14	14	0
Lethrinidae	M	7	0	0
Lutjanidae	M	9	0	0
Megalopidae	M	0	1	0
Menidae	M	1	1	0
Monacanthidae	M	7	0	0
Mullidae	M	7	0	0
Muraenidae	M	1	0	0
Nemipteridae	M	15	0	0
Ophichthinae	M	1	1	0
Ostraciidae	M	2	0	0
Pempheridae	M	4	0	0
Pinguipedidae	M	4	0	0
Platycephalidae	M	4	0	0
Plesiopidae	M	1	0	0
Pomacanthidae	M	15	0	0
Pomacentridae	M	129	106	0
Priacantidae	M	2	0	0
Pseudochromidae	M	15	0	0
Rhinobatidae	M	1	0	0
Scaridae	M	10	0	0
Scorpaenidae	M	12	0	0
Serranidae	M	32	0	0
Siganidae	M	4	1	0
Sillaginidae	M	1	0	0
Soleidae	M	2	0	0
Sphyrnidae	M	64	0	0
Stegostomatidae	M	1	0	0
Syngnathidae	M	4	0	0
Synodontidae	M	11	1	0
Tetraodontidae	M	12	0	0
Torpedinidae	M	1	0	0
Triakidae	M	1	0	0
Tripterygiidae	M	4	0	0
Zanclidae	M	1	0	0
**Total**		**2015**	**605**	**240**

Environment is abbreviated as freshwater (F), facultative freshwater (FF), containing marine and freshwater species (MF), and marine (M). Note that the table contains both native and introduced species.

**Fig 1:**
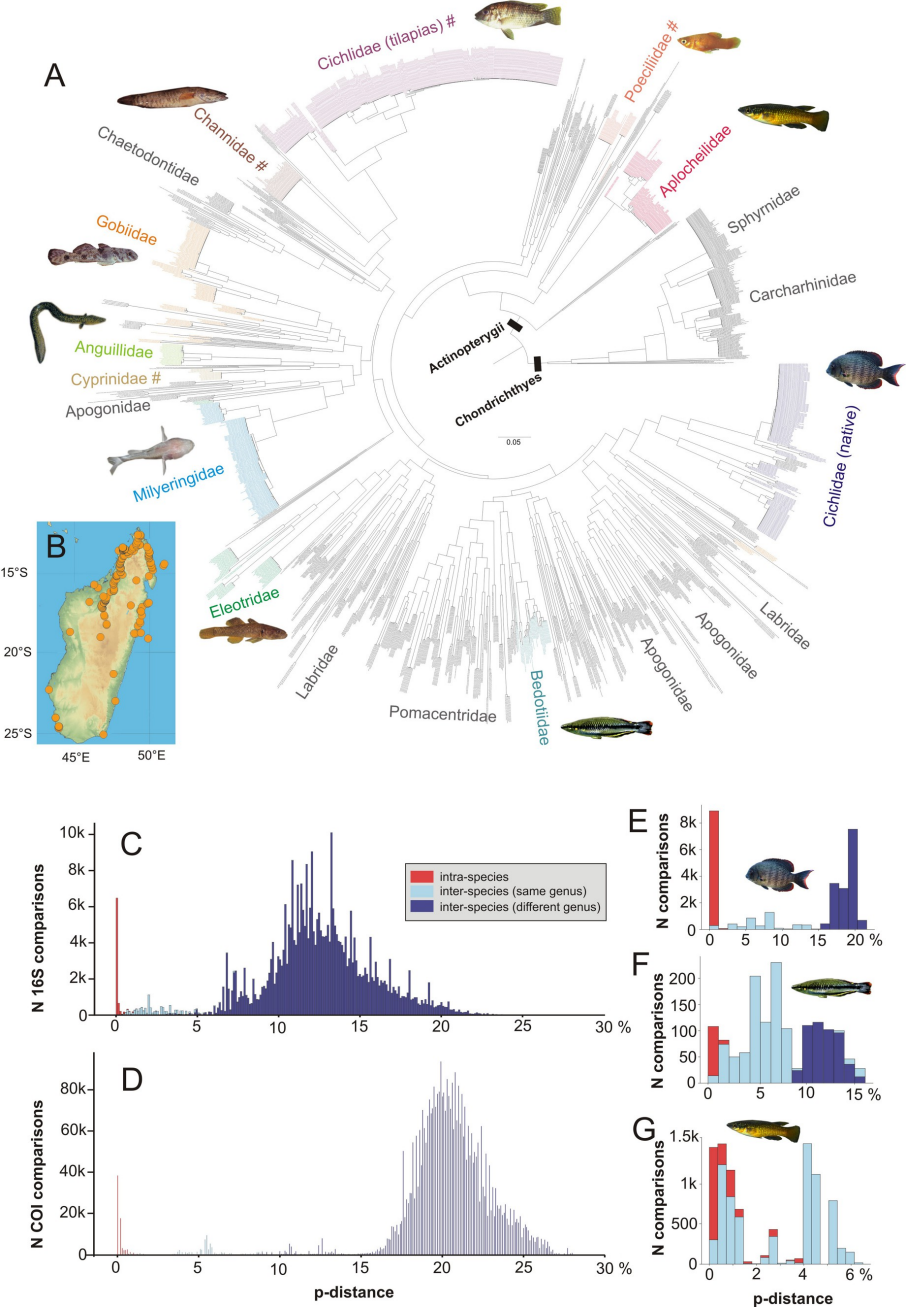
Taxonomic coverage of the *COI* barcode library for Malagasy fishes and genetic divergences. (A) Circle tree representation of an Approximate Maximum Likelihood tree calculated with FastTree from 2015 *COI* sequences. The most important and well represented families of obligate or facultative freshwater species are shown in color and with representative inset photos; for marine taxa, selected well-represented families are indicated in grey, but many additional species-poor families are not labelled; see S1 Fig. for a full representation of this tree in PDF format. (B) Map of Madagascar showing all sampling localities from which sequences were included in this study. Map colors represent elevations; drawn with the open-source Python library matplotlib/basemap (https://github.com/matplotlib/basemap). (C-G) Uncorrected pairwise distances (p-distances) in percent among samples of the same species (intra-species; red), samples from different species of the same genus (light blue) and different species of different genera (dark blue). Data are presented for (C) a fragment of the 16S gene from all available sequences for this gene fragment; (D) all available sequences for *COI*; (E) *COI* sequences of native Malagasy cichlids; (F) *COI* sequences of native Malagasy bedotiids; (G) *COI* sequences of native Malagasy aplocheilids.

Our database includes samples from 256 locations, and geographical coordinates for 172 of these (see inset map in Fig 1 for locations of georeferenced samples, and S3 Fig. for more detailed maps). It is important to mention that due to taxonomic uncertainties in groups lacking comprehensive revisions (especially Eleotridae and Gobiidae), in our library the assignment of some samples and species (all collected in freshwater habitats) to freshwater vs. facultative freshwater species is tentative. *COI* sequences of some groups were predominantly gathered from previous publications: marine fishes [33, 34, 49], endemic Malagasy cichlids [50], bedotiids [51], and typhleotrids [35].

Average uncorrected pairwise genetic distances in *COI* for the full data set were 20.6% (5.2‒31.0%) among species belonging to different genera, and 6.4% (0.0‒22.4%) for species belonging to the same genus. Considering only native Malagasy freshwater species, *COI* distances were 8.7‒24.9% for comparisons among non-congeneric species, 0.0–19.3% for comparisons among congeneric species, and 0.0‒4.6% for intraspecific comparisons. For *16S*, the respective values were 2.6‒22.7% among non-congenerics, 0.0‒8.4% among congenerics, and 0.0‒2.2% among conspecifics. The largest distances between congeneric sequences were found in the genus *Glossogobius*, while the largest distances within species were found between samples of *Ratsirakia* from two different sites that here were provisionally both assigned to *R*. *legendrei*. The minimal value of 0.0% distance between species corresponded to species of *Pachypanchax*, questioning some aspects of the current taxonomy, as will be discussed below; note that a specimen of *Typhleotris mararybe* with likely introgressed haplotype [35] was not included in this analysis. The *COI*/*16S* average distances between species (congeneric and non-congeneric) were 19.3% / 11.1% for species occurring in freshwater, 16.3% / 5.8% for native cichlids, 7.8% / 2.5% for bedotiids, and 3.1% / 1.2% for aplocheilids (genus *Pachypanchax*).

The new sequences assembled for this data set are informative regarding the diversity of Malagasy freshwater eleotrids and gobiids, for which so far almost no molecular resources were available. For instance, in the genus *Glossogobius* we found samples with sequences matching *G*. *giuris* in several locations in the North West and Sambirano regions of Madagascar, and samples matching *G*. *callidus* at one site. In addition, three deep lineages corresponding to other, unidentified *Glossogobius* species were found in several freshwater sites in Madagascar, one of which (from Bemarivo and Ankaramihely) was sister to the blind cave goby *G*. *ankaranensis* from Ankarana National Park, possibly representing a key finding to understand the subterraneous adaptations of this species. In the Eleotridae, we found two deep mitochondrial lineages in the obligate freshwater goby *Ratsirakia legendrei*, supporting that this species has a distinct phylogeographic structure and may represent a complex of various subspecies or species. The existence of additional species in the currently monotypic genus *Ratsirakia* has also been hypothesized previously [52] and mentioned in unpublished reports (e.g., [53]) that distinguished five evolutionary significant units within the genus, based primarily on morphological differentiation.

Our dataset also includes 70 *COI* and 68 *16S* sequences of the endemic cichlid *Ptychochromis oligacanthus*, and five sequences of *Paratilapia polleni*, from five crater lakes on the Malagasy offshore island of Nosy Be (Lakes Anjavibe, Amparihimirahavavy, Antsahamanavaka, Bempazava, Djabala) which showed no genetic variation (all sequences identical). The new data also allow for the first time an assessment of molecular variation in the killifish genus *Pachypanchax*, and a comprehensive and reliable assignment of Malagasy tilapia samples to mitochondrial lineages. The latter two aspects will be separately addressed in the subsequent sections.

### Mitochondrial identity of "tilapias" occurring in Madagascar

Cichlid fishes summarized under the common name tilapia comprise a series of genera of cichlid fishes which may represent a paraphyletic assemblage [54]. Tilapias are among the most important groups of farmed fishes globally, with over 7 million tonnes harvested in 2020 [55], and they have been introduced in Madagascar probably already in the late 19th century (*Oreochromis niloticus* [56]) where they represent a dramatic threat for native cichlid fishes by replacing them [16]. Introductions were intensified in the 1950s, and to date the following species have been reported from Madagascar [3]: *Coptodon rendalli*, *C*. *zillii*, *Oreochromis macrochir*, *O*. *mossambicus*, *O*. *niloticus*, *and O*. *spilurus*.

We used *ND2* sequences to match the identified mitochondrial lineages in our sampling to those of a comprehensive reference data set [39], and thereby assign *COI* and *16S* sequences of Malagasy tilapias to species. Our data expand a previous assessment [36] by demonstrating the presence of mitochondrial genomes of *O*. *aureus*, *O*. cf. *mossambicus*, *O*. *niloticus*, *C*. *rendallii* and *C*. *zillii*, as well as of one undetermined species (*Oreochromis* sp.) in wild Malagasy tilapia populations. The presence of *O*. *aureus* in Madagascar has not previously been reported [3] and this species was not included in the reference study [39]; however, our *ND2* sequences matched with 99.5% identity a sequence of that species from Lake Hula, Israel (accession DQ465029 [57]). Malagasy samples here considered as *O*. cf. *mossambicus* match sequences of *O*. *mossambicus* [39] with 99.7% in *ND2* and therefore almost certainly correspond to this species. Our own sampling did not yield any sequence of *O*. *macrochir* but the presence of this species in Madagascar is confirmed by sequences from another study [36]. Sequences of the undetermined *Oreochromis* sp. differ from all other species with reliable identification sequenced for *ND2* by at least 2.7% uncorrected *ND2* distance.

Our sampling revealed that at most surveyed sites in the North West of Madagascar, at least two mitochondrial tilapia lineages occur in syntopy. At some sites we found up to three syntopic lineages, e.g., syntopy of *O*. *aureus*, *O*. *niloticus* and *Oreochromis* sp. at an unnamed site (geographical coordinates -16.63653°, 47.08880°) and at Andranobevava (-17.12308°, 46.80760°); or sympatry of *O*. cf. *mossambicus*, *O*. *niloticus* and *C*. *zillii* at a site between Ambondromamy and Maevatanana (-16.67583°, 47.07433°). Interestingly, at sampling sites in the Sambirano region and in the North and North East, except one record of *Coptodon rendalli* at Nosy Be, only the undetermined species of *Oreochromis* (*O*. sp.) was found.

Because congeneric tilapias are known to hybridize [58, 59], we cannot determine if these co-occurrences, and our records in general, represent pure populations of the respective species, hybrids, or events of mitochondrial introgression in otherwise more or less pure populations of other species. The apparent presence of multiple introduced tilapia species in close spatial proximity highlights the prospect of studying phenomena of introgressive hybridization in these fishes in Madagascar, by combining genomic and morphological approaches.

It is intriguing that the undetermined *Oreochromis* sp. has not been reported in previous surveys and species lists of Madagascar’s freshwater fishes, despite its apparent high frequency at many sites according to our DNA barcoding data. On the contrary, we did not detect sequences of *Tilapia sparrmani*, a species previously listed for Madagascar [60] and considered to be common at some sites by Malagasy researchers [61], but not included in the most recent checklist of the island’s ichthyofauna [3]. Future studies should assess whether perhaps those apparently morphologically distinct tilapias from Madagascar that sometimes are identified as *T*. *sparrmani* may in fact correspond to the unidentified species of *Oreochromis* detected by the DNA barcodes in our study.

### Phylogeography and low mitochondrial divergences of Malagasy *Pachypanchax*

Madagascar’s bedotiids and endemic cichlids have been the subject of comprehensive molecular studies [49, 50] which were the basis for integrative taxonomic revision and descriptions of numerous new species. The third main clade of Malagasy freshwater fishes, the aplocheilid killifishes of the genus *Pachypanchax*, have been revised by Loiselle [62] but molecular data were so far only available for selected species [63]. Besides the Seychellean species *P*. *playfairii*, six species endemic to Madagascar are distinguished [62], occurring allopatrically in the North, North East, North West, and Sambirano regions of the island: *P*. *arnoulti*, *P*. *omalonotus*, *P*. *patriciae*, *P*. *sakaramy*, *P*. *sparksorum*, and *P*. *varatraza*. We here include 108 samples from 24 localities spanning the distribution areas of all these species, and furthermore extend the known distribution of *P*. *arnoulti* southwards with samples from Tsingy de Bemaraha National Park.

**Fig 2:**
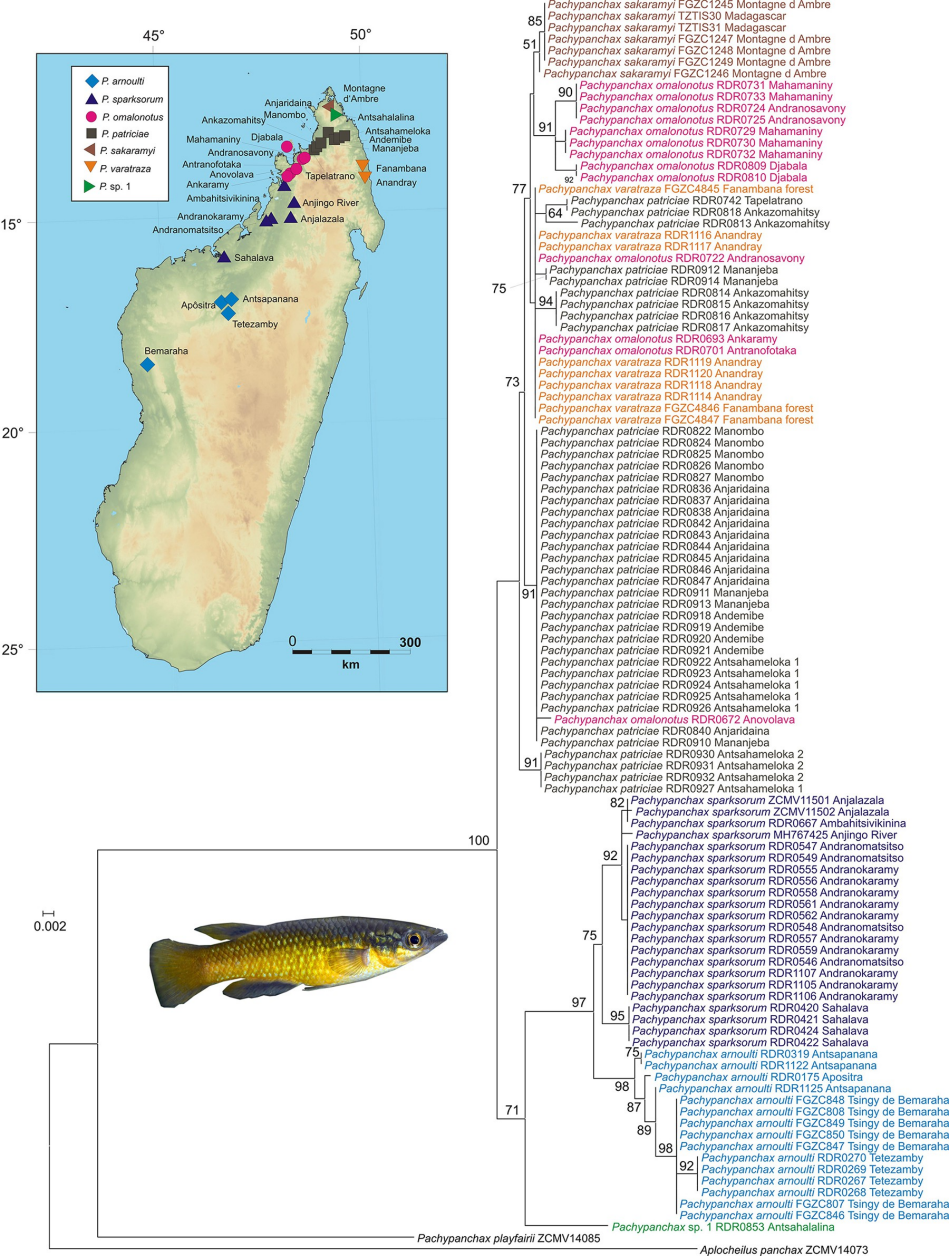
Geographic distribution of sample sites of Malagasy species of *Pachypanchax* (Aplocheilidae) from which sequences were available for this study, and mitochondrial Approximate Maximum Likelihood tree of combined *COI* and *16S* gene fragments (total alignment length 1199 nucleotides), calculated with FastTree. Numbers at nodes are SH-like local support values in percent (shown only if >50%). The inset shows a photo of *P*. *sakaramyi*. Map colors represent elevations; drawn with the open-source Python library matplotlib/basemap (https://github.com/matplotlib/basemap).

The mitochondrial data confirm that the Seychellean species, *P*. *playfairii*, is strongly divergent from the Malagasy *Pachypanchax*, with uncorrected genetic distances of 14.8–16.5% in *COI* and 7.8–11.5% in *16S*. In contrast, the Malagasy taxa had distinctly lower divergences among each other, with a highest *COI* distance of 6.5% between samples of *P*. *arnoulti* and *P*. *omalonotus*, and a highest *16S* distance of 2.8% between samples of *P*. *arnoulti* and *P*. *varatraza*. Phylogenetically, sequences from populations assigned to the two species of southernmost distribution (from the West and North West regions), *P*. *arnoulti* and *P*. *sparksorum*, form a monophyletic group (Fig 2). These two species are also reciprocally monophyletic if samples from Sahalava (-15.89892°, 46.58592°) close to the northern shore of the Betsiboka river are assigned to *P*. *sparksorum*; the *COI* genetic distances between these two species range from 2.1 to 3.1%, and the two species differ from the other Malagasy species by 4.0–6.5%.

The remaining species (*P*. *omalonotus*, *P*. *patriciae*, *P*. *sakaramyi*, *P*. *varatraza*) form a second clade. They had maximum differences of 1.4% and showed only a limited phylogeographic structure, with no reciprocal monophyly of *P*. *omalonotus*, *P*. *patriciae*, and *P*. *varatraza*. While the lack of voucher specimens and morphological data for the sequenced specimens precludes any taxonomic conclusions, it is probable that a certain level of gene flow and hybridization takes place, or has taken place, among at least three of the northern Malagasy species (*P*. *omalonotus*, *P*. *patriciae*, *P*. *varatraza*). Their status as separate species, as well as their precise ranges, require confirmation from nuclear-encoded markers, preferably from phylogenomic analyses of gene flow across hybrid zones.

Strikingly, the sole available sample from Antsahalalina (RDR 0853; -12.68143°, 49.2661°) was phylogenetically and genetically divergent from the other northern species. It differed from the other samples of *P*. *omalonotus*, *P*. *patriciae*, *P*. *sakaramyi* and *P*. *varatraza* by 3.3–4.3% *COI* distance, and in our preferred phylogenetic tree (Fig 2) was placed sister to the *P*. *arnoulti/sparksorum* clade. This population from an area near the southwestern edge of Montagne d’Ambre National Park should be the subject of new sampling as it may represent a new microendemic species.

### Perspectives

Madagascar’s native fishes require urgent conservation efforts, especially in freshwaters but also in marine environments [16, 21]. DNA barcoding can aid and inform such conservation management on various levels: (i) by revealing hitherto unknown genetic diversity, both at the population level and in terms of scientifically undescribed species, it guides taxonomic revision and defines the basic units meriting conservation actions; (ii) by enabling reliable assessments of distribution ranges it allows identification of centers of species richness and local endemism and defines areas and habitats requiring protection; (iii) by identifying captive populations it informs conservation breeding and reintroduction efforts [14]. The reference database presented herein represents a first step making DNA barcoding of Madagascar’s ichthyofauna widely applicable. By also including *16S* sequences for 187 fish species occurring in Madagascar, 88 of which from freshwater habitats, our library also provides a preliminary resource for DNA metabarcoding of environmental DNA (eDNA) samples, for which primers for *12S* and *16S* rDNA often perform better than those for *COI* [64].

As Malagasy freshwater fishes have evolved in a similar geographic setting as other vertebrates of the island, it is striking that their mitochondrial divergences appear to be lower than in co-occurring amphibians and reptiles. The average *COI* distances of 19.3% among all native freshwater species, and the 16.3% for cichlids, are at the same level as observed in the two other orders [25, 26]; however, the values for bedotiids (7.8%) and aplocheilids (3.1%) are distinctly lower than even in small and species-poor amphibian clades as the genus *Heterixalus* (12.7%) or reptile clades such as Malagasy boas (11.9%). This may indicate that the species status of some Malagasy fishes should be scrutinized (note that for bedotiids, we considered as species several lineages that have not yet been formally named and thus in any case require further study [51]).

Interestingly, compared to terrestrial vertebrates such as amphibians and reptiles [23–25] our data also did not reveal a large number of scientifically unnamed candidate species of fishes in Madagascar’s freshwaters. Besides the already known unnamed lineages of bedotiids [50] a few genetically divergent lineages possibly corresponding to new species were encountered in the Gobiidae and Eleotridae, two groups that have not yet been subjected to comprehensive taxonomic revisions in this century. Despite the limits in geographical sampling (mostly northern Madagascar) and field methodology (mostly hand nets in shallow water) of the surveys that yielded samples for our data base, it is striking that compared to the hundreds of deeply divergent lineages of amphibians and reptiles, less than ten such lineages were newly revealed by our data, suggesting that completion of the taxonomic inventory of Madagascar’s fishes is a realistic endeavour.

It is obvious that the current data set for freshwater fishes presented herein clearly has limitations, in particular due to the absence of voucher specimens of morphologically verified identity, or of photographies of the respective fish individual, for many of the new samples used–as such, the data set only partially fulfils the requirements of a reference library [65]. Nevertheless we are convinced that the identification of most *COI* sequences in the data set is reliable due to cross-checking of *16S* and *ND2* sequences obtained from the same samples with previously available sequences from reliably identified reference specimens (e.g., native cichlids, bedotiids, tilapias [39, 50, 51]). In other cases such as the allopatrically distributed species of *Pachypanchax*, geographic origin of the samples in itself allowed their identification according to current taxonomy. Our study therefore also is an example that highlights how DNA barcoding data can yield valuable information–such as refining distribution range information, elucidating intraspecific genetic variation, and identifying deviant genetic lineages towards which future research should be directed–in the absence of voucher specimens. Yet, we encourage future ichthyological inventory work in Madagascar to always include (i) high-resolution photography of all encountered individuals and submission of these photos to open-access public repositories along with detailed metadata, especially geographical coordinates, (ii) collection of representative voucher specimens for morphological study, and (iii) collection of tissue samples (e.g., fin clips) from multiple individuals, in order to extend and improve the reference library for these threatened animals and better understand their phylogeography and genetic variation.

Future surveys will also be valuable to better understand in depth the effects of past introductions of exotic fish species on Madagascar’s endemic ichthyofauna. It is well known that exotics have become dominant components of fish communities at most freshwater sites in Madagascar [12]. For instance, Moreau [66] documented ichthyofaunal changes in Lake Alaotra on Madagascar’s high plateau based on fishery reports: already in the early 1900s, goldfish (*Carassius auratus*) were detected, apparently in parallel with a decrease of *Paratilapia polleni* in fishermen catches; followed, in 1955, by the release of a tilapia identified as *Coptodon rendalli* which became dominant in the catches; and finally, the introduction of two further tilapias, purportedly *Oreochromis macrochir* and *O*. *niloticus*, that were introduced in this lake and rapidly replaced *C*. *rendalli* [66]. Exotic fish in Madagascar include predatory species such as largemouth bass (*Micropterus salmoides*), Nile arowana (*Heterotis niloticus*), and snakehead (*Channa striata*) [17], of which in particular snakehead have become widespread (detected at 22 sites in our survey) and probably contributed to declines and local extinctions of native species [67]. Furthermore, introduced poeciliids such as *Xiphophorus helleri* may prey heavily on eggs and larvae of native Malagasy fishes, and their occurrence seems to be linked to the disappearance of native bedotiids and aplocheiloids [17]. Although negative effects of the exotic species on Madagascar’s native ichthyofauna are obvious, it has been poorly explored how these introduced species interact with habitat degradation, and under which conditions they may coexist vs. completely displace the native species. In our survey, we detected poeciliids (*Xiphophorus helleri*, *X*. *maculatus*, *Gambusia holbrooki*, *Poecilia reticulata*) at 13 sites of which only three also hosted aplocheiloids (co-occurrence of *Pachypanchax arnoulti* with *X*. *helleri* at one site, and of *Pachypanchax arnoulti* repectively *Pachypanchax omalonotus* with *Poecilia reticulata* at two sites; see S1 Table). Future surveys combining the standardized assessment of habitat parameters with DNA barcoding or environmental DNA metabarcoding might contribute to identify ecological factors allowing the persistence of *Pachypanchax* at such sites despite the presence of poeciliids, and in general, of native fishes in the presence of exotics in Madagascar, as a basis for habitat management plans.

Besides collection of voucher specimens and samples, we recommend that future surveys should also consider the collection of living fish for the buildup of assurance populations in captivity, i.e., in breeding stations and zoos. Such breeding programs, ideally both in a coordinated in-country and international effort, would allow for potential restocking when required. Given the current rate of habitat destruction and diversity loss in Madagascar, it was already proposed nearly two decades ago that, for many fish species *ex situ* captive breeding represents the only reliable means to save them from extinction [16, 68]–despite priority should be given to habitat conservation. As important conservation centers with global network activities, zoos contribute a great amount by preserving viable species populations which would be threatened with extinction due to the increased loss in natural habitats in the wild [69]. Most recently, a review of threatened Malagasy freshwater fishes in zoos and aquaria [15] highlighted the necessity of an *ex situ* conservation network. This complies with the One Plan Approach proposed by the IUCN SSC Conservation Breeding Specialist Group (CBSG), viz. the development of management strategies and conservation actions by all responsible parties for all populations of a species, whether inside or outside their natural range [15].

## Supporting information

S1 Fig Full *COI* tree. Expanded version of Approximate Maximum Likelihood tree of Madagascar fishes as shown in Fig 1, calculated with FastTree from 2015 partial sequences of the mitochondrial *COI* gene, with full labels of all terminals.
(PDF)

S2 Fig Full *16S* tree. Approximate Maximum Likelihood tree of Madagascar fishes, calculated with FastTree from 605 partial sequences of the mitochondrial *16S* gene, with full labels of all terminals.
(PDF)

S3 Fig Maps of Madagascar with sampling localities. Maps show sampling locations of georeferenced samples in our DNA barcode library, separately from left to right for (i) sequences taken from Genbank, (ii) sequences derived from 2000–2004 field campaigns, and (iii) sequences derived from the 2011 field campaign. Map colors represent elevations; drawn with the open-source Python library matplotlib/basemap (https://github.com/matplotlib/basemap).
(JPG)

S1 Table Curated DNA barcode library for Madagascar’s ichthyofauna. Table with all sequences and associated metadata for the *COI*, *16S* and *ND2* gene fragments used in this study.
(XLSX)

## References

[1] Myers N, Mittermeier RA, Mittermeier CG, da Fonseca GA, Kent J. Biodiversity hotspots for conservation priorities. Nature. 2000; 403: 853–858. doi: 10.1038/35002501 10706275

[2] Goodman SM, Benstead JP (eds.). The Natural History of Madagascar. Chicago, IL; London: University of Chicago Press; 2003.

[3] Fricke R, Mahafina J, Behivoke F, Jaonalison H, Léopold M, Ponton D. Annotated checklist of the fishes of Madagascar, southwestern Indian Ocean, with 158 new records. FishTaxa. 2018; 3: 1–432.

[4] Stiassny MLJ, Raminosoa N. The fishes of the inland waters of Madagascar. In Teugels GG, Guégan JF, Albaret JJ, editors. Biological Diversity of African Fresh-and Brackish Water Fishes. Annales Sciences Zoologiques Tervuren 1994; 275: 133–149.

[5] Sparks J, Smith W. Freshwater fishes, dispersal ability, and nonevidence: “Gondwana life rafts” to the rescue. Syst Biol. 2005; 54: 158–165. doi: 10.1080/10635150590906019 15823966

[6] Chakrabarty P, Davis MP, Sparks JS. The first record of a trans-oceanic sister-group relationship between obligate vertebrate troglobites. PLoS One. 2012; 7: e44083. doi: 10.1371/journal.pone.0044083 22937155 PMC3429432

[7] Vences M, Freyhof J, Sonnenberg R, Kosuch J, Veith M. Reconciling fossils and molecules: Cenozoic divergence of cichlid fishes and the biogeography of Madagascar. J Biogeogr. 2001; 28: 1091–1099.

[8] Crottini A, Madsen O, Poux C, Strauß A, Vieites DR, Vences M. Vertebrate time-tree elucidates the biogeographic pattern of a major biotic change around the K-T boundary in Madagascar. Proc Natl Acad Sci USA. 2012; 109: 5358–5363. doi: 10.1073/pnas.1112487109 22431616 PMC3325728

[9] Near TJ, Eytan RI, Dornburg A, Kuhn KL, Moore JA, Davis MP, et al. Resolution of ray-finned fish phylogeny and timing of diversification. Proc Natl Acad Sci USA. 2012; 109: 13698–13703. doi: 10.1073/pnas.1206625109 22869754 PMC3427055

[10] Friedman M, Keck BP, Dornburg A, Eytan RI, Martin CH, Hulsey CD, et al. Molecular and fossil evidence place the origin of cichlid fishes long after Gondwanan rifting. Proc Roy Soc Lond B. 2013; 280: 20131733. doi: 10.1098/rspb.2013.1733 24048155 PMC3779330

[11] Matschiner M, Musilová Z, Barth JM, Starostová Z, Salzburger W, Steel M, et al. Bayesian phylogenetic estimation of clade ages supports trans-Atlantic dispersal of cichlid fishes. Syst Biol. 2017; 66: 3–22. doi: 10.1093/sysbio/syw076 28173588

[12] Sparks JS, Stiassny MLJ. Introduction to the freshwater fishes. In: Goodman SM, Benstead JP, editors. The Natural History of Madagascar. University of Chicago Press; 2003. pp. 849–863.

[13] Kiener A, Richard-Vindard G. Fishes of the continental waters of Madagascar. Pp. 477–499 in Battistini R, Richard-Vindard G (eds). Biogeography and Ecology of Madagascar. W. Junk, The Hague (Netherlands); 1972.

[14] Ziegler T, Frank-Klein N, Ommer S, Hürche R, Loiselle PV, Vences M. Keeping and breeding of threatened endemic Malagasy freshwater fishes at Cologne Zoo (Germany): a contribution towards the advancement of a conservation breeding network. Der Zoologische Garten. 2020; 88: 123–155.

[15] Leiss L, Rauhaus A, Rakotoarison A, Fusari C, Vences M, Ziegler T. Review of threatened Malagasy freshwater fishes in zoos and aquaria: The necessity of an ex situ conservation network—A call for action. Zoo Biol. 2022; 41: 244–262; doi: 10.1002/zoo.21661 34870879 PMC9299897

[16] Benstead JP, De Rham P, Gattolliat JL, Gibon FM, Loiselle PV, Sartori M, et al. Conserving Madagascar’s freshwater biodiversity. BioScience. 2003; 53: 1101–1111.

[17] Ravelomanana T, Máiz-Tomé L, Darwall W, Sayer C, Sparks J. The status and distribution of freshwater fishes. In: Máiz-Tomé L, Sayer C, Darwall W, editors. The Status and Distribution of Freshwater Biodiversity in Madagascar and the Indian Ocean Islands Hotspot. Gland, Switzerland: IUCN; 2018. pp. 13–28. doi: 10.1684/mst.2018.0776

[18] Styger E, Rakotondramasy HM, Pfeffer JM, Fernandes ECM, Bates DM. 2007. Influence of slash-and-burn farming practices on fallow succession and land degradation in the rainforest region of Madagascar. Agric., Ecosyst. Environ. 2007; 119: 257–269.

[19] Kull C.A. Air photo evidence of land cover change in the highlands: wetlands and grasslands give way to crops and woodlots. Madagascar Conserv Dev. 2012; 7: 144–152.

[20] IUCN. The IUCN Red List of Threatened Species. Version 2020–1. https://www.iucnredlist.org; 2020. Downloaded on 19 March 2020.

[21] Harris AR. Out of sight but no longer out of mind: a climate of change for marine conservation in Madagascar. Madagascar Conserv Dev. 2011; 6: 7–14.

[22] Cooke A, Brand J. Madagascar–A Guide to Marine Biodiversity. Editions Resolve. 2012; 176 pp.

[23] Smith MA, Fisher BL, Hebert PD. DNA barcoding for effective biodiversity assessment of a hyperdiverse arthropod group: the ants of Madagascar. Phil Trans Roy Soc Lond B. 2005; 360: 1825–1834. 10.1098/rstb.2005.1714 16214741 PMC1609228

[24] Vieites DR, Wollenberg KC, Andreone F, Köhler J, Glaw F, Vences M. Vast underestimation of Madagascar’s biodiversity evidenced by an integrative amphibian inventory. Proc Natl Acad Sci USA. 2009; 106: 8267–8272. doi: 10.1073/pnas.0810821106 19416818 PMC2688882

[25] Nagy ZT, Sonet G, Glaw F, Vences M. First large-scale DNA barcoding assessment of reptiles in the biodiversity hotspot of Madagascar, based on newly designed COI primers. PLoS One. 2012; 7: e34506. doi: 10.1371/journal.pone.0034506 22479636 PMC3316696

[26] Perl RGB, Nagy ZT, Sonet G, Glaw F, Wollenberg KC, Vences M. DNA barcoding Madagascar’s amphibian fauna. Amphibia-Reptilia. 2014; 35: 197–206.

[27] Hassold S, Lowry PP 2nd, Bauert MR, Razafintsalama A, Ramamonjisoa L, Widmer A. DNA barcoding of Malagasy rosewoods: towards a molecular identification of CITES-listed *Dalbergia* species. PloS One. 2016; 11: e0157881. doi: 10.1371/journal.pone.0157881 27362258 PMC4928830

[28] Lopez-Vaamonde C, Sire L, Rasmussen B, Rougerie R, Wieser C, Allaoui AA, et al. DNA barcodes reveal deeply neglected diversity and numerous invasions of micromoths in Madagascar. Genome. 2019; 62: 108–121. doi: 10.1139/gen-2018-0065 30184444

[29] Becker S, Hanner R, Steinke D. Five years of FISH-BOL: brief status report. Mitochondrial DNA. 2013; 22 Suppl 1: 3–9. 10.3109/19401736.2010.535528 21271850

[30] Ward RD. FISH-BOL, a case study for DNA barcodes. Methods in Molecular Biology (Clifton, N.J.). 2012; 858: 423–439. doi: 10.1007/978-1-61779-591-6_21 22684969

[31] Filonzi L, Vaghi M, Ardenghi A, Rontani PM, Voccia A, Nonnis Marzano F. Efficiency of DNA mini-barcoding to assess mislabeling in commercial fish products in Italy: an overview of the last decade. Foods. 2021; 10: 1449. doi: 10.3390/foods10071449 34206502 PMC8305242

[32] Raharinaivo LR, Jaonalison H, Mahafina J, Ponton D. How to efficiently determine the size at maturity of small-sized tropical fishes: A case study based on 144 species identified via DNA barcoding from southwestern Madagascar. J Appl Ichthyol 2020; 36: 402–413.

[33] Hubert N, Paradis E, Bruggemann H, Planes S. Community assembly and diversification in Indo-Pacific coral reef fishes. Ecol Evol. 2011; 1: 229–277. doi: 10.1002/ece3.19 22393499 PMC3287318

[34] Hubert N, Meyer CP, Bruggemann HJ, Guérin F, Komeno RJ, Espiau B, et al. Cryptic diversity in Indo-Pacific coral-reef fishes revealed by DNA-barcoding provides new support to the centre-of-overlap hypothesis. PloS One. 2012; 7: e28987. doi: 10.1371/journal.pone.0028987 22438862 PMC3305298

[35] Vences M, Rasoloariniaina JR, Riemann JC. A preliminary assessment of genetic divergence and distribution of Malagasy cave fish in the genus *Typhleotris* (Teleostei: Milyeringidae). Zootaxa. 2018; 4378: 367–376. doi: 10.11646/zootaxa.4378.3.5 29690007

[36] Hubert N, Pepey E, Mortillaro JM, Steinke D, Andria-Mananjara DE, de Verdal H (2021). Mitochondrial genetic diversity among farmed stocks of *Oreochromis* spp. (perciformes, cichlidae) in Madagascar. Diversity. 2021; 13: 1–11. 10.3390/d13070281

[37] Rakotomamonjy L, Oliarinony R. Phylogenetic, morphological and systematic studies of three genera of Cichlidae (Teleosts fish, Perciformes) endemic of Madagascar. Biodiversity Int J. 2018; 2: 538–543.

[38] Vences M, Miralles A, Brouillet S, Ducasse J, Fedosov A, Kharchev V, et al. iTaxoTools 0.1: Kickstarting a specimen-based software toolkit for taxonomists. Megataxa. 2021; 66: 77–92. 10.11646/megataxa.6.2.1

[39] Klett V, Meyer A. What, if anything, is a Tilapia?—Mitochondrial ND2 phylogeny of tilapiines and the evolution of parental care systems in the African cichlid fishes. Mol Biol Evol. 2002; 19: 865–883. doi: 10.1093/oxfordjournals.molbev.a004144 12032243

[40] Boumans L, Vieites DR, Glaw F, Vences M. Geographical patterns of deep mitochondrial differentiation in widespread Malagasy reptiles. Mol Phylogenet Evol. 2007; 45: 822–839. doi: 10.1016/j.ympev.2007.05.028 17920299

[41] Bruford MW, Hanotte O, Brookfield JFY, Burke T. Single-locus and multilocus DNA fingerprinting. In: Hoelzel AR, editor. Molecular Genetic Analysis of Populations: A Practical Approach. 1992; IRL Press, Oxford. pp. 225–270.

[42] Folmer O, Black M, Hoeh W, Lutz R, Vrijenhoek R. DNA primers for amplification of mitochondrial cytochrome c oxidase subunit I from diverse metazoan invertebrates. Mol Mar Biol Biotechn. 1994; 3: 294–299. 10.1071/ZO9660275 7881515

[43] Che J, Chen HM, Jin JQ, Joang K, Yuan ZY, Murphy RW, et al. Universal COI primers for DNA barcoding amphibians. Mol Ecol Res. 2012; 12: 247–258. doi: 10.1111/j.1755-0998.2011.03090.x 22145866

[44] Palumbi S, Martin A, Ramano S, McMillan WO, Stice L, Grabowski G. The Simple Fool’s Guide to PCR, Version 2. University of Hawaii Zoology Department, Honolulu, Hawaii; 1991.

[45] Kocher TD, Conroy JA, McKaye KR, Srauffer JR, Lockwood SF. Evolution of NADH dehydro-genase subunit 2 in East African cichlid fish. Mol Phylogenet Evol. 1995; 4: 420–432. doi: 10.1006/mpev.1995.1039 8747298

[46] Katoh K, Standley DM. MAFFT multiple sequence alignment software version 7: improvements in performance and usability. Mol Biol Evol. 2013; 30: 772–780. doi: 10.1093/molbev/mst010 23329690 PMC3603318

[47] Kumar S, Stecher G, Tamura K. MEGA7: Molecular Evolutionary Genetics Analysis Version 7.0 for Bigger Datasets. Mol Biol Evol. 2016; 33: 1870–1874. doi: 10.1093/molbev/msw054 27004904 PMC8210823

[48] Price MN, Dehal PS, Arkin AP. FastTree 2—Approximately Maximum-Likelihood trees for large alignments. PLoS One. 2010; 5: e9490. doi: 10.1371/journal.pone.0009490 20224823 PMC2835736

[49] Doukakis P, Hanner R, Shivji M, Bartholomew C, Chapman D, Wong E, Amato G. Applying genetic techniques to study remote shark fisheries in northeastern Madagascar. Mitochondrial DNA. 2011; 22: 15–20. doi: 10.3109/19401736.2010.526112 21281222

[50] Sparks JS. Molecular phylogeny and biogeography of the Malagasy and South Asian cichlids (Teleostei: Perciformes: Cichlidae). Mol Phylogenet Evol. 2004; 30: 599–614. doi: 10.1016/S1055-7903(03)00225-2 15012941

[51] Sparks JS, Smith WL. Phylogeny and biogeography of the Malagasy and Australasian rainbowfishes (Teleostei: Melanotaenioidei): Gondwanan vicariance and evolution in freshwater Mol Phylogenet Evol. 2004; 33: 719–734. doi: 10.1016/j.ympev.2004.07.002 15522799

[52] Dickinson S, Berner PO. Ambatovy project: Mining in a challenging biodiversity setting in Madagascar. In: Biodiversity, exploration, and conservation of the natural habitats associated with the Ambatovy project, eds. Goodman SM, Mass V. Malagasy Nature. 2010; 3: 2–13.

[53] Ambatovy. Ambatovy Sustainability Report 2017. Ambatovy (Sumitomo Corp., Kores, Sherritt). Antananarivo, Madagascar, 2017.

[54] Dunz AR, Schliewen UK. Molecular phylogeny and revised classification of the haplotilapiine cichlid fishes formerly referred to as “Tilapia.” Mol Phylogenet Evol. 2013; 68: 64–80. doi: 10.1016/j.ympev.2013.03.015 23542002

[55] FAO. Tilapia sector expected to resume rapid growth after temporary slowdown in 2020 _ GLOBEFISH _ Food and Agriculture Organization of the United Nations.pdf. 2021; https://Www.Fao.Org/in-Action/Globefish/Market-Reports/Resource-Detail/En/c/1379264/. https://www.fao.org/in-action/globefish/market-reports/resource-detail/en/c/1379264/

[56] Bleeker P. Poissons de Madagascar et de Île de la Réunion des collections de MM. Pollen et van Dam. Recherches sur la Faune de Madagascar et de ses Dépendances. 1874; 4: 1–104. https://www.biodiversitylibrary.org/part/74282

[57] Cnaani A, Lee BY, Zilberman N, Ozouf-Costaz C, Hulata G, Ron M, et al. Genetics of sex determination in tilapiine species. Sex Dev. 2008; 2: 43–54. doi: 10.1159/000117718 18418034

[58] Taniguchi N, Macaranas JM, Pullin RSV. Introgressive hybridization in cultured tilapia stocks in the Philippines. Bull Japan Soc Sci Fisheries. 1985; 51: 1219–1224.

[59] D’Amato ME, Esterhuyse MM, van der Waal BCW, Brink D, Volckaert FAM. Hybridization and phylogeography of the Mozambique tilapia *Oreochromis mossambicus* in southern Africa evidenced by mitochondrial and microsatellite DNA genotyping. Conserv Genet. 2007; 8: 475–488. 10.1007/s10592-006-9186-x

[60] Reinthal PN, Stiassny MLJ. The freshwater fishes of Madagascar: a study of an endangered fauna with recommendations for a conservation strategy. Cons Biol. 1991, 5: 231–243.

[61] Andriantsoaharinanahary R. Étude des elements fondamentaux ecosystemiques du Grand Lac du Parc Botanique et Zoologique de Tsimbazaza, Antananarivo, en vue de la conservation de *Paratilapia* sp. (Poisson-Cichlidae) endemique de Madagascar. Unpublished diploma thesis, Université d’Antananarivo. 2015.

[62] Loiselle PV. A review of the Malagasy *Pachypanchax* (Teleostei: Cyprinodontiformes, Aplocheilidae), with descriptions of four new species. Zootaxa. 2006; 1366: 1–44.

[63] Pohl M, Milvertz FC, Meyer A, Vences M. Multigene phylogeny of cyprinodontiform fishes suggests continental radiations and a rogue taxon position of *Pantanodon*. Vertebrate Zool. 2015; 65: 37–44.

[64] Shu L, Ludwig A, Peng Z. Environmental DNA metabarcoding primers for freshwater fish detection and quantification: In silico and in tanks. Ecol Evol. 2021; 11: 8281–8294. doi: 10.1002/ece3.7658 34188886 PMC8216916

[65] Rimet F, Aylagas E, Borja A, Bouchez A, Canino A, Chauvin C, et al. Metadata standards and practical guidelines for specimen and DNA curation when building barcode reference libraries for aquatic life. Metabarcoding and Metagenomics. 2021; 5: e58056. 10.3897/mbmg.5.58056

[66] Moreau J. Le lac Alaotra à Madagascar: cinquante ans d’aménagement des pêches. Cahiers de l’ORSTOM, Série Hydrobiologie. 1979; 13: 171–179.

[67] Raminosoa NR. Écologie et biologie d’un poison teleosteen: *Ophiocephalus striatus* (Bloch, 1793), introduit à Madagascar. Master’s thesis. University of Madagascar,Antananarivo. 1987.

[68] Loiselle PV. Captive breeding for the freshwater fishes of Madagascar. In: Goodman SM, Benstead JP, editors. The Natural History of Madagascar. Chicago: University of Chicago Press. 2003; pp. 1569–1574.

[69] Conde DA. The Role of Zoos. Grzimek’s Animal Life Encyclopedia. Cengange Learning; 2013.

