# Supplementary material for: Towards a DNA barcode library for Madagascar’s threatened ichthyofauna
Source: PLoS One. 2022 Aug 11;17(8):e0271400. doi: 10.1371/journal.pone.0271400 (PMC9371263; doi:10.1371/journal.pone.0271400)
Supplement: S1 Fig — Expanded version of Approximate Maximum Likelihood tree of Madagascar fishes as shown in Fig 1, calculated with FastTree from 2015 partial sequences of the mitochondrial COI gene, with full labels of all terminals. (PDF) [file pone.0271400.s001.pdf]

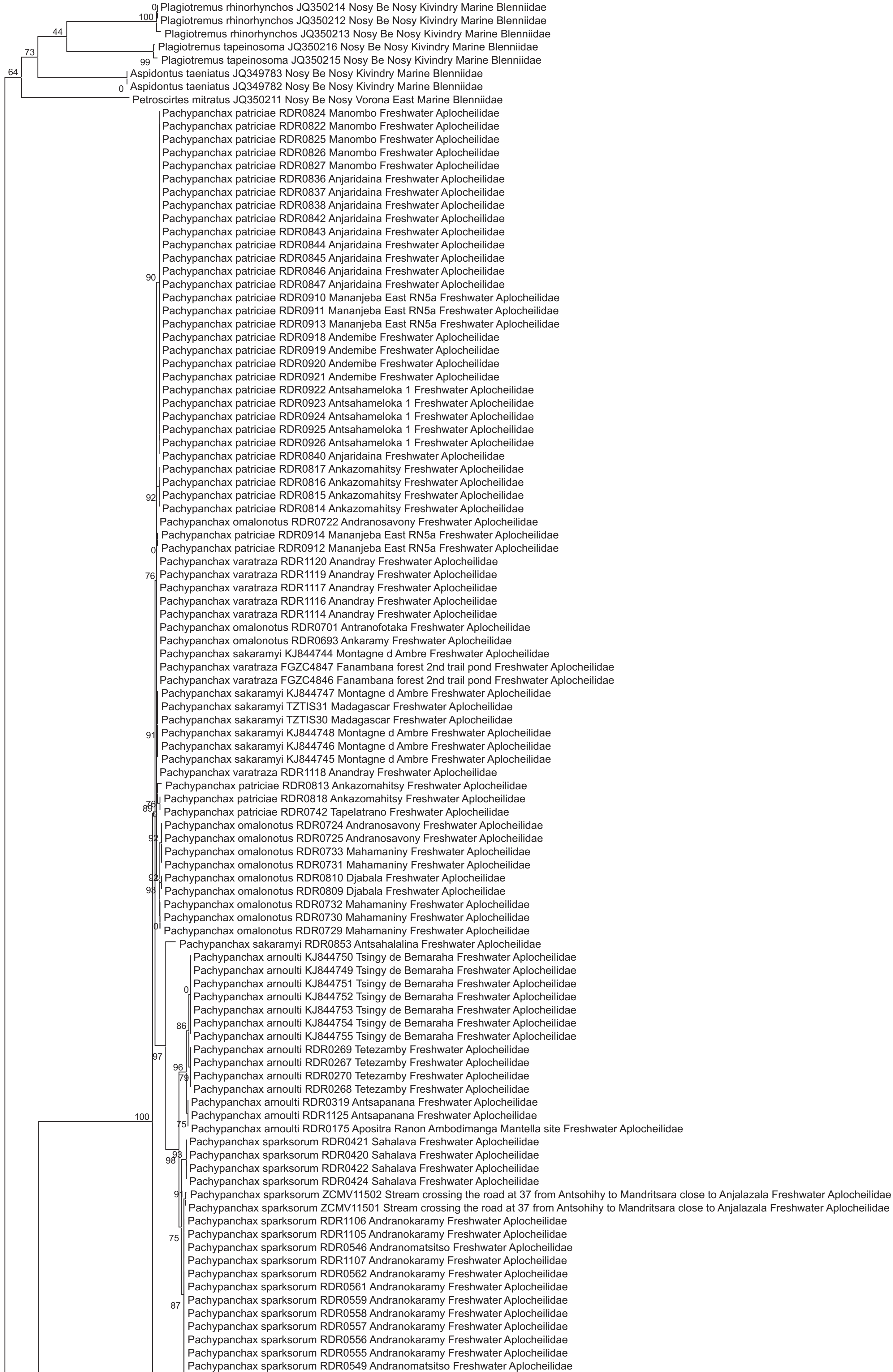

0
100
Plagiotremus rhinorhynchos JQ350214 Nosy Be Nosy Kivindry Marine Blenniidae
Plagiotremus rhinorhynchos JQ350212 Nosy Be Nosy Kivindry Marine Blenniidae
44
Plagiotremus rhinorhynchos JQ350213 Nosy Be Nosy Kivindry Marine Blenniidae
73
Plagiotremus tapeinosoma JQ350216 Nosy Be Nosy Kivindry Marine Blenniidae
99
Plagiotremus tapeinosoma JQ350215 Nosy Be Nosy Kivindry Marine Blenniidae
64
Aspidontus taeniatus JQ349783 Nosy Be Nosy Kivindry Marine Blenniidae
0
Aspidontus taeniatus JQ349782 Nosy Be Nosy Kivindry Marine Blenniidae
Petroscirtes mitratus JQ350211 Nosy Be Nosy Vorona East Marine Blenniidae
Pachypanchax patriciae RDR0824 Manombo Freshwater Aplocheilidae
Pachypanchax patriciae RDR0822 Manombo Freshwater Aplocheilidae
Pachypanchax patriciae RDR0825 Manombo Freshwater Aplocheilidae
Pachypanchax patriciae RDR0826 Manombo Freshwater Aplocheilidae
Pachypanchax patriciae RDR0827 Manombo Freshwater Aplocheilidae
Pachypanchax patriciae RDR0836 Anjaridaina Freshwater Aplocheilidae
Pachypanchax patriciae RDR0837 Anjaridaina Freshwater Aplocheilidae
Pachypanchax patriciae RDR0838 Anjaridaina Freshwater Aplocheilidae
Pachypanchax patriciae RDR0842 Anjaridaina Freshwater Aplocheilidae
Pachypanchax patriciae RDR0843 Anjaridaina Freshwater Aplocheilidae
Pachypanchax patriciae RDR0844 Anjaridaina Freshwater Aplocheilidae
Pachypanchax patriciae RDR0845 Anjaridaina Freshwater Aplocheilidae
Pachypanchax patriciae RDR0846 Anjaridaina Freshwater Aplocheilidae
90
Pachypanchax patriciae RDR0847 Anjaridaina Freshwater Aplocheilidae
Pachypanchax patriciae RDR0910 Mananjeba East RN5a Freshwater Aplocheilidae
Pachypanchax patriciae RDR0911 Mananjeba East RN5a Freshwater Aplocheilidae
Pachypanchax patriciae RDR0913 Mananjeba East RN5a Freshwater Aplocheilidae
Pachypanchax patriciae RDR0918 Andemibe Freshwater Aplocheilidae
Pachypanchax patriciae RDR0919 Andemibe Freshwater Aplocheilidae
Pachypanchax patriciae RDR0920 Andemibe Freshwater Aplocheilidae
Pachypanchax patriciae RDR0921 Andemibe Freshwater Aplocheilidae
Pachypanchax patriciae RDR0922 Antsahameloka 1 Freshwater Aplocheilidae
Pachypanchax patriciae RDR0923 Antsahameloka 1 Freshwater Aplocheilidae
Pachypanchax patriciae RDR0924 Antsahameloka 1 Freshwater Aplocheilidae
Pachypanchax patriciae RDR0925 Antsahameloka 1 Freshwater Aplocheilidae
Pachypanchax patriciae RDR0926 Antsahameloka 1 Freshwater Aplocheilidae
Pachypanchax patriciae RDR0840 Anjaridaina Freshwater Aplocheilidae
Pachypanchax patriciae RDR0817 Ankazomahitsy Freshwater Aplocheilidae
Pachypanchax patriciae RDR0816 Ankazomahitsy Freshwater Aplocheilidae
92
Pachypanchax patriciae RDR0815 Ankazomahitsy Freshwater Aplocheilidae
Pachypanchax patriciae RDR0814 Ankazomahitsy Freshwater Aplocheilidae
Pachypanchax omalonotus RDR0722 Andranosavony Freshwater Aplocheilidae
Pachypanchax patriciae RDR0914 Mananjeba East RN5a Freshwater Aplocheilidae
0
Pachypanchax patriciae RDR0912 Mananjeba East RN5a Freshwater Aplocheilidae
Pachypanchax varatraza RDR1120 Anandray Freshwater Aplocheilidae
76
Pachypanchax varatraza RDR1119 Anandray Freshwater Aplocheilidae
Pachypanchax varatraza RDR1117 Anandray Freshwater Aplocheilidae
Pachypanchax varatraza RDR1116 Anandray Freshwater Aplocheilidae
Pachypanchax varatraza RDR1114 Anandray Freshwater Aplocheilidae
Pachypanchax omalonotus RDR0701 Antranofotaka Freshwater Aplocheilidae
Pachypanchax omalonotus RDR0693 Ankaramy Freshwater Aplocheilidae
Pachypanchax sakaramyi KJ844744 Montagne d Ambre Freshwater Aplocheilidae
Pachypanchax varatraza FGZC4847 Fanambana forest 2nd trail pond Freshwater Aplocheilidae
Pachypanchax varatraza FGZC4846 Fanambana forest 2nd trail pond Freshwater Aplocheilidae
Pachypanchax sakaramyi KJ844747 Montagne d Ambre Freshwater Aplocheilidae
Pachypanchax sakaramyi TZTIS31 Madagascar Freshwater Aplocheilidae
Pachypanchax sakaramyi TZTIS30 Madagascar Freshwater Aplocheilidae
91
Pachypanchax sakaramyi KJ844748 Montagne d Ambre Freshwater Aplocheilidae
Pachypanchax sakaramyi KJ844746 Montagne d Ambre Freshwater Aplocheilidae
Pachypanchax sakaramyi KJ844745 Montagne d Ambre Freshwater Aplocheilidae
Pachypanchax varatraza RDR1118 Anandray Freshwater Aplocheilidae
Pachypanchax patriciae RDR0813 Ankazomahitsy Freshwater Aplocheilidae
Pachypanchax patriciae RDR0818 Ankazomahitsy Freshwater Aplocheilidae
Pachypanchax patriciae RDR0742 Tapelatrano Freshwater Aplocheilidae
Pachypanchax omalonotus RDR0724 Andranosavony Freshwater Aplocheilidae
Pachypanchax omalonotus RDR0725 Andranosavony Freshwater Aplocheilidae
Pachypanchax omalonotus RDR0733 Mahamaniny Freshwater Aplocheilidae
Pachypanchax omalonotus RDR0731 Mahamaniny Freshwater Aplocheilidae
Pachypanchax omalonotus RDR0810 Djabala Freshwater Aplocheilidae
Pachypanchax omalonotus RDR0809 Djabala Freshwater Aplocheilidae
Pachypanchax omalonotus RDR0732 Mahamaniny Freshwater Aplocheilidae
Pachypanchax omalonotus RDR0730 Mahamaniny Freshwater Aplocheilidae
0
Pachypanchax omalonotus RDR0729 Mahamaniny Freshwater Aplocheilidae
Pachypanchax sakaramyi RDR0853 Antsahalalina Freshwater Aplocheilidae
Pachypanchax arnoulti KJ844750 Tsingy de Bemaraha Freshwater Aplocheilidae
Pachypanchax arnoulti KJ844749 Tsingy de Bemaraha Freshwater Aplocheilidae
Pachypanchax arnoulti KJ844751 Tsingy de Bemaraha Freshwater Aplocheilidae
0
Pachypanchax arnoulti KJ844752 Tsingy de Bemaraha Freshwater Aplocheilidae
Pachypanchax arnoulti KJ844753 Tsingy de Bemaraha Freshwater Aplocheilidae
86
Pachypanchax arnoulti KJ844754 Tsingy de Bemaraha Freshwater Aplocheilidae
Pachypanchax arnoulti KJ844755 Tsingy de Bemaraha Freshwater Aplocheilidae
Pachypanchax arnoulti RDR0269 Tetezamby Freshwater Aplocheilidae
97
96
Pachypanchax arnoulti RDR0267 Tetezamby Freshwater Aplocheilidae
79
Pachypanchax arnoulti RDR0270 Tetezamby Freshwater Aplocheilidae
Pachypanchax arnoulti RDR0268 Tetezamby Freshwater Aplocheilidae
Pachypanchax arnoulti RDR0319 Antsapanana Freshwater Aplocheilidae
100
Pachypanchax arnoulti RDR1125 Antsapanana Freshwater Aplocheilidae
75
Pachypanchax arnoulti RDR0175 Apositra Ranon Ambodimanga Mantella site Freshwater Aplocheilidae
Pachypanchax sparksorum RDR0421 Sahalava Freshwater Aplocheilidae
98
Pachypanchax sparksorum RDR0420 Sahalava Freshwater Aplocheilidae
Pachypanchax sparksorum RDR0422 Sahalava Freshwater Aplocheilidae
Pachypanchax sparksorum RDR0424 Sahalava Freshwater Aplocheilidae
91
Pachypanchax sparksorum ZCMV11502 Stream crossing the road at 37 from Antsohihy to Mandritsara close to Anjalazala Freshwater Aplocheilidae
Pachypanchax sparksorum ZCMV11501 Stream crossing the road at 37 from Antsohihy to Mandritsara close to Anjalazala Freshwater Aplocheilidae
Pachypanchax sparksorum RDR1106 Andranokaramy Freshwater Aplocheilidae
75
Pachypanchax sparksorum RDR1105 Andranokaramy Freshwater Aplocheilidae
Pachypanchax sparksorum RDR0546 Andranomatsitso Freshwater Aplocheilidae
Pachypanchax sparksorum RDR1107 Andranokaramy Freshwater Aplocheilidae
Pachypanchax sparksorum RDR0562 Andranokaramy Freshwater Aplocheilidae
Pachypanchax sparksorum RDR0561 Andranokaramy Freshwater Aplocheilidae
Pachypanchax sparksorum RDR0559 Andranokaramy Freshwater Aplocheilidae
87
Pachypanchax sparksorum RDR0558 Andranokaramy Freshwater Aplocheilidae
Pachypanchax sparksorum RDR0557 Andranokaramy Freshwater Aplocheilidae
Pachypanchax sparksorum RDR0556 Andranokaramy Freshwater Aplocheilidae
Pachypanchax sparksorum RDR0555 Andranokaramy Freshwater Aplocheilidae
Pachypanchax sparksorum RDR0549 Andranomatsitso Freshwater Aplocheilidae

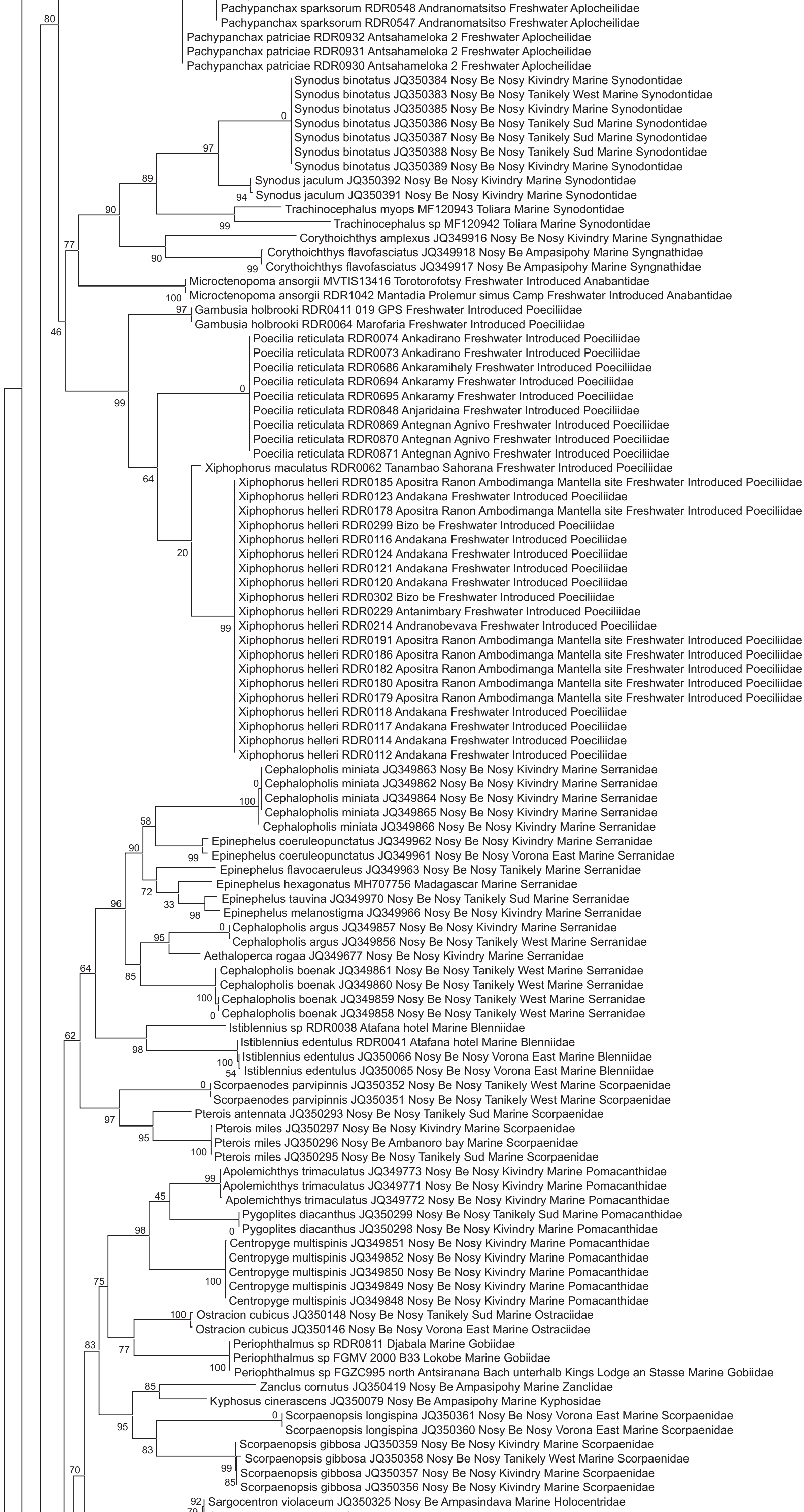

Pachypanchax sparksorum RDR0548 Andranomatsitso Freshwater Aplocheilidae
80
Pachypanchax sparksorum RDR0547 Andranomatsitso Freshwater Aplocheilidae
Pachypanchax patriciae RDR0932 Antsahameloka 2 Freshwater Aplocheilidae
Pachypanchax patriciae RDR0931 Antsahameloka 2 Freshwater Aplocheilidae
Pachypanchax patriciae RDR0930 Antsahameloka 2 Freshwater Aplocheilidae
Synodus binotatus JQ350384 Nosy Be Nosy Kivindry Marine Synodontidae
Synodus binotatus JQ350383 Nosy Be Nosy Tanikely West Marine Synodontidae
Synodus binotatus JQ350385 Nosy Be Nosy Kivindry Marine Synodontidae
0
Synodus binotatus JQ350386 Nosy Be Nosy Tanikely Sud Marine Synodontidae
Synodus binotatus JQ350387 Nosy Be Nosy Tanikely Sud Marine Synodontidae
97
Synodus binotatus JQ350388 Nosy Be Nosy Tanikely Sud Marine Synodontidae
Synodus binotatus JQ350389 Nosy Be Nosy Kivindry Marine Synodontidae
89
Synodus jaculum JQ350392 Nosy Be Nosy Kivindry Marine Synodontidae
94
Synodus jaculum JQ350391 Nosy Be Nosy Kivindry Marine Synodontidae
90
Trachinocephalus myops MF120943 Toliara Marine Synodontidae
99
Trachinocephalus sp MF120942 Toliara Marine Synodontidae
77
Corythoichthys amplexus JQ349916 Nosy Be Nosy Kivindry Marine Syngnathidae
90
Corythoichthys flavofasciatus JQ349918 Nosy Be Ampasipohy Marine Syngnathidae
99
Corythoichthys flavofasciatus JQ349917 Nosy Be Ampasipohy Marine Syngnathidae
Microctenopoma ansorgii MVTIS13416 Torotorofotsy Freshwater Introduced Anabantidae
100
Microctenopoma ansorgii RDR1042 Mantadia Prolemur simus Camp Freshwater Introduced Anabantidae
97
Gambusia holbrooki RDR0411 019 GPS Freshwater Introduced Poeciliidae
46
Gambusia holbrooki RDR0064 Marofaria Freshwater Introduced Poeciliidae
Poecilia reticulata RDR0074 Ankadirano Freshwater Introduced Poeciliidae
Poecilia reticulata RDR0073 Ankadirano Freshwater Introduced Poeciliidae
Poecilia reticulata RDR0686 Ankaramihely Freshwater Introduced Poeciliidae
Poecilia reticulata RDR0694 Ankaramy Freshwater Introduced Poeciliidae
0
Poecilia reticulata RDR0695 Ankaramy Freshwater Introduced Poeciliidae
99
Poecilia reticulata RDR0848 Anjaridaina Freshwater Introduced Poeciliidae
Poecilia reticulata RDR0869 Antegnan Agnivo Freshwater Introduced Poeciliidae
Poecilia reticulata RDR0870 Antegnan Agnivo Freshwater Introduced Poeciliidae
Poecilia reticulata RDR0871 Antegnan Agnivo Freshwater Introduced Poeciliidae
Xiphophorus maculatus RDR0062 Tanambao Sahorana Freshwater Introduced Poeciliidae
64
Xiphophorus helleri RDR0185 Apositra Ranon Ambodimanga Mantella site Freshwater Introduced Poeciliidae
Xiphophorus helleri RDR0123 Andakana Freshwater Introduced Poeciliidae
Xiphophorus helleri RDR0178 Apositra Ranon Ambodimanga Mantella site Freshwater Introduced Poeciliidae
Xiphophorus helleri RDR0299 Bizo be Freshwater Introduced Poeciliidae
Xiphophorus helleri RDR0116 Andakana Freshwater Introduced Poeciliidae
20
Xiphophorus helleri RDR0124 Andakana Freshwater Introduced Poeciliidae
Xiphophorus helleri RDR0121 Andakana Freshwater Introduced Poeciliidae
Xiphophorus helleri RDR0120 Andakana Freshwater Introduced Poeciliidae
Xiphophorus helleri RDR0302 Bizo be Freshwater Introduced Poeciliidae
Xiphophorus helleri RDR0229 Antanimbary Freshwater Introduced Poeciliidae
99
Xiphophorus helleri RDR0214 Andranobevava Freshwater Introduced Poeciliidae
Xiphophorus helleri RDR0191 Apositra Ranon Ambodimanga Mantella site Freshwater Introduced Poeciliidae
Xiphophorus helleri RDR0186 Apositra Ranon Ambodimanga Mantella site Freshwater Introduced Poeciliidae
Xiphophorus helleri RDR0182 Apositra Ranon Ambodimanga Mantella site Freshwater Introduced Poeciliidae
Xiphophorus helleri RDR0180 Apositra Ranon Ambodimanga Mantella site Freshwater Introduced Poeciliidae
Xiphophorus helleri RDR0179 Apositra Ranon Ambodimanga Mantella site Freshwater Introduced Poeciliidae
Xiphophorus helleri RDR0118 Andakana Freshwater Introduced Poeciliidae
Xiphophorus helleri RDR0117 Andakana Freshwater Introduced Poeciliidae
Xiphophorus helleri RDR0114 Andakana Freshwater Introduced Poeciliidae
Xiphophorus helleri RDR0112 Andakana Freshwater Introduced Poeciliidae
Cephalopholis miniata JQ349863 Nosy Be Nosy Kivindry Marine Serranidae
0
Cephalopholis miniata JQ349862 Nosy Be Nosy Kivindry Marine Serranidae
100
Cephalopholis miniata JQ349864 Nosy Be Nosy Kivindry Marine Serranidae
Cephalopholis miniata JQ349865 Nosy Be Nosy Kivindry Marine Serranidae
58
Cephalopholis miniata JQ349866 Nosy Be Nosy Kivindry Marine Serranidae
Epinephelus coeruleopunctatus JQ349962 Nosy Be Nosy Kivindry Marine Serranidae
90
99
Epinephelus coeruleopunctatus JQ349961 Nosy Be Nosy Vorona East Marine Serranidae
Epinephelus flavocaeruleus JQ349963 Nosy Be Nosy Tanikely Marine Serranidae
Epinephelus hexagonatus MH707756 Madagascar Marine Serranidae
72
Epinephelus tauvina JQ349970 Nosy Be Nosy Tanikely Sud Marine Serranidae
96
33
98
Epinephelus melanostigma JQ349966 Nosy Be Nosy Kivindry Marine Serranidae
0
Cephalopholis argus JQ349857 Nosy Be Nosy Kivindry Marine Serranidae
95
Cephalopholis argus JQ349856 Nosy Be Nosy Tanikely West Marine Serranidae
Aethaloperca rogaa JQ349677 Nosy Be Nosy Kivindry Marine Serranidae
64
Cephalopholis boenak JQ349861 Nosy Be Nosy Tanikely West Marine Serranidae
85
Cephalopholis boenak JQ349860 Nosy Be Nosy Tanikely West Marine Serranidae
100
Cephalopholis boenak JQ349859 Nosy Be Nosy Tanikely West Marine Serranidae
0
Cephalopholis boenak JQ349858 Nosy Be Nosy Tanikely West Marine Serranidae
Istiblennius sp RDR0038 Atafana hotel Marine Blenniidae
62
Istiblennius edentulus RDR0041 Atafana hotel Marine Blenniidae
98
Istiblennius edentulus JQ350066 Nosy Be Nosy Vorona East Marine Blenniidae
100
54
Istiblennius edentulus JQ350065 Nosy Be Nosy Vorona East Marine Blenniidae
0
Scorpaenodes parvipinnis JQ350352 Nosy Be Nosy Tanikely West Marine Scorpaenidae
Scorpaenodes parvipinnis JQ350351 Nosy Be Nosy Tanikely West Marine Scorpaenidae
97
Pterois antennata JQ350293 Nosy Be Nosy Tanikely Sud Marine Scorpaenidae
Pterois miles JQ350297 Nosy Be Nosy Kivindry Marine Scorpaenidae
95
Pterois miles JQ350296 Nosy Be Ambanoro bay Marine Scorpaenidae
100
Pterois miles JQ350295 Nosy Be Nosy Tanikely Sud Marine Scorpaenidae
Apolemichthys trimaculatus JQ349773 Nosy Be Nosy Kivindry Marine Pomacanthidae
99
Apolemichthys trimaculatus JQ349771 Nosy Be Nosy Kivindry Marine Pomacanthidae
45
Apolemichthys trimaculatus JQ349772 Nosy Be Nosy Kivindry Marine Pomacanthidae
Pygoplites diacanthus JQ350299 Nosy Be Nosy Tanikely Sud Marine Pomacanthidae
98
0
Pygoplites diacanthus JQ350298 Nosy Be Nosy Kivindry Marine Pomacanthidae
Centropyge multispinis JQ349851 Nosy Be Nosy Kivindry Marine Pomacanthidae
Centropyge multispinis JQ349852 Nosy Be Nosy Kivindry Marine Pomacanthidae
Centropyge multispinis JQ349850 Nosy Be Nosy Kivindry Marine Pomacanthidae
75
100
Centropyge multispinis JQ349849 Nosy Be Nosy Kivindry Marine Pomacanthidae
Centropyge multispinis JQ349848 Nosy Be Nosy Kivindry Marine Pomacanthidae
100
Ostracion cubicus JQ350148 Nosy Be Nosy Tanikely Sud Marine Ostraciidae
Ostracion cubicus JQ350146 Nosy Be Nosy Vorona East Marine Ostraciidae
83
Periophthalmus sp RDR0811 Djabala Marine Gobiidae
77
Periophthalmus sp FGMV 2000 B33 Lokobe Marine Gobiidae
100
Periophthalmus sp FGZC995 north Antsiranana Bach unterhalb Kings Lodge an Stasse Marine Gobiidae
85
Zanclus cornutus JQ350419 Nosy Be Ampasipohy Marine Zanclidae
Kyphosus cinerascens JQ350079 Nosy Be Ampasipohy Marine Kyphosidae
0
Scorpaenopsis longispina JQ350361 Nosy Be Nosy Vorona East Marine Scorpaenidae
95
Scorpaenopsis longispina JQ350360 Nosy Be Nosy Vorona East Marine Scorpaenidae
Scorpaenopsis gibbosa JQ350359 Nosy Be Nosy Kivindry Marine Scorpaenidae
83
Scorpaenopsis gibbosa JQ350358 Nosy Be Nosy Tanikely West Marine Scorpaenidae
70
99
Scorpaenopsis gibbosa JQ350357 Nosy Be Nosy Kivindry Marine Scorpaenidae
85
Scorpaenopsis gibbosa JQ350356 Nosy Be Nosy Kivindry Marine Scorpaenidae
92
Sargocentron violaceum JQ350325 Nosy Be Ampasindava Marine Holocentridae
79

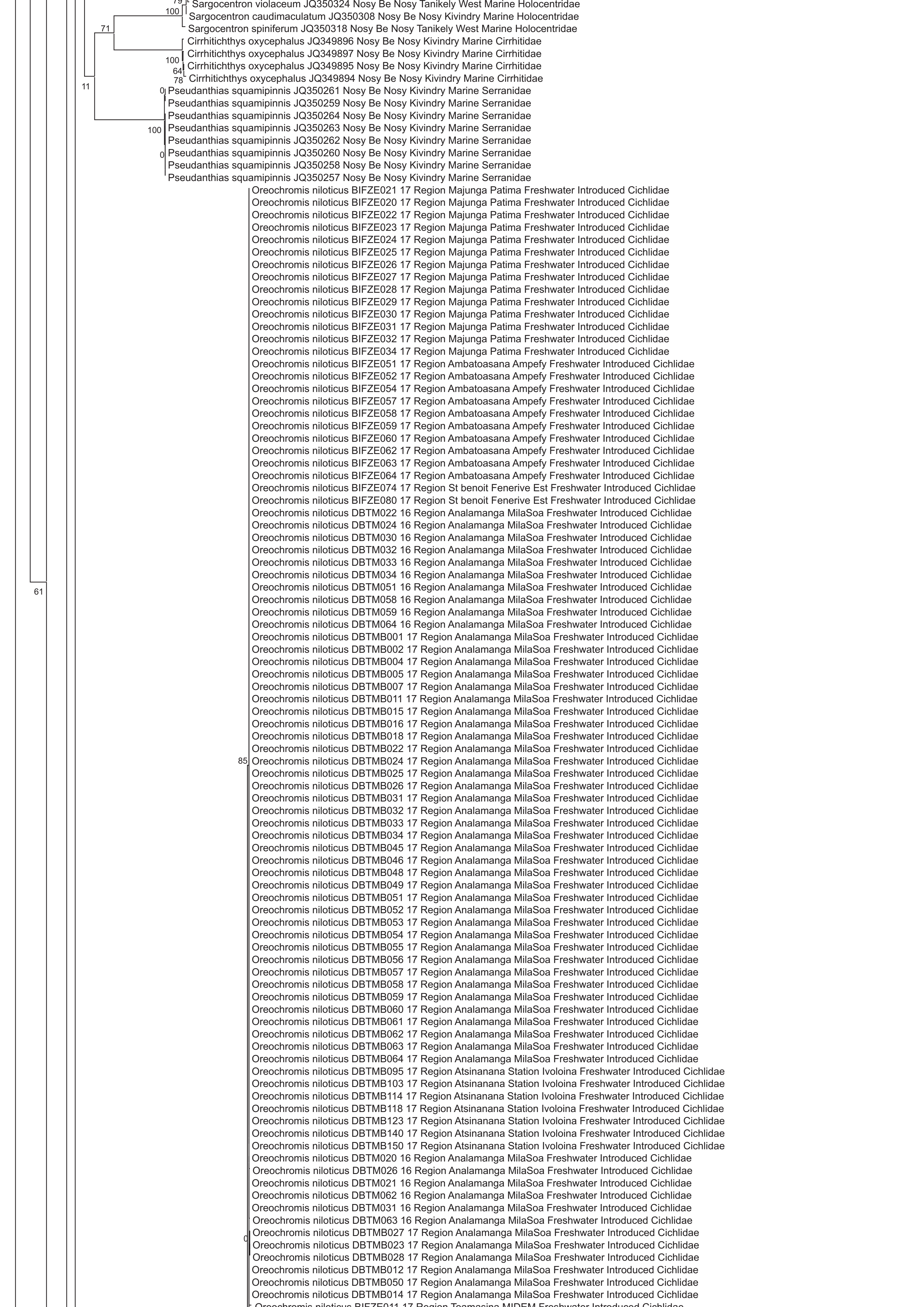

79
Sargocentron violaceum JQ350324 Nosy Be Nosy Tanikely West Marine Holocentridae
100
Sargocentron caudimaculatum JQ350308 Nosy Be Nosy Kivindry Marine Holocentridae
71
Sargocentron spiniferum JQ350318 Nosy Be Nosy Tanikely West Marine Holocentridae
Cirrhitichthys oxycephalus JQ349896 Nosy Be Nosy Kivindry Marine Cirrhitidae
100
Cirrhitichthys oxycephalus JQ349897 Nosy Be Nosy Kivindry Marine Cirrhitidae
64
Cirrhitichthys oxycephalus JQ349895 Nosy Be Nosy Kivindry Marine Cirrhitidae
78
Cirrhitichthys oxycephalus JQ349894 Nosy Be Nosy Kivindry Marine Cirrhitidae
11
0
Pseudanthias squamipinnis JQ350261 Nosy Be Nosy Kivindry Marine Serranidae
Pseudanthias squamipinnis JQ350259 Nosy Be Nosy Kivindry Marine Serranidae
Pseudanthias squamipinnis JQ350264 Nosy Be Nosy Kivindry Marine Serranidae
100
Pseudanthias squamipinnis JQ350263 Nosy Be Nosy Kivindry Marine Serranidae
Pseudanthias squamipinnis JQ350262 Nosy Be Nosy Kivindry Marine Serranidae
0
Pseudanthias squamipinnis JQ350260 Nosy Be Nosy Kivindry Marine Serranidae
Pseudanthias squamipinnis JQ350258 Nosy Be Nosy Kivindry Marine Serranidae
Pseudanthias squamipinnis JQ350257 Nosy Be Nosy Kivindry Marine Serranidae
Oreochromis niloticus BIFZE021 17 Region Majunga Patima Freshwater Introduced Cichlidae
Oreochromis niloticus BIFZE020 17 Region Majunga Patima Freshwater Introduced Cichlidae
Oreochromis niloticus BIFZE022 17 Region Majunga Patima Freshwater Introduced Cichlidae
Oreochromis niloticus BIFZE023 17 Region Majunga Patima Freshwater Introduced Cichlidae
Oreochromis niloticus BIFZE024 17 Region Majunga Patima Freshwater Introduced Cichlidae
Oreochromis niloticus BIFZE025 17 Region Majunga Patima Freshwater Introduced Cichlidae
Oreochromis niloticus BIFZE026 17 Region Majunga Patima Freshwater Introduced Cichlidae
Oreochromis niloticus BIFZE027 17 Region Majunga Patima Freshwater Introduced Cichlidae
Oreochromis niloticus BIFZE028 17 Region Majunga Patima Freshwater Introduced Cichlidae
Oreochromis niloticus BIFZE029 17 Region Majunga Patima Freshwater Introduced Cichlidae
Oreochromis niloticus BIFZE030 17 Region Majunga Patima Freshwater Introduced Cichlidae
Oreochromis niloticus BIFZE031 17 Region Majunga Patima Freshwater Introduced Cichlidae
Oreochromis niloticus BIFZE032 17 Region Majunga Patima Freshwater Introduced Cichlidae
Oreochromis niloticus BIFZE034 17 Region Majunga Patima Freshwater Introduced Cichlidae
Oreochromis niloticus BIFZE051 17 Region Ambatoasana Ampefy Freshwater Introduced Cichlidae
Oreochromis niloticus BIFZE052 17 Region Ambatoasana Ampefy Freshwater Introduced Cichlidae
Oreochromis niloticus BIFZE054 17 Region Ambatoasana Ampefy Freshwater Introduced Cichlidae
Oreochromis niloticus BIFZE057 17 Region Ambatoasana Ampefy Freshwater Introduced Cichlidae
Oreochromis niloticus BIFZE058 17 Region Ambatoasana Ampefy Freshwater Introduced Cichlidae
Oreochromis niloticus BIFZE059 17 Region Ambatoasana Ampefy Freshwater Introduced Cichlidae
Oreochromis niloticus BIFZE060 17 Region Ambatoasana Ampefy Freshwater Introduced Cichlidae
Oreochromis niloticus BIFZE062 17 Region Ambatoasana Ampefy Freshwater Introduced Cichlidae
Oreochromis niloticus BIFZE063 17 Region Ambatoasana Ampefy Freshwater Introduced Cichlidae
Oreochromis niloticus BIFZE064 17 Region Ambatoasana Ampefy Freshwater Introduced Cichlidae
Oreochromis niloticus BIFZE074 17 Region St benoit Fenerive Est Freshwater Introduced Cichlidae
Oreochromis niloticus BIFZE080 17 Region St benoit Fenerive Est Freshwater Introduced Cichlidae
Oreochromis niloticus DBTM022 16 Region Analamanga MilaSoa Freshwater Introduced Cichlidae
Oreochromis niloticus DBTM024 16 Region Analamanga MilaSoa Freshwater Introduced Cichlidae
Oreochromis niloticus DBTM030 16 Region Analamanga MilaSoa Freshwater Introduced Cichlidae
Oreochromis niloticus DBTM032 16 Region Analamanga MilaSoa Freshwater Introduced Cichlidae
Oreochromis niloticus DBTM033 16 Region Analamanga MilaSoa Freshwater Introduced Cichlidae
Oreochromis niloticus DBTM034 16 Region Analamanga MilaSoa Freshwater Introduced Cichlidae
61
Oreochromis niloticus DBTM051 16 Region Analamanga MilaSoa Freshwater Introduced Cichlidae
Oreochromis niloticus DBTM058 16 Region Analamanga MilaSoa Freshwater Introduced Cichlidae
Oreochromis niloticus DBTM059 16 Region Analamanga MilaSoa Freshwater Introduced Cichlidae
Oreochromis niloticus DBTM064 16 Region Analamanga MilaSoa Freshwater Introduced Cichlidae
Oreochromis niloticus DBTMB001 17 Region Analamanga MilaSoa Freshwater Introduced Cichlidae
Oreochromis niloticus DBTMB002 17 Region Analamanga MilaSoa Freshwater Introduced Cichlidae
Oreochromis niloticus DBTMB004 17 Region Analamanga MilaSoa Freshwater Introduced Cichlidae
Oreochromis niloticus DBTMB005 17 Region Analamanga MilaSoa Freshwater Introduced Cichlidae
Oreochromis niloticus DBTMB007 17 Region Analamanga MilaSoa Freshwater Introduced Cichlidae
Oreochromis niloticus DBTMB011 17 Region Analamanga MilaSoa Freshwater Introduced Cichlidae
Oreochromis niloticus DBTMB015 17 Region Analamanga MilaSoa Freshwater Introduced Cichlidae
Oreochromis niloticus DBTMB016 17 Region Analamanga MilaSoa Freshwater Introduced Cichlidae
Oreochromis niloticus DBTMB018 17 Region Analamanga MilaSoa Freshwater Introduced Cichlidae
Oreochromis niloticus DBTMB022 17 Region Analamanga MilaSoa Freshwater Introduced Cichlidae
85
Oreochromis niloticus DBTMB024 17 Region Analamanga MilaSoa Freshwater Introduced Cichlidae
Oreochromis niloticus DBTMB025 17 Region Analamanga MilaSoa Freshwater Introduced Cichlidae
Oreochromis niloticus DBTMB026 17 Region Analamanga MilaSoa Freshwater Introduced Cichlidae
Oreochromis niloticus DBTMB031 17 Region Analamanga MilaSoa Freshwater Introduced Cichlidae
Oreochromis niloticus DBTMB032 17 Region Analamanga MilaSoa Freshwater Introduced Cichlidae
Oreochromis niloticus DBTMB033 17 Region Analamanga MilaSoa Freshwater Introduced Cichlidae
Oreochromis niloticus DBTMB034 17 Region Analamanga MilaSoa Freshwater Introduced Cichlidae
Oreochromis niloticus DBTMB045 17 Region Analamanga MilaSoa Freshwater Introduced Cichlidae
Oreochromis niloticus DBTMB046 17 Region Analamanga MilaSoa Freshwater Introduced Cichlidae
Oreochromis niloticus DBTMB048 17 Region Analamanga MilaSoa Freshwater Introduced Cichlidae
Oreochromis niloticus DBTMB049 17 Region Analamanga MilaSoa Freshwater Introduced Cichlidae
Oreochromis niloticus DBTMB051 17 Region Analamanga MilaSoa Freshwater Introduced Cichlidae
Oreochromis niloticus DBTMB052 17 Region Analamanga MilaSoa Freshwater Introduced Cichlidae
Oreochromis niloticus DBTMB053 17 Region Analamanga MilaSoa Freshwater Introduced Cichlidae
Oreochromis niloticus DBTMB054 17 Region Analamanga MilaSoa Freshwater Introduced Cichlidae
Oreochromis niloticus DBTMB055 17 Region Analamanga MilaSoa Freshwater Introduced Cichlidae
Oreochromis niloticus DBTMB056 17 Region Analamanga MilaSoa Freshwater Introduced Cichlidae
Oreochromis niloticus DBTMB057 17 Region Analamanga MilaSoa Freshwater Introduced Cichlidae
Oreochromis niloticus DBTMB058 17 Region Analamanga MilaSoa Freshwater Introduced Cichlidae
Oreochromis niloticus DBTMB059 17 Region Analamanga MilaSoa Freshwater Introduced Cichlidae
Oreochromis niloticus DBTMB060 17 Region Analamanga MilaSoa Freshwater Introduced Cichlidae
Oreochromis niloticus DBTMB061 17 Region Analamanga MilaSoa Freshwater Introduced Cichlidae
Oreochromis niloticus DBTMB062 17 Region Analamanga MilaSoa Freshwater Introduced Cichlidae
Oreochromis niloticus DBTMB063 17 Region Analamanga MilaSoa Freshwater Introduced Cichlidae
Oreochromis niloticus DBTMB064 17 Region Analamanga MilaSoa Freshwater Introduced Cichlidae
Oreochromis niloticus DBTMB095 17 Region Atsinanana Station Ivoloina Freshwater Introduced Cichlidae
Oreochromis niloticus DBTMB103 17 Region Atsinanana Station Ivoloina Freshwater Introduced Cichlidae
Oreochromis niloticus DBTMB114 17 Region Atsinanana Station Ivoloina Freshwater Introduced Cichlidae
Oreochromis niloticus DBTMB118 17 Region Atsinanana Station Ivoloina Freshwater Introduced Cichlidae
Oreochromis niloticus DBTMB123 17 Region Atsinanana Station Ivoloina Freshwater Introduced Cichlidae
Oreochromis niloticus DBTMB140 17 Region Atsinanana Station Ivoloina Freshwater Introduced Cichlidae
Oreochromis niloticus DBTMB150 17 Region Atsinanana Station Ivoloina Freshwater Introduced Cichlidae
Oreochromis niloticus DBTM020 16 Region Analamanga MilaSoa Freshwater Introduced Cichlidae
Oreochromis niloticus DBTM026 16 Region Analamanga MilaSoa Freshwater Introduced Cichlidae
Oreochromis niloticus DBTM021 16 Region Analamanga MilaSoa Freshwater Introduced Cichlidae
Oreochromis niloticus DBTM062 16 Region Analamanga MilaSoa Freshwater Introduced Cichlidae
Oreochromis niloticus DBTM031 16 Region Analamanga MilaSoa Freshwater Introduced Cichlidae
Oreochromis niloticus DBTM063 16 Region Analamanga MilaSoa Freshwater Introduced Cichlidae
Oreochromis niloticus DBTMB027 17 Region Analamanga MilaSoa Freshwater Introduced Cichlidae
0
Oreochromis niloticus DBTMB023 17 Region Analamanga MilaSoa Freshwater Introduced Cichlidae
Oreochromis niloticus DBTMB028 17 Region Analamanga MilaSoa Freshwater Introduced Cichlidae
Oreochromis niloticus DBTMB012 17 Region Analamanga MilaSoa Freshwater Introduced Cichlidae
Oreochromis niloticus DBTMB050 17 Region Analamanga MilaSoa Freshwater Introduced Cichlidae
Oreochromis niloticus DBTMB014 17 Region Analamanga MilaSoa Freshwater Introduced Cichlidae

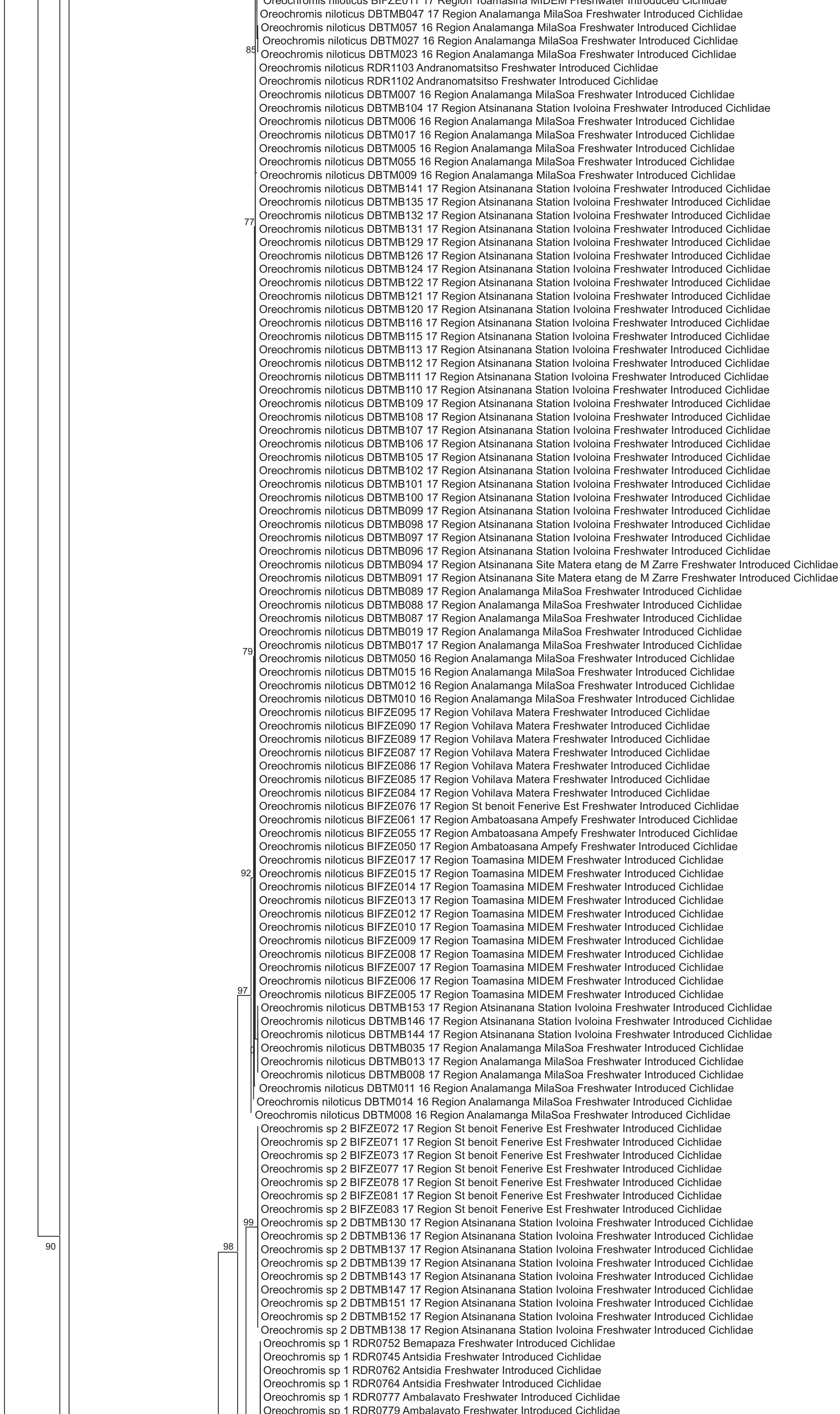
Oreochromis niloticus BIFZE011 17 Region Toamasina MIDEM Freshwater Introduced Cichlidae
Oreochromis niloticus DBTMB047 17 Region Analamanga MilaSoa Freshwater Introduced Cichlidae
Oreochromis niloticus DBTM057 16 Region Analamanga MilaSoa Freshwater Introduced Cichlidae
Oreochromis niloticus DBTM027 16 Region Analamanga MilaSoa Freshwater Introduced Cichlidae
85
Oreochromis niloticus DBTM023 16 Region Analamanga MilaSoa Freshwater Introduced Cichlidae
Oreochromis niloticus RDR1103 Andranomatsitso Freshwater Introduced Cichlidae
Oreochromis niloticus RDR1102 Andranomatsitso Freshwater Introduced Cichlidae
Oreochromis niloticus DBTM007 16 Region Analamanga MilaSoa Freshwater Introduced Cichlidae
Oreochromis niloticus DBTMB104 17 Region Atsinanana Station Ivoloina Freshwater Introduced Cichlidae
Oreochromis niloticus DBTM006 16 Region Analamanga MilaSoa Freshwater Introduced Cichlidae
Oreochromis niloticus DBTM017 16 Region Analamanga MilaSoa Freshwater Introduced Cichlidae
Oreochromis niloticus DBTM005 16 Region Analamanga MilaSoa Freshwater Introduced Cichlidae
Oreochromis niloticus DBTM055 16 Region Analamanga MilaSoa Freshwater Introduced Cichlidae
Oreochromis niloticus DBTM009 16 Region Analamanga MilaSoa Freshwater Introduced Cichlidae
Oreochromis niloticus DBTMB141 17 Region Atsinanana Station Ivoloina Freshwater Introduced Cichlidae
Oreochromis niloticus DBTMB135 17 Region Atsinanana Station Ivoloina Freshwater Introduced Cichlidae
Oreochromis niloticus DBTMB132 17 Region Atsinanana Station Ivoloina Freshwater Introduced Cichlidae
77
Oreochromis niloticus DBTMB131 17 Region Atsinanana Station Ivoloina Freshwater Introduced Cichlidae
Oreochromis niloticus DBTMB129 17 Region Atsinanana Station Ivoloina Freshwater Introduced Cichlidae
Oreochromis niloticus DBTMB126 17 Region Atsinanana Station Ivoloina Freshwater Introduced Cichlidae
Oreochromis niloticus DBTMB124 17 Region Atsinanana Station Ivoloina Freshwater Introduced Cichlidae
Oreochromis niloticus DBTMB122 17 Region Atsinanana Station Ivoloina Freshwater Introduced Cichlidae
Oreochromis niloticus DBTMB121 17 Region Atsinanana Station Ivoloina Freshwater Introduced Cichlidae
Oreochromis niloticus DBTMB120 17 Region Atsinanana Station Ivoloina Freshwater Introduced Cichlidae
Oreochromis niloticus DBTMB116 17 Region Atsinanana Station Ivoloina Freshwater Introduced Cichlidae
Oreochromis niloticus DBTMB115 17 Region Atsinanana Station Ivoloina Freshwater Introduced Cichlidae
Oreochromis niloticus DBTMB113 17 Region Atsinanana Station Ivoloina Freshwater Introduced Cichlidae
Oreochromis niloticus DBTMB112 17 Region Atsinanana Station Ivoloina Freshwater Introduced Cichlidae
Oreochromis niloticus DBTMB111 17 Region Atsinanana Station Ivoloina Freshwater Introduced Cichlidae
Oreochromis niloticus DBTMB110 17 Region Atsinanana Station Ivoloina Freshwater Introduced Cichlidae
Oreochromis niloticus DBTMB109 17 Region Atsinanana Station Ivoloina Freshwater Introduced Cichlidae
Oreochromis niloticus DBTMB108 17 Region Atsinanana Station Ivoloina Freshwater Introduced Cichlidae
Oreochromis niloticus DBTMB107 17 Region Atsinanana Station Ivoloina Freshwater Introduced Cichlidae
Oreochromis niloticus DBTMB106 17 Region Atsinanana Station Ivoloina Freshwater Introduced Cichlidae
Oreochromis niloticus DBTMB105 17 Region Atsinanana Station Ivoloina Freshwater Introduced Cichlidae
Oreochromis niloticus DBTMB102 17 Region Atsinanana Station Ivoloina Freshwater Introduced Cichlidae
Oreochromis niloticus DBTMB101 17 Region Atsinanana Station Ivoloina Freshwater Introduced Cichlidae
Oreochromis niloticus DBTMB100 17 Region Atsinanana Station Ivoloina Freshwater Introduced Cichlidae
Oreochromis niloticus DBTMB099 17 Region Atsinanana Station Ivoloina Freshwater Introduced Cichlidae
Oreochromis niloticus DBTMB098 17 Region Atsinanana Station Ivoloina Freshwater Introduced Cichlidae
Oreochromis niloticus DBTMB097 17 Region Atsinanana Station Ivoloina Freshwater Introduced Cichlidae
Oreochromis niloticus DBTMB096 17 Region Atsinanana Station Ivoloina Freshwater Introduced Cichlidae
Oreochromis niloticus DBTMB094 17 Region Atsinanana Site Matera etang de M Zarre Freshwater Introduced Cichlidae
Oreochromis niloticus DBTMB091 17 Region Atsinanana Site Matera etang de M Zarre Freshwater Introduced Cichlidae
Oreochromis niloticus DBTMB089 17 Region Analamanga MilaSoa Freshwater Introduced Cichlidae
Oreochromis niloticus DBTMB088 17 Region Analamanga MilaSoa Freshwater Introduced Cichlidae
Oreochromis niloticus DBTMB087 17 Region Analamanga MilaSoa Freshwater Introduced Cichlidae
Oreochromis niloticus DBTMB019 17 Region Analamanga MilaSoa Freshwater Introduced Cichlidae
Oreochromis niloticus DBTMB017 17 Region Analamanga MilaSoa Freshwater Introduced Cichlidae
79
Oreochromis niloticus DBTM050 16 Region Analamanga MilaSoa Freshwater Introduced Cichlidae
Oreochromis niloticus DBTM015 16 Region Analamanga MilaSoa Freshwater Introduced Cichlidae
Oreochromis niloticus DBTM012 16 Region Analamanga MilaSoa Freshwater Introduced Cichlidae
Oreochromis niloticus DBTM010 16 Region Analamanga MilaSoa Freshwater Introduced Cichlidae
Oreochromis niloticus BIFZE095 17 Region Vohilava Matera Freshwater Introduced Cichlidae
Oreochromis niloticus BIFZE090 17 Region Vohilava Matera Freshwater Introduced Cichlidae
Oreochromis niloticus BIFZE089 17 Region Vohilava Matera Freshwater Introduced Cichlidae
Oreochromis niloticus BIFZE087 17 Region Vohilava Matera Freshwater Introduced Cichlidae
Oreochromis niloticus BIFZE086 17 Region Vohilava Matera Freshwater Introduced Cichlidae
Oreochromis niloticus BIFZE085 17 Region Vohilava Matera Freshwater Introduced Cichlidae
Oreochromis niloticus BIFZE084 17 Region Vohilava Matera Freshwater Introduced Cichlidae
Oreochromis niloticus BIFZE076 17 Region St benoit Fenerive Est Freshwater Introduced Cichlidae
Oreochromis niloticus BIFZE061 17 Region Ambatoasana Ampefy Freshwater Introduced Cichlidae
Oreochromis niloticus BIFZE055 17 Region Ambatoasana Ampefy Freshwater Introduced Cichlidae
Oreochromis niloticus BIFZE050 17 Region Ambatoasana Ampefy Freshwater Introduced Cichlidae
Oreochromis niloticus BIFZE017 17 Region Toamasina MIDEM Freshwater Introduced Cichlidae
92
Oreochromis niloticus BIFZE015 17 Region Toamasina MIDEM Freshwater Introduced Cichlidae
Oreochromis niloticus BIFZE014 17 Region Toamasina MIDEM Freshwater Introduced Cichlidae
Oreochromis niloticus BIFZE013 17 Region Toamasina MIDEM Freshwater Introduced Cichlidae
Oreochromis niloticus BIFZE012 17 Region Toamasina MIDEM Freshwater Introduced Cichlidae
Oreochromis niloticus BIFZE010 17 Region Toamasina MIDEM Freshwater Introduced Cichlidae
Oreochromis niloticus BIFZE009 17 Region Toamasina MIDEM Freshwater Introduced Cichlidae
Oreochromis niloticus BIFZE008 17 Region Toamasina MIDEM Freshwater Introduced Cichlidae
Oreochromis niloticus BIFZE007 17 Region Toamasina MIDEM Freshwater Introduced Cichlidae
Oreochromis niloticus BIFZE006 17 Region Toamasina MIDEM Freshwater Introduced Cichlidae
97
Oreochromis niloticus BIFZE005 17 Region Toamasina MIDEM Freshwater Introduced Cichlidae
Oreochromis niloticus DBTMB153 17 Region Atsinanana Station Ivoloina Freshwater Introduced Cichlidae
Oreochromis niloticus DBTMB146 17 Region Atsinanana Station Ivoloina Freshwater Introduced Cichlidae
Oreochromis niloticus DBTMB144 17 Region Atsinanana Station Ivoloina Freshwater Introduced Cichlidae
Oreochromis niloticus DBTMB035 17 Region Analamanga MilaSoa Freshwater Introduced Cichlidae
Oreochromis niloticus DBTMB013 17 Region Analamanga MilaSoa Freshwater Introduced Cichlidae
Oreochromis niloticus DBTMB008 17 Region Analamanga MilaSoa Freshwater Introduced Cichlidae
Oreochromis niloticus DBTM011 16 Region Analamanga MilaSoa Freshwater Introduced Cichlidae
Oreochromis niloticus DBTM014 16 Region Analamanga MilaSoa Freshwater Introduced Cichlidae
Oreochromis niloticus DBTM008 16 Region Analamanga MilaSoa Freshwater Introduced Cichlidae
Oreochromis sp 2 BIFZE072 17 Region St benoit Fenerive Est Freshwater Introduced Cichlidae
Oreochromis sp 2 BIFZE071 17 Region St benoit Fenerive Est Freshwater Introduced Cichlidae
Oreochromis sp 2 BIFZE073 17 Region St benoit Fenerive Est Freshwater Introduced Cichlidae
Oreochromis sp 2 BIFZE077 17 Region St benoit Fenerive Est Freshwater Introduced Cichlidae
Oreochromis sp 2 BIFZE078 17 Region St benoit Fenerive Est Freshwater Introduced Cichlidae
Oreochromis sp 2 BIFZE081 17 Region St benoit Fenerive Est Freshwater Introduced Cichlidae
Oreochromis sp 2 BIFZE083 17 Region St benoit Fenerive Est Freshwater Introduced Cichlidae
99
Oreochromis sp 2 DBTMB130 17 Region Atsinanana Station Ivoloina Freshwater Introduced Cichlidae
Oreochromis sp 2 DBTMB136 17 Region Atsinanana Station Ivoloina Freshwater Introduced Cichlidae
90
98
Oreochromis sp 2 DBTMB137 17 Region Atsinanana Station Ivoloina Freshwater Introduced Cichlidae
Oreochromis sp 2 DBTMB139 17 Region Atsinanana Station Ivoloina Freshwater Introduced Cichlidae
Oreochromis sp 2 DBTMB143 17 Region Atsinanana Station Ivoloina Freshwater Introduced Cichlidae
Oreochromis sp 2 DBTMB147 17 Region Atsinanana Station Ivoloina Freshwater Introduced Cichlidae
Oreochromis sp 2 DBTMB151 17 Region Atsinanana Station Ivoloina Freshwater Introduced Cichlidae
Oreochromis sp 2 DBTMB152 17 Region Atsinanana Station Ivoloina Freshwater Introduced Cichlidae
Oreochromis sp 2 DBTMB138 17 Region Atsinanana Station Ivoloina Freshwater Introduced Cichlidae
Oreochromis sp 1 RDR0752 Bemapaza Freshwater Introduced Cichlidae
Oreochromis sp 1 RDR0745 Antsidia Freshwater Introduced Cichlidae
Oreochromis sp 1 RDR0762 Antsidia Freshwater Introduced Cichlidae
Oreochromis sp 1 RDR0764 Antsidia Freshwater Introduced Cichlidae
Oreochromis sp 1 RDR0777 Ambalavato Freshwater Introduced Cichlidae
Oreochromis sp 1 RDR0779 Ambalavato Freshwater Introduced Cichlidae

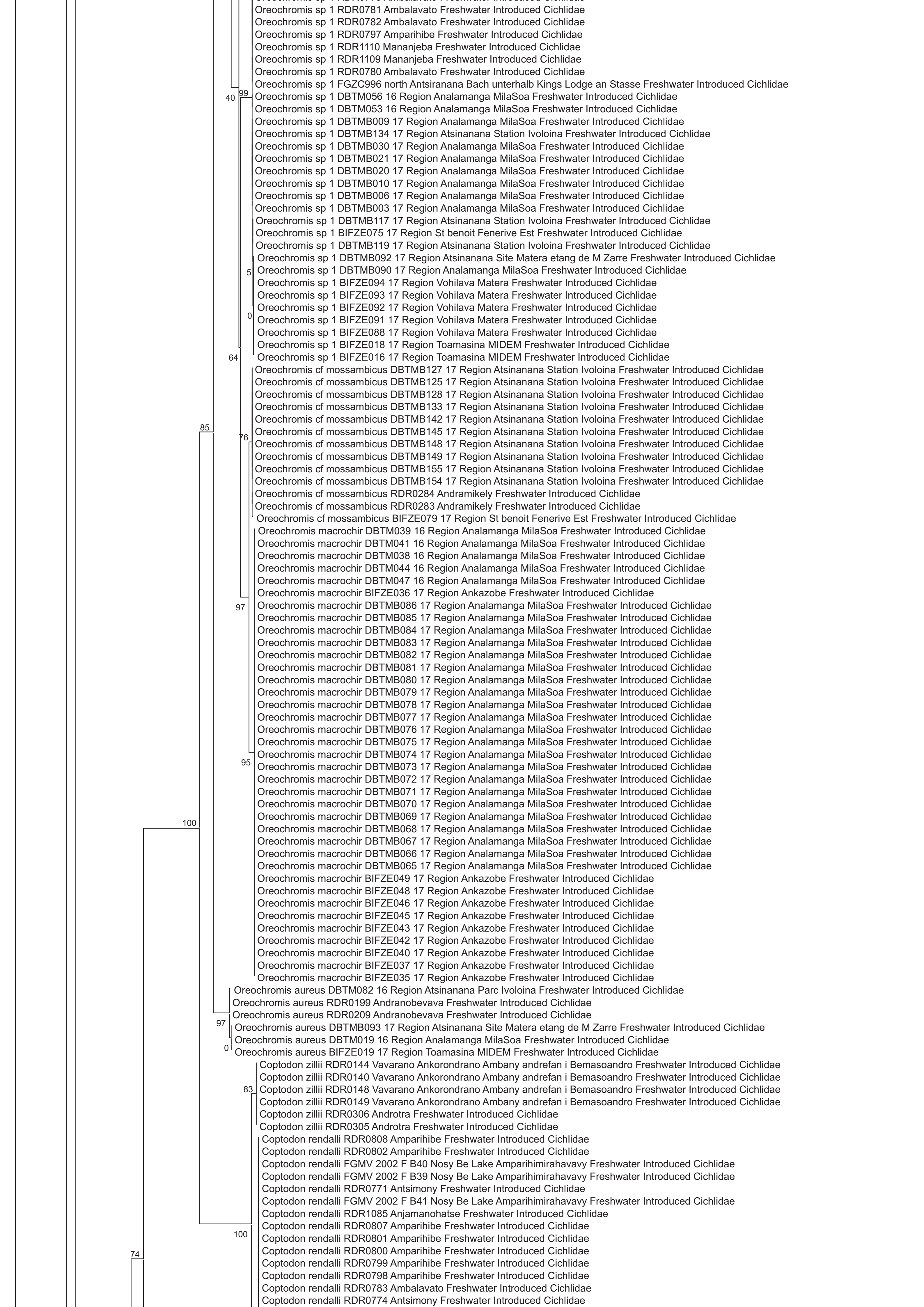
Oreochromis sp 1 RDR0781 Ambalavato Freshwater Introduced Cichlidae
Oreochromis sp 1 RDR0782 Ambalavato Freshwater Introduced Cichlidae
Oreochromis sp 1 RDR0797 Amparihibe Freshwater Introduced Cichlidae
Oreochromis sp 1 RDR1110 Mananjeba Freshwater Introduced Cichlidae
Oreochromis sp 1 RDR1109 Mananjeba Freshwater Introduced Cichlidae
Oreochromis sp 1 RDR0780 Ambalavato Freshwater Introduced Cichlidae
Oreochromis sp 1 FGZC996 north Antsiranana Bach unterhalb Kings Lodge an Stasse Freshwater Introduced Cichlidae
99
40
Oreochromis sp 1 DBTM056 16 Region Analamanga MilaSoa Freshwater Introduced Cichlidae
Oreochromis sp 1 DBTM053 16 Region Analamanga MilaSoa Freshwater Introduced Cichlidae
Oreochromis sp 1 DBTMB009 17 Region Analamanga MilaSoa Freshwater Introduced Cichlidae
Oreochromis sp 1 DBTMB134 17 Region Atsinanana Station Ivoloina Freshwater Introduced Cichlidae
Oreochromis sp 1 DBTMB030 17 Region Analamanga MilaSoa Freshwater Introduced Cichlidae
Oreochromis sp 1 DBTMB021 17 Region Analamanga MilaSoa Freshwater Introduced Cichlidae
Oreochromis sp 1 DBTMB020 17 Region Analamanga MilaSoa Freshwater Introduced Cichlidae
Oreochromis sp 1 DBTMB010 17 Region Analamanga MilaSoa Freshwater Introduced Cichlidae
Oreochromis sp 1 DBTMB006 17 Region Analamanga MilaSoa Freshwater Introduced Cichlidae
Oreochromis sp 1 DBTMB003 17 Region Analamanga MilaSoa Freshwater Introduced Cichlidae
Oreochromis sp 1 DBTMB117 17 Region Atsinanana Station Ivoloina Freshwater Introduced Cichlidae
Oreochromis sp 1 BIFZE075 17 Region St benoit Fenerive Est Freshwater Introduced Cichlidae
Oreochromis sp 1 DBTMB119 17 Region Atsinanana Station Ivoloina Freshwater Introduced Cichlidae
Oreochromis sp 1 DBTMB092 17 Region Atsinanana Site Matera etang de M Zarre Freshwater Introduced Cichlidae
5
Oreochromis sp 1 DBTMB090 17 Region Analamanga MilaSoa Freshwater Introduced Cichlidae
Oreochromis sp 1 BIFZE094 17 Region Vohilava Matera Freshwater Introduced Cichlidae
Oreochromis sp 1 BIFZE093 17 Region Vohilava Matera Freshwater Introduced Cichlidae
Oreochromis sp 1 BIFZE092 17 Region Vohilava Matera Freshwater Introduced Cichlidae
0
Oreochromis sp 1 BIFZE091 17 Region Vohilava Matera Freshwater Introduced Cichlidae
Oreochromis sp 1 BIFZE088 17 Region Vohilava Matera Freshwater Introduced Cichlidae
Oreochromis sp 1 BIFZE018 17 Region Toamasina MIDEM Freshwater Introduced Cichlidae
64
Oreochromis sp 1 BIFZE016 17 Region Toamasina MIDEM Freshwater Introduced Cichlidae
Oreochromis cf mossambicus DBTMB127 17 Region Atsinanana Station Ivoloina Freshwater Introduced Cichlidae
Oreochromis cf mossambicus DBTMB125 17 Region Atsinanana Station Ivoloina Freshwater Introduced Cichlidae
Oreochromis cf mossambicus DBTMB128 17 Region Atsinanana Station Ivoloina Freshwater Introduced Cichlidae
Oreochromis cf mossambicus DBTMB133 17 Region Atsinanana Station Ivoloina Freshwater Introduced Cichlidae
Oreochromis cf mossambicus DBTMB142 17 Region Atsinanana Station Ivoloina Freshwater Introduced Cichlidae
85
Oreochromis cf mossambicus DBTMB145 17 Region Atsinanana Station Ivoloina Freshwater Introduced Cichlidae
76
Oreochromis cf mossambicus DBTMB148 17 Region Atsinanana Station Ivoloina Freshwater Introduced Cichlidae
Oreochromis cf mossambicus DBTMB149 17 Region Atsinanana Station Ivoloina Freshwater Introduced Cichlidae
Oreochromis cf mossambicus DBTMB155 17 Region Atsinanana Station Ivoloina Freshwater Introduced Cichlidae
Oreochromis cf mossambicus DBTMB154 17 Region Atsinanana Station Ivoloina Freshwater Introduced Cichlidae
Oreochromis cf mossambicus RDR0284 Andramikely Freshwater Introduced Cichlidae
Oreochromis cf mossambicus RDR0283 Andramikely Freshwater Introduced Cichlidae
Oreochromis cf mossambicus BIFZE079 17 Region St benoit Fenerive Est Freshwater Introduced Cichlidae
Oreochromis macrochir DBTM039 16 Region Analamanga MilaSoa Freshwater Introduced Cichlidae
Oreochromis macrochir DBTM041 16 Region Analamanga MilaSoa Freshwater Introduced Cichlidae
Oreochromis macrochir DBTM038 16 Region Analamanga MilaSoa Freshwater Introduced Cichlidae
Oreochromis macrochir DBTM044 16 Region Analamanga MilaSoa Freshwater Introduced Cichlidae
Oreochromis macrochir DBTM047 16 Region Analamanga MilaSoa Freshwater Introduced Cichlidae
Oreochromis macrochir BIFZE036 17 Region Ankazobe Freshwater Introduced Cichlidae
97
Oreochromis macrochir DBTMB086 17 Region Analamanga MilaSoa Freshwater Introduced Cichlidae
Oreochromis macrochir DBTMB085 17 Region Analamanga MilaSoa Freshwater Introduced Cichlidae
Oreochromis macrochir DBTMB084 17 Region Analamanga MilaSoa Freshwater Introduced Cichlidae
Oreochromis macrochir DBTMB083 17 Region Analamanga MilaSoa Freshwater Introduced Cichlidae
Oreochromis macrochir DBTMB082 17 Region Analamanga MilaSoa Freshwater Introduced Cichlidae
Oreochromis macrochir DBTMB081 17 Region Analamanga MilaSoa Freshwater Introduced Cichlidae
Oreochromis macrochir DBTMB080 17 Region Analamanga MilaSoa Freshwater Introduced Cichlidae
Oreochromis macrochir DBTMB079 17 Region Analamanga MilaSoa Freshwater Introduced Cichlidae
Oreochromis macrochir DBTMB078 17 Region Analamanga MilaSoa Freshwater Introduced Cichlidae
Oreochromis macrochir DBTMB077 17 Region Analamanga MilaSoa Freshwater Introduced Cichlidae
Oreochromis macrochir DBTMB076 17 Region Analamanga MilaSoa Freshwater Introduced Cichlidae
Oreochromis macrochir DBTMB075 17 Region Analamanga MilaSoa Freshwater Introduced Cichlidae
Oreochromis macrochir DBTMB074 17 Region Analamanga MilaSoa Freshwater Introduced Cichlidae
95
Oreochromis macrochir DBTMB073 17 Region Analamanga MilaSoa Freshwater Introduced Cichlidae
Oreochromis macrochir DBTMB072 17 Region Analamanga MilaSoa Freshwater Introduced Cichlidae
Oreochromis macrochir DBTMB071 17 Region Analamanga MilaSoa Freshwater Introduced Cichlidae
Oreochromis macrochir DBTMB070 17 Region Analamanga MilaSoa Freshwater Introduced Cichlidae
Oreochromis macrochir DBTMB069 17 Region Analamanga MilaSoa Freshwater Introduced Cichlidae
100
Oreochromis macrochir DBTMB068 17 Region Analamanga MilaSoa Freshwater Introduced Cichlidae
Oreochromis macrochir DBTMB067 17 Region Analamanga MilaSoa Freshwater Introduced Cichlidae
Oreochromis macrochir DBTMB066 17 Region Analamanga MilaSoa Freshwater Introduced Cichlidae
Oreochromis macrochir DBTMB065 17 Region Analamanga MilaSoa Freshwater Introduced Cichlidae
Oreochromis macrochir BIFZE049 17 Region Ankazobe Freshwater Introduced Cichlidae
Oreochromis macrochir BIFZE048 17 Region Ankazobe Freshwater Introduced Cichlidae
Oreochromis macrochir BIFZE046 17 Region Ankazobe Freshwater Introduced Cichlidae
Oreochromis macrochir BIFZE045 17 Region Ankazobe Freshwater Introduced Cichlidae
Oreochromis macrochir BIFZE043 17 Region Ankazobe Freshwater Introduced Cichlidae
Oreochromis macrochir BIFZE042 17 Region Ankazobe Freshwater Introduced Cichlidae
Oreochromis macrochir BIFZE040 17 Region Ankazobe Freshwater Introduced Cichlidae
Oreochromis macrochir BIFZE037 17 Region Ankazobe Freshwater Introduced Cichlidae
Oreochromis macrochir BIFZE035 17 Region Ankazobe Freshwater Introduced Cichlidae
Oreochromis aureus DBTM082 16 Region Atsinanana Parc Ivoloina Freshwater Introduced Cichlidae
Oreochromis aureus RDR0199 Andranobevava Freshwater Introduced Cichlidae
Oreochromis aureus RDR0209 Andranobevava Freshwater Introduced Cichlidae
97
Oreochromis aureus DBTMB093 17 Region Atsinanana Site Matera etang de M Zarre Freshwater Introduced Cichlidae
Oreochromis aureus DBTM019 16 Region Analamanga MilaSoa Freshwater Introduced Cichlidae
0
Oreochromis aureus BIFZE019 17 Region Toamasina MIDEM Freshwater Introduced Cichlidae
Coptodon zillii RDR0144 Vavarano Ankorondrano Ambany andrefan i Bemasoandro Freshwater Introduced Cichlidae
Coptodon zillii RDR0140 Vavarano Ankorondrano Ambany andrefan i Bemasoandro Freshwater Introduced Cichlidae
83
Coptodon zillii RDR0148 Vavarano Ankorondrano Ambany andrefan i Bemasoandro Freshwater Introduced Cichlidae
Coptodon zillii RDR0149 Vavarano Ankorondrano Ambany andrefan i Bemasoandro Freshwater Introduced Cichlidae
Coptodon zillii RDR0306 Androtra Freshwater Introduced Cichlidae
Coptodon zillii RDR0305 Androtra Freshwater Introduced Cichlidae
Coptodon rendalli RDR0808 Amparihibe Freshwater Introduced Cichlidae
Coptodon rendalli RDR0802 Amparihibe Freshwater Introduced Cichlidae
Coptodon rendalli FGMV 2002 F B40 Nosy Be Lake Amparihimirahavavy Freshwater Introduced Cichlidae
Coptodon rendalli FGMV 2002 F B39 Nosy Be Lake Amparihimirahavavy Freshwater Introduced Cichlidae
Coptodon rendalli RDR0771 Antsimony Freshwater Introduced Cichlidae
Coptodon rendalli FGMV 2002 F B41 Nosy Be Lake Amparihimirahavavy Freshwater Introduced Cichlidae
Coptodon rendalli RDR1085 Anjamanohatse Freshwater Introduced Cichlidae
Coptodon rendalli RDR0807 Amparihibe Freshwater Introduced Cichlidae
100
Coptodon rendalli RDR0801 Amparihibe Freshwater Introduced Cichlidae
Coptodon rendalli RDR0800 Amparihibe Freshwater Introduced Cichlidae
74
Coptodon rendalli RDR0799 Amparihibe Freshwater Introduced Cichlidae
Coptodon rendalli RDR0798 Amparihibe Freshwater Introduced Cichlidae
Coptodon rendalli RDR0783 Ambalavato Freshwater Introduced Cichlidae
Coptodon rendalli RDR0774 Antsimony Freshwater Introduced Cichlidae

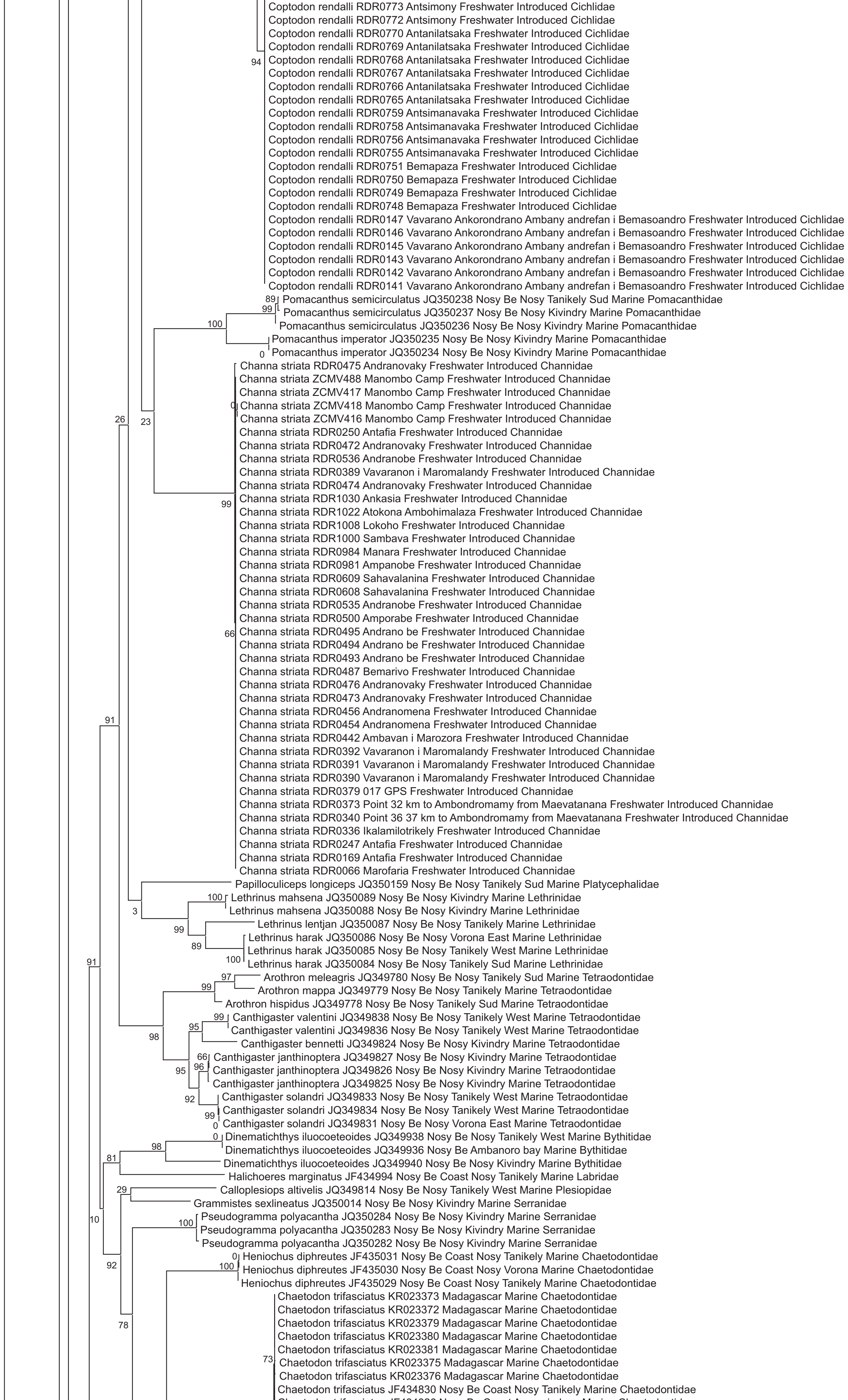
Coptodon rendalli RDR0773 Antsimony Freshwater Introduced Cichlidae
Coptodon rendalli RDR0772 Antsimony Freshwater Introduced Cichlidae
Coptodon rendalli RDR0770 Antanilatsaka Freshwater Introduced Cichlidae
Coptodon rendalli RDR0769 Antanilatsaka Freshwater Introduced Cichlidae
Coptodon rendalli RDR0768 Antanilatsaka Freshwater Introduced Cichlidae
Coptodon rendalli RDR0767 Antanilatsaka Freshwater Introduced Cichlidae
Coptodon rendalli RDR0766 Antanilatsaka Freshwater Introduced Cichlidae
Coptodon rendalli RDR0765 Antanilatsaka Freshwater Introduced Cichlidae
Coptodon rendalli RDR0759 Antsimanavaka Freshwater Introduced Cichlidae
Coptodon rendalli RDR0758 Antsimanavaka Freshwater Introduced Cichlidae
Coptodon rendalli RDR0756 Antsimanavaka Freshwater Introduced Cichlidae
Coptodon rendalli RDR0755 Antsimanavaka Freshwater Introduced Cichlidae
Coptodon rendalli RDR0751 Bemapaza Freshwater Introduced Cichlidae
Coptodon rendalli RDR0750 Bemapaza Freshwater Introduced Cichlidae
Coptodon rendalli RDR0749 Bemapaza Freshwater Introduced Cichlidae
Coptodon rendalli RDR0748 Bemapaza Freshwater Introduced Cichlidae
Coptodon rendalli RDR0147 Vavarano Ankorondrano Ambany andrefan i Bemasoandro Freshwater Introduced Cichlidae
Coptodon rendalli RDR0146 Vavarano Ankorondrano Ambany andrefan i Bemasoandro Freshwater Introduced Cichlidae
Coptodon rendalli RDR0145 Vavarano Ankorondrano Ambany andrefan i Bemasoandro Freshwater Introduced Cichlidae
Coptodon rendalli RDR0143 Vavarano Ankorondrano Ambany andrefan i Bemasoandro Freshwater Introduced Cichlidae
Coptodon rendalli RDR0142 Vavarano Ankorondrano Ambany andrefan i Bemasoandro Freshwater Introduced Cichlidae
Coptodon rendalli RDR0141 Vavarano Ankorondrano Ambany andrefan i Bemasoandro Freshwater Introduced Cichlidae
Pomacanthus semicirculatus JQ350238 Nosy Be Nosy Tanikely Sud Marine Pomacanthidae
Pomacanthus semicirculatus JQ350237 Nosy Be Nosy Kivindry Marine Pomacanthidae
Pomacanthus semicirculatus JQ350236 Nosy Be Nosy Kivindry Marine Pomacanthidae
Pomacanthus imperator JQ350235 Nosy Be Nosy Kivindry Marine Pomacanthidae
Pomacanthus imperator JQ350234 Nosy Be Nosy Kivindry Marine Pomacanthidae
Channa striata RDR0475 Andranovaky Freshwater Introduced Channidae
Channa striata ZCMV488 Manombo Camp Freshwater Introduced Channidae
Channa striata ZCMV417 Manombo Camp Freshwater Introduced Channidae
Channa striata ZCMV418 Manombo Camp Freshwater Introduced Channidae
Channa striata ZCMV416 Manombo Camp Freshwater Introduced Channidae
Channa striata RDR0250 Antafia Freshwater Introduced Channidae
Channa striata RDR0472 Andranovaky Freshwater Introduced Channidae
Channa striata RDR0536 Andranobe Freshwater Introduced Channidae
Channa striata RDR0389 Vavaranon i Maromalandy Freshwater Introduced Channidae
Channa striata RDR0474 Andranovaky Freshwater Introduced Channidae
Channa striata RDR1030 Ankasia Freshwater Introduced Channidae
Channa striata RDR1022 Atokona Ambohimalaza Freshwater Introduced Channidae
Channa striata RDR1008 Lokoho Freshwater Introduced Channidae
Channa striata RDR1000 Sambava Freshwater Introduced Channidae
Channa striata RDR0984 Manara Freshwater Introduced Channidae
Channa striata RDR0981 Ampanobe Freshwater Introduced Channidae
Channa striata RDR0609 Sahavalanina Freshwater Introduced Channidae
Channa striata RDR0608 Sahavalanina Freshwater Introduced Channidae
Channa striata RDR0535 Andranobe Freshwater Introduced Channidae
Channa striata RDR0500 Amporabe Freshwater Introduced Channidae
Channa striata RDR0495 Andrano be Freshwater Introduced Channidae
Channa striata RDR0494 Andrano be Freshwater Introduced Channidae
Channa striata RDR0493 Andrano be Freshwater Introduced Channidae
Channa striata RDR0487 Bemarivo Freshwater Introduced Channidae
Channa striata RDR0476 Andranovaky Freshwater Introduced Channidae
Channa striata RDR0473 Andranovaky Freshwater Introduced Channidae
Channa striata RDR0456 Andranomena Freshwater Introduced Channidae
Channa striata RDR0454 Andranomena Freshwater Introduced Channidae
Channa striata RDR0442 Ambavan i Marozora Freshwater Introduced Channidae
Channa striata RDR0392 Vavaranon i Maromalandy Freshwater Introduced Channidae
Channa striata RDR0391 Vavaranon i Maromalandy Freshwater Introduced Channidae
Channa striata RDR0390 Vavaranon i Maromalandy Freshwater Introduced Channidae
Channa striata RDR0379 017 GPS Freshwater Introduced Channidae
Channa striata RDR0373 Point 32 km to Ambondromamy from Maevatanana Freshwater Introduced Channidae
Channa striata RDR0340 Point 36 37 km to Ambondromamy from Maevatanana Freshwater Introduced Channidae
Channa striata RDR0336 Ikalamilotrikely Freshwater Introduced Channidae
Channa striata RDR0247 Antafia Freshwater Introduced Channidae
Channa striata RDR0169 Antafia Freshwater Introduced Channidae
Channa striata RDR0066 Marofaria Freshwater Introduced Channidae
Papilloculiceps longiceps JQ350159 Nosy Be Nosy Tanikely Sud Marine Platycephalidae
Lethrinus mahsena JQ350089 Nosy Be Nosy Kivindry Marine Lethrinidae
Lethrinus mahsena JQ350088 Nosy Be Nosy Kivindry Marine Lethrinidae
Lethrinus lentjan JQ350087 Nosy Be Nosy Tanikely Marine Lethrinidae
Lethrinus harak JQ350086 Nosy Be Nosy Vorona East Marine Lethrinidae
Lethrinus harak JQ350085 Nosy Be Nosy Tanikely West Marine Lethrinidae
Lethrinus harak JQ350084 Nosy Be Nosy Tanikely Sud Marine Lethrinidae
Arothron meleagris JQ349780 Nosy Be Nosy Tanikely Sud Marine Tetraodontidae
Arothron mappa JQ349779 Nosy Be Nosy Tanikely Marine Tetraodontidae
Arothron hispidus JQ349778 Nosy Be Nosy Tanikely Sud Marine Tetraodontidae
Canthigaster valentini JQ349838 Nosy Be Nosy Tanikely West Marine Tetraodontidae
Canthigaster valentini JQ349836 Nosy Be Nosy Tanikely West Marine Tetraodontidae
Canthigaster bennetti JQ349824 Nosy Be Nosy Kivindry Marine Tetraodontidae
Canthigaster janthinoptera JQ349827 Nosy Be Nosy Kivindry Marine Tetraodontidae
Canthigaster janthinoptera JQ349826 Nosy Be Nosy Kivindry Marine Tetraodontidae
Canthigaster janthinoptera JQ349825 Nosy Be Nosy Kivindry Marine Tetraodontidae
Canthigaster solandri JQ349833 Nosy Be Nosy Tanikely West Marine Tetraodontidae
Canthigaster solandri JQ349834 Nosy Be Nosy Tanikely West Marine Tetraodontidae
Canthigaster solandri JQ349831 Nosy Be Nosy Vorona East Marine Tetraodontidae
Dinematichthys iluocoeteoides JQ349938 Nosy Be Nosy Tanikely West Marine Bythitidae
Dinematichthys iluocoeteoides JQ349936 Nosy Be Ambanoro bay Marine Bythitidae
Dinematichthys iluocoeteoides JQ349940 Nosy Be Nosy Kivindry Marine Bythitidae
Halichoeres marginatus JF434994 Nosy Be Coast Nosy Tanikely Marine Labridae
Calloplesiops altivelis JQ349814 Nosy Be Nosy Tanikely West Marine Plesiopidae
Grammistes sexlineatus JQ350014 Nosy Be Nosy Kivindry Marine Serranidae
Pseudogramma polyacantha JQ350284 Nosy Be Nosy Kivindry Marine Serranidae
Pseudogramma polyacantha JQ350283 Nosy Be Nosy Kivindry Marine Serranidae
Pseudogramma polyacantha JQ350282 Nosy Be Nosy Kivindry Marine Serranidae
Heniochus diphreutes JF435031 Nosy Be Coast Nosy Tanikely Marine Chaetodontidae
Heniochus diphreutes JF435030 Nosy Be Coast Nosy Vorona Marine Chaetodontidae
Heniochus diphreutes JF435029 Nosy Be Coast Nosy Tanikely Marine Chaetodontidae
Chaetodon trifasciatus KR023373 Madagascar Marine Chaetodontidae
Chaetodon trifasciatus KR023372 Madagascar Marine Chaetodontidae
Chaetodon trifasciatus KR023379 Madagascar Marine Chaetodontidae
Chaetodon trifasciatus KR023380 Madagascar Marine Chaetodontidae
Chaetodon trifasciatus KR023381 Madagascar Marine Chaetodontidae
Chaetodon trifasciatus KR023375 Madagascar Marine Chaetodontidae
Chaetodon trifasciatus KR023376 Madagascar Marine Chaetodontidae
Chaetodon trifasciatus JF434830 Nosy Be Coast Nosy Tanikely Marine Chaetodontidae

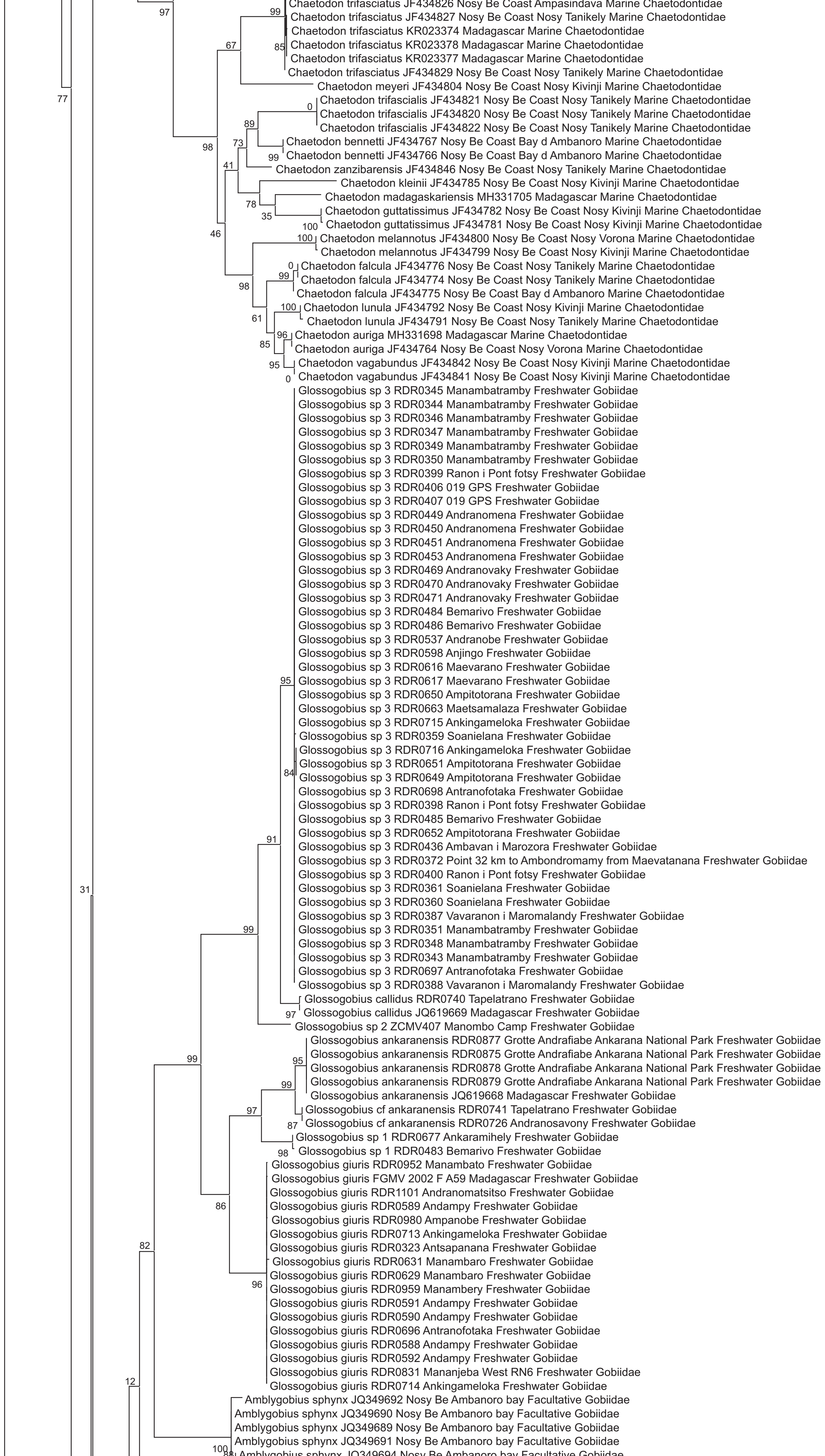

Chaetodon trifasciatus JF434826 Nosy Be Coast Ampasindava Marine Chaetodontidae
Chaetodon trifasciatus JF434827 Nosy Be Coast Nosy Tanikely Marine Chaetodontidae
Chaetodon trifasciatus KR023374 Madagascar Marine Chaetodontidae
Chaetodon trifasciatus KR023378 Madagascar Marine Chaetodontidae
Chaetodon trifasciatus KR023377 Madagascar Marine Chaetodontidae
Chaetodon trifasciatus JF434829 Nosy Be Coast Nosy Tanikely Marine Chaetodontidae
Chaetodon meyeri JF434804 Nosy Be Coast Nosy Kivinji Marine Chaetodontidae
Chaetodon trifascialis JF434821 Nosy Be Coast Nosy Tanikely Marine Chaetodontidae
Chaetodon trifascialis JF434820 Nosy Be Coast Nosy Tanikely Marine Chaetodontidae
Chaetodon trifascialis JF434822 Nosy Be Coast Nosy Tanikely Marine Chaetodontidae
Chaetodon bennetti JF434767 Nosy Be Coast Bay d Ambanoro Marine Chaetodontidae
Chaetodon bennetti JF434766 Nosy Be Coast Bay d Ambanoro Marine Chaetodontidae
Chaetodon zanzibarensis JF434846 Nosy Be Coast Nosy Tanikely Marine Chaetodontidae
Chaetodon kleinii JF434785 Nosy Be Coast Nosy Kivinji Marine Chaetodontidae
Chaetodon madagaskariensis MH331705 Madagascar Marine Chaetodontidae
Chaetodon guttatissimus JF434782 Nosy Be Coast Nosy Kivinji Marine Chaetodontidae
Chaetodon guttatissimus JF434781 Nosy Be Coast Nosy Kivinji Marine Chaetodontidae
Chaetodon melannotus JF434800 Nosy Be Coast Nosy Vorona Marine Chaetodontidae
Chaetodon melannotus JF434799 Nosy Be Coast Nosy Kivinji Marine Chaetodontidae
Chaetodon falcula JF434776 Nosy Be Coast Nosy Tanikely Marine Chaetodontidae
Chaetodon falcula JF434774 Nosy Be Coast Nosy Tanikely Marine Chaetodontidae
Chaetodon falcula JF434775 Nosy Be Coast Bay d Ambanoro Marine Chaetodontidae
Chaetodon lunula JF434792 Nosy Be Coast Nosy Kivinji Marine Chaetodontidae
Chaetodon lunula JF434791 Nosy Be Coast Nosy Tanikely Marine Chaetodontidae
Chaetodon auriga MH331698 Madagascar Marine Chaetodontidae
Chaetodon auriga JF434764 Nosy Be Coast Nosy Vorona Marine Chaetodontidae
Chaetodon vagabundus JF434842 Nosy Be Coast Nosy Kivinji Marine Chaetodontidae
Chaetodon vagabundus JF434841 Nosy Be Coast Nosy Kivinji Marine Chaetodontidae
Glossogobius sp 3 RDR0345 Manambatramby Freshwater Gobiidae
Glossogobius sp 3 RDR0344 Manambatramby Freshwater Gobiidae
Glossogobius sp 3 RDR0346 Manambatramby Freshwater Gobiidae
Glossogobius sp 3 RDR0347 Manambatramby Freshwater Gobiidae
Glossogobius sp 3 RDR0349 Manambatramby Freshwater Gobiidae
Glossogobius sp 3 RDR0350 Manambatramby Freshwater Gobiidae
Glossogobius sp 3 RDR0399 Ranon i Pont fotsy Freshwater Gobiidae
Glossogobius sp 3 RDR0406 019 GPS Freshwater Gobiidae
Glossogobius sp 3 RDR0407 019 GPS Freshwater Gobiidae
Glossogobius sp 3 RDR0449 Andranomena Freshwater Gobiidae
Glossogobius sp 3 RDR0450 Andranomena Freshwater Gobiidae
Glossogobius sp 3 RDR0451 Andranomena Freshwater Gobiidae
Glossogobius sp 3 RDR0453 Andranomena Freshwater Gobiidae
Glossogobius sp 3 RDR0469 Andranovaky Freshwater Gobiidae
Glossogobius sp 3 RDR0470 Andranovaky Freshwater Gobiidae
Glossogobius sp 3 RDR0471 Andranovaky Freshwater Gobiidae
Glossogobius sp 3 RDR0484 Bemarivo Freshwater Gobiidae
Glossogobius sp 3 RDR0486 Bemarivo Freshwater Gobiidae
Glossogobius sp 3 RDR0537 Andranobe Freshwater Gobiidae
Glossogobius sp 3 RDR0598 Anjingo Freshwater Gobiidae
Glossogobius sp 3 RDR0616 Maevarano Freshwater Gobiidae
Glossogobius sp 3 RDR0617 Maevarano Freshwater Gobiidae
Glossogobius sp 3 RDR0650 Ampitotorana Freshwater Gobiidae
Glossogobius sp 3 RDR0663 Maetsamalaza Freshwater Gobiidae
Glossogobius sp 3 RDR0715 Ankingameloka Freshwater Gobiidae
Glossogobius sp 3 RDR0359 Soanielana Freshwater Gobiidae
Glossogobius sp 3 RDR0716 Ankingameloka Freshwater Gobiidae
Glossogobius sp 3 RDR0651 Ampitotorana Freshwater Gobiidae
Glossogobius sp 3 RDR0649 Ampitotorana Freshwater Gobiidae
Glossogobius sp 3 RDR0698 Antranofotaka Freshwater Gobiidae
Glossogobius sp 3 RDR0398 Ranon i Pont fotsy Freshwater Gobiidae
Glossogobius sp 3 RDR0485 Bemarivo Freshwater Gobiidae
Glossogobius sp 3 RDR0652 Ampitotorana Freshwater Gobiidae
Glossogobius sp 3 RDR0436 Ambavan i Marozora Freshwater Gobiidae
Glossogobius sp 3 RDR0372 Point 32 km to Ambondromamy from Maevatanana Freshwater Gobiidae
Glossogobius sp 3 RDR0400 Ranon i Pont fotsy Freshwater Gobiidae
Glossogobius sp 3 RDR0361 Soanielana Freshwater Gobiidae
Glossogobius sp 3 RDR0360 Soanielana Freshwater Gobiidae
Glossogobius sp 3 RDR0387 Vavaranon i Maromalandy Freshwater Gobiidae
Glossogobius sp 3 RDR0351 Manambatramby Freshwater Gobiidae
Glossogobius sp 3 RDR0348 Manambatramby Freshwater Gobiidae
Glossogobius sp 3 RDR0343 Manambatramby Freshwater Gobiidae
Glossogobius sp 3 RDR0697 Antranofotaka Freshwater Gobiidae
Glossogobius sp 3 RDR0388 Vavaranon i Maromalandy Freshwater Gobiidae
Glossogobius callidus RDR0740 Tapelatrano Freshwater Gobiidae
Glossogobius callidus JQ619669 Madagascar Freshwater Gobiidae
Glossogobius sp 2 ZCMV407 Manombo Camp Freshwater Gobiidae
Glossogobius ankaranensis RDR0877 Grotte Andrafiabe Ankarana National Park Freshwater Gobiidae
Glossogobius ankaranensis RDR0875 Grotte Andrafiabe Ankarana National Park Freshwater Gobiidae
Glossogobius ankaranensis RDR0878 Grotte Andrafiabe Ankarana National Park Freshwater Gobiidae
Glossogobius ankaranensis RDR0879 Grotte Andrafiabe Ankarana National Park Freshwater Gobiidae
Glossogobius ankaranensis JQ619668 Madagascar Freshwater Gobiidae
Glossogobius cf ankaranensis RDR0741 Tapelatrano Freshwater Gobiidae
Glossogobius cf ankaranensis RDR0726 Andranosavony Freshwater Gobiidae
Glossogobius sp 1 RDR0677 Ankaramihely Freshwater Gobiidae
Glossogobius sp 1 RDR0483 Bemarivo Freshwater Gobiidae
Glossogobius giuris RDR0952 Manambato Freshwater Gobiidae
Glossogobius giuris FGMV 2002 F A59 Madagascar Freshwater Gobiidae
Glossogobius giuris RDR1101 Andranomatsitso Freshwater Gobiidae
Glossogobius giuris RDR0589 Andampy Freshwater Gobiidae
Glossogobius giuris RDR0980 Ampanobe Freshwater Gobiidae
Glossogobius giuris RDR0713 Ankingameloka Freshwater Gobiidae
Glossogobius giuris RDR0323 Antsapanana Freshwater Gobiidae
Glossogobius giuris RDR0631 Manambaro Freshwater Gobiidae
Glossogobius giuris RDR0629 Manambaro Freshwater Gobiidae
Glossogobius giuris RDR0959 Manambery Freshwater Gobiidae
Glossogobius giuris RDR0591 Andampy Freshwater Gobiidae
Glossogobius giuris RDR0590 Andampy Freshwater Gobiidae
Glossogobius giuris RDR0696 Antranofotaka Freshwater Gobiidae
Glossogobius giuris RDR0588 Andampy Freshwater Gobiidae
Glossogobius giuris RDR0592 Andampy Freshwater Gobiidae
Glossogobius giuris RDR0831 Mananjeba West RN6 Freshwater Gobiidae
Glossogobius giuris RDR0714 Ankingameloka Freshwater Gobiidae
Amblygobius sphynx JQ349692 Nosy Be Ambanoro bay Facultative Gobiidae
Amblygobius sphynx JQ349690 Nosy Be Ambanoro bay Facultative Gobiidae
Amblygobius sphynx JQ349689 Nosy Be Ambanoro bay Facultative Gobiidae
Amblygobius sphynx JQ349691 Nosy Be Ambanoro bay Facultative Gobiidae
Amblygobius sphynx JQ349694 Nosy Be Ambanoro bay Facultative Gobiidae

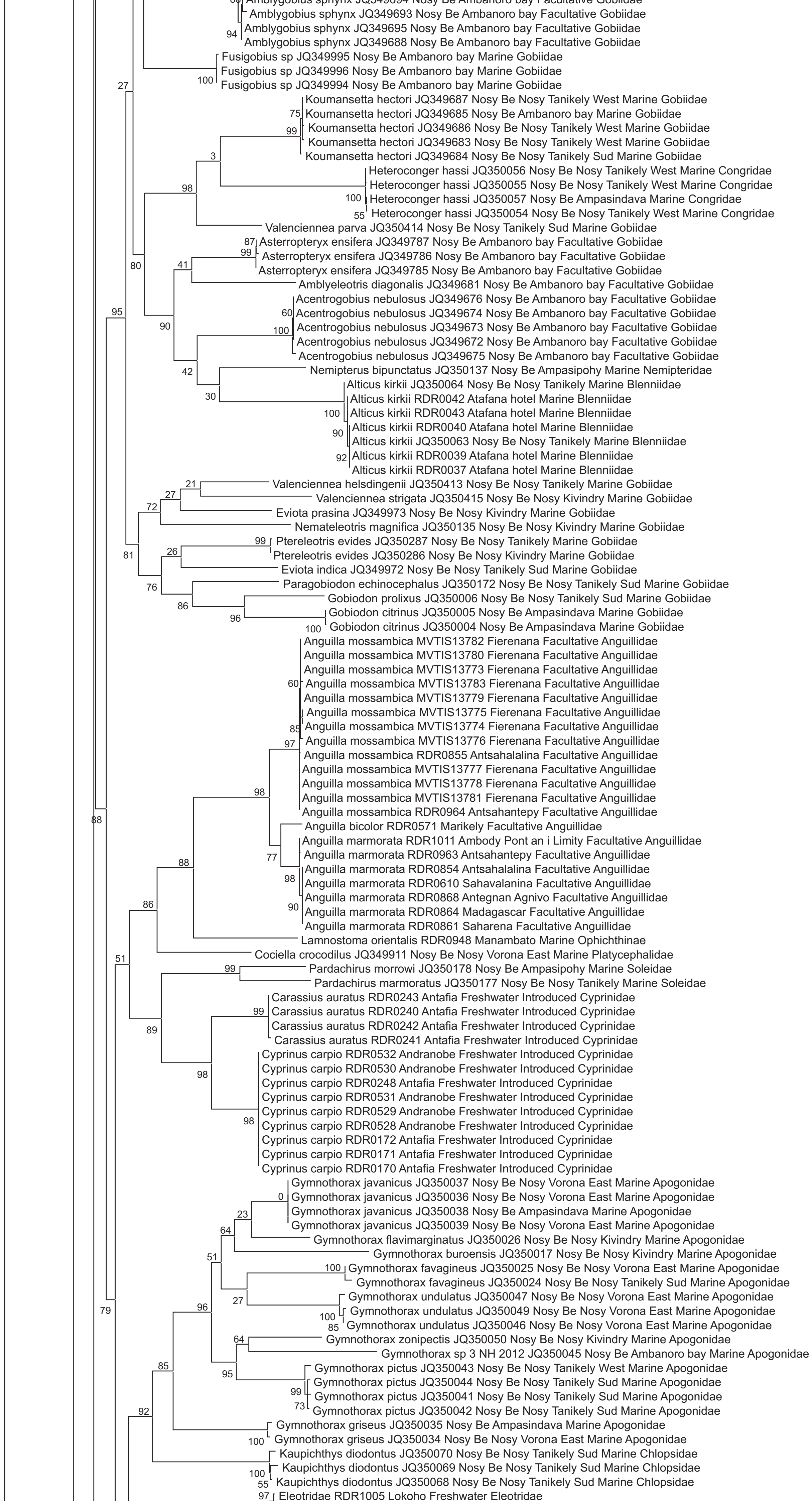
Amblygobius sphynx JQ349693 Nosy Be Ambanoro bay Facultative Gobiidae
Amblygobius sphynx JQ349695 Nosy Be Ambanoro bay Facultative Gobiidae
Amblygobius sphynx JQ349688 Nosy Be Ambanoro bay Facultative Gobiidae
Fusigobius sp JQ349995 Nosy Be Ambanoro bay Marine Gobiidae
Fusigobius sp JQ349996 Nosy Be Ambanoro bay Marine Gobiidae
Fusigobius sp JQ349994 Nosy Be Ambanoro bay Marine Gobiidae
Koumansetta hectori JQ349687 Nosy Be Nosy Tanikely West Marine Gobiidae
Koumansetta hectori JQ349685 Nosy Be Ambanoro bay Marine Gobiidae
Koumansetta hectori JQ349686 Nosy Be Nosy Tanikely West Marine Gobiidae
Koumansetta hectori JQ349683 Nosy Be Nosy Tanikely West Marine Gobiidae
Koumansetta hectori JQ349684 Nosy Be Nosy Tanikely Sud Marine Gobiidae
Heteroconger hassi JQ350056 Nosy Be Nosy Tanikely West Marine Congridae
Heteroconger hassi JQ350055 Nosy Be Nosy Tanikely West Marine Congridae
Heteroconger hassi JQ350057 Nosy Be Ampasindava Marine Congridae
Heteroconger hassi JQ350054 Nosy Be Nosy Tanikely West Marine Congridae
Valenciennea parva JQ350414 Nosy Be Nosy Tanikely Sud Marine Gobiidae
Asterropteryx ensifera JQ349787 Nosy Be Ambanoro bay Facultative Gobiidae
Asterropteryx ensifera JQ349786 Nosy Be Ambanoro bay Facultative Gobiidae
Asterropteryx ensifera JQ349785 Nosy Be Ambanoro bay Facultative Gobiidae
Amblyeleotris diagonalis JQ349681 Nosy Be Ambanoro bay Facultative Gobiidae
Acentrogobius nebulosus JQ349676 Nosy Be Ambanoro bay Facultative Gobiidae
Acentrogobius nebulosus JQ349674 Nosy Be Ambanoro bay Facultative Gobiidae
Acentrogobius nebulosus JQ349673 Nosy Be Ambanoro bay Facultative Gobiidae
Acentrogobius nebulosus JQ349672 Nosy Be Ambanoro bay Facultative Gobiidae
Acentrogobius nebulosus JQ349675 Nosy Be Ambanoro bay Facultative Gobiidae
Nemipterus bipunctatus JQ350137 Nosy Be Ampasipohy Marine Nemipteridae
Alticus kirkii JQ350064 Nosy Be Nosy Tanikely Marine Blenniidae
Alticus kirkii RDR0042 Atafana hotel Marine Blenniidae
Alticus kirkii RDR0043 Atafana hotel Marine Blenniidae
Alticus kirkii RDR0040 Atafana hotel Marine Blenniidae
Alticus kirkii JQ350063 Nosy Be Nosy Tanikely Marine Blenniidae
Alticus kirkii RDR0039 Atafana hotel Marine Blenniidae
Alticus kirkii RDR0037 Atafana hotel Marine Blenniidae
Valenciennea helsdingenii JQ350413 Nosy Be Nosy Tanikely Marine Gobiidae
Valenciennea strigata JQ350415 Nosy Be Nosy Kivindry Marine Gobiidae
Eviota prasina JQ349973 Nosy Be Nosy Kivindry Marine Gobiidae
Nemateleotris magnifica JQ350135 Nosy Be Nosy Kivindry Marine Gobiidae
Ptereleotris evides JQ350287 Nosy Be Nosy Tanikely Marine Gobiidae
Ptereleotris evides JQ350286 Nosy Be Nosy Kivindry Marine Gobiidae
Eviota indica JQ349972 Nosy Be Nosy Tanikely Sud Marine Gobiidae
Paragobiodon echinocephalus JQ350172 Nosy Be Nosy Tanikely Sud Marine Gobiidae
Gobiodon prolixus JQ350006 Nosy Be Nosy Tanikely Sud Marine Gobiidae
Gobiodon citrinus JQ350005 Nosy Be Ampasindava Marine Gobiidae
Gobiodon citrinus JQ350004 Nosy Be Ampasindava Marine Gobiidae
Anguilla mossambica MVTIS13782 Fierenana Facultative Anguillidae
Anguilla mossambica MVTIS13780 Fierenana Facultative Anguillidae
Anguilla mossambica MVTIS13773 Fierenana Facultative Anguillidae
Anguilla mossambica MVTIS13783 Fierenana Facultative Anguillidae
Anguilla mossambica MVTIS13779 Fierenana Facultative Anguillidae
Anguilla mossambica MVTIS13775 Fierenana Facultative Anguillidae
Anguilla mossambica MVTIS13774 Fierenana Facultative Anguillidae
Anguilla mossambica MVTIS13776 Fierenana Facultative Anguillidae
Anguilla mossambica RDR0855 Antsahalalina Facultative Anguillidae
Anguilla mossambica MVTIS13777 Fierenana Facultative Anguillidae
Anguilla mossambica MVTIS13778 Fierenana Facultative Anguillidae
Anguilla mossambica MVTIS13781 Fierenana Facultative Anguillidae
Anguilla mossambica RDR0964 Antsahantepy Facultative Anguillidae
Anguilla bicolor RDR0571 Marikely Facultative Anguillidae
Anguilla marmorata RDR1011 Ambody Pont an i Limity Facultative Anguillidae
Anguilla marmorata RDR0963 Antsahantepy Facultative Anguillidae
Anguilla marmorata RDR0854 Antsahalalina Facultative Anguillidae
Anguilla marmorata RDR0610 Sahavalanina Facultative Anguillidae
Anguilla marmorata RDR0868 Antegnan Agnivo Facultative Anguillidae
Anguilla marmorata RDR0864 Madagascar Facultative Anguillidae
Anguilla marmorata RDR0861 Saharena Facultative Anguillidae
Lamnostoma orientalis RDR0948 Manambato Marine Ophichthinae
Cociella crocodilus JQ349911 Nosy Be Nosy Vorona East Marine Platycephalidae
Pardachirus morrowi JQ350178 Nosy Be Ampasipohy Marine Soleidae
Pardachirus marmoratus JQ350177 Nosy Be Nosy Tanikely Marine Soleidae
Carassius auratus RDR0243 Antafia Freshwater Introduced Cyprinidae
Carassius auratus RDR0240 Antafia Freshwater Introduced Cyprinidae
Carassius auratus RDR0242 Antafia Freshwater Introduced Cyprinidae
Carassius auratus RDR0241 Antafia Freshwater Introduced Cyprinidae
Cyprinus carpio RDR0532 Andranobe Freshwater Introduced Cyprinidae
Cyprinus carpio RDR0530 Andranobe Freshwater Introduced Cyprinidae
Cyprinus carpio RDR0248 Antafia Freshwater Introduced Cyprinidae
Cyprinus carpio RDR0531 Andranobe Freshwater Introduced Cyprinidae
Cyprinus carpio RDR0529 Andranobe Freshwater Introduced Cyprinidae
Cyprinus carpio RDR0528 Andranobe Freshwater Introduced Cyprinidae
Cyprinus carpio RDR0172 Antafia Freshwater Introduced Cyprinidae
Cyprinus carpio RDR0171 Antafia Freshwater Introduced Cyprinidae
Cyprinus carpio RDR0170 Antafia Freshwater Introduced Cyprinidae
Gymnothorax javanicus JQ350037 Nosy Be Nosy Vorona East Marine Apogonidae
Gymnothorax javanicus JQ350036 Nosy Be Nosy Vorona East Marine Apogonidae
Gymnothorax javanicus JQ350038 Nosy Be Ampasindava Marine Apogonidae
Gymnothorax javanicus JQ350039 Nosy Be Nosy Vorona East Marine Apogonidae
Gymnothorax flavimarginatus JQ350026 Nosy Be Nosy Kivindry Marine Apogonidae
Gymnothorax buroensis JQ350017 Nosy Be Nosy Kivindry Marine Apogonidae
Gymnothorax favagineus JQ350025 Nosy Be Nosy Vorona East Marine Apogonidae
Gymnothorax favagineus JQ350024 Nosy Be Nosy Tanikely Sud Marine Apogonidae
Gymnothorax undulatus JQ350047 Nosy Be Nosy Vorona East Marine Apogonidae
Gymnothorax undulatus JQ350049 Nosy Be Nosy Vorona East Marine Apogonidae
Gymnothorax undulatus JQ350046 Nosy Be Nosy Vorona East Marine Apogonidae
Gymnothorax zonipectis JQ350050 Nosy Be Nosy Kivindry Marine Apogonidae
Gymnothorax sp 3 NH 2012 JQ350045 Nosy Be Ambanoro bay Marine Apogonidae
Gymnothorax pictus JQ350043 Nosy Be Nosy Tanikely West Marine Apogonidae
Gymnothorax pictus JQ350044 Nosy Be Nosy Tanikely Sud Marine Apogonidae
Gymnothorax pictus JQ350041 Nosy Be Nosy Tanikely Sud Marine Apogonidae
Gymnothorax pictus JQ350042 Nosy Be Nosy Tanikely Sud Marine Apogonidae
Gymnothorax griseus JQ350035 Nosy Be Ampasindava Marine Apogonidae
Gymnothorax griseus JQ350034 Nosy Be Nosy Vorona East Marine Apogonidae
Kaupichthys diodontus JQ350070 Nosy Be Nosy Tanikely Sud Marine Chlopsidae
Kaupichthys diodontus JQ350069 Nosy Be Nosy Tanikely Sud Marine Chlopsidae
Kaupichthys diodontus JQ350068 Nosy Be Nosy Tanikely Sud Marine Chlopsidae
Eleotridae RDR1005 Lokoho Freshwater Eleotridae

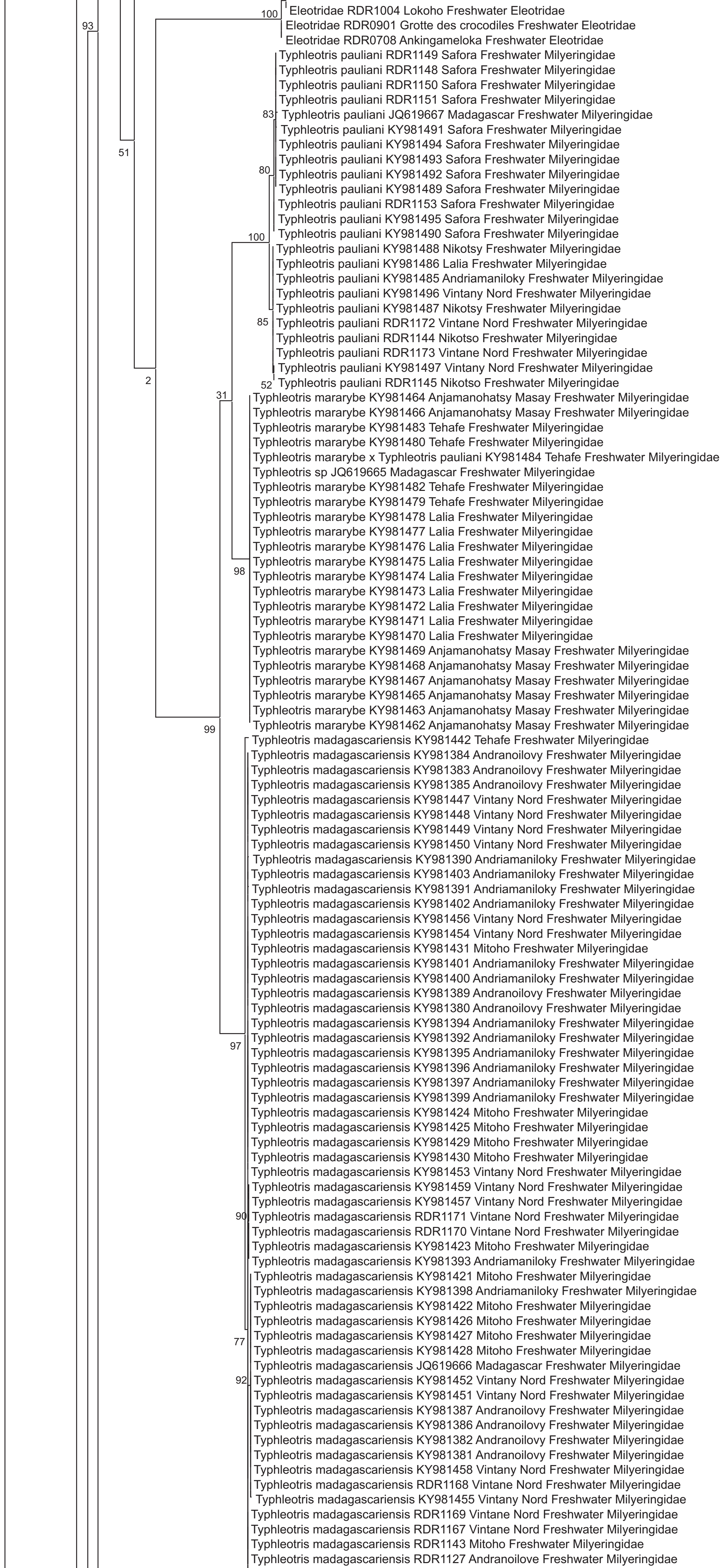
93
100
Eleotridae RDR1004 Lokoho Freshwater Eleotridae
Eleotridae RDR0901 Grotte des crocodiles Freshwater Eleotridae
Eleotridae RDR0708 Ankingameloka Freshwater Eleotridae
Typhleotris pauliani RDR1149 Safora Freshwater Milyeringidae
Typhleotris pauliani RDR1148 Safora Freshwater Milyeringidae
Typhleotris pauliani RDR1150 Safora Freshwater Milyeringidae
Typhleotris pauliani RDR1151 Safora Freshwater Milyeringidae
83
Typhleotris pauliani JQ619667 Madagascar Freshwater Milyeringidae
Typhleotris pauliani KY981491 Safora Freshwater Milyeringidae
Typhleotris pauliani KY981494 Safora Freshwater Milyeringidae
51
Typhleotris pauliani KY981493 Safora Freshwater Milyeringidae
80
Typhleotris pauliani KY981492 Safora Freshwater Milyeringidae
Typhleotris pauliani KY981489 Safora Freshwater Milyeringidae
Typhleotris pauliani RDR1153 Safora Freshwater Milyeringidae
Typhleotris pauliani KY981495 Safora Freshwater Milyeringidae
100
Typhleotris pauliani KY981490 Safora Freshwater Milyeringidae
Typhleotris pauliani KY981488 Nikotsy Freshwater Milyeringidae
Typhleotris pauliani KY981486 Lalia Freshwater Milyeringidae
Typhleotris pauliani KY981485 Andriamaniloky Freshwater Milyeringidae
Typhleotris pauliani KY981496 Vintany Nord Freshwater Milyeringidae
Typhleotris pauliani KY981487 Nikotsy Freshwater Milyeringidae
85
Typhleotris pauliani RDR1172 Vintane Nord Freshwater Milyeringidae
Typhleotris pauliani RDR1144 Nikotso Freshwater Milyeringidae
Typhleotris pauliani RDR1173 Vintane Nord Freshwater Milyeringidae
Typhleotris pauliani KY981497 Vintany Nord Freshwater Milyeringidae
2
52
Typhleotris pauliani RDR1145 Nikotso Freshwater Milyeringidae
31
Typhleotris mararybe KY981464 Anjamanohatsy Masay Freshwater Milyeringidae
Typhleotris mararybe KY981466 Anjamanohatsy Masay Freshwater Milyeringidae
Typhleotris mararybe KY981483 Tehafe Freshwater Milyeringidae
Typhleotris mararybe KY981480 Tehafe Freshwater Milyeringidae
Typhleotris mararybe x Typhleotris pauliani KY981484 Tehafe Freshwater Milyeringidae
Typhleotris sp JQ619665 Madagascar Freshwater Milyeringidae
Typhleotris mararybe KY981482 Tehafe Freshwater Milyeringidae
Typhleotris mararybe KY981479 Tehafe Freshwater Milyeringidae
Typhleotris mararybe KY981478 Lalia Freshwater Milyeringidae
Typhleotris mararybe KY981477 Lalia Freshwater Milyeringidae
Typhleotris mararybe KY981476 Lalia Freshwater Milyeringidae
Typhleotris mararybe KY981475 Lalia Freshwater Milyeringidae
98
Typhleotris mararybe KY981474 Lalia Freshwater Milyeringidae
Typhleotris mararybe KY981473 Lalia Freshwater Milyeringidae
Typhleotris mararybe KY981472 Lalia Freshwater Milyeringidae
Typhleotris mararybe KY981471 Lalia Freshwater Milyeringidae
Typhleotris mararybe KY981470 Lalia Freshwater Milyeringidae
Typhleotris mararybe KY981469 Anjamanohatsy Masay Freshwater Milyeringidae
Typhleotris mararybe KY981468 Anjamanohatsy Masay Freshwater Milyeringidae
Typhleotris mararybe KY981467 Anjamanohatsy Masay Freshwater Milyeringidae
Typhleotris mararybe KY981465 Anjamanohatsy Masay Freshwater Milyeringidae
Typhleotris mararybe KY981463 Anjamanohatsy Masay Freshwater Milyeringidae
Typhleotris mararybe KY981462 Anjamanohatsy Masay Freshwater Milyeringidae
99
Typhleotris madagascariensis KY981442 Tehafe Freshwater Milyeringidae
Typhleotris madagascariensis KY981384 Andranoilovy Freshwater Milyeringidae
Typhleotris madagascariensis KY981383 Andranoilovy Freshwater Milyeringidae
Typhleotris madagascariensis KY981385 Andranoilovy Freshwater Milyeringidae
Typhleotris madagascariensis KY981447 Vintany Nord Freshwater Milyeringidae
Typhleotris madagascariensis KY981448 Vintany Nord Freshwater Milyeringidae
Typhleotris madagascariensis KY981449 Vintany Nord Freshwater Milyeringidae
Typhleotris madagascariensis KY981450 Vintany Nord Freshwater Milyeringidae
Typhleotris madagascariensis KY981390 Andriamaniloky Freshwater Milyeringidae
Typhleotris madagascariensis KY981403 Andriamaniloky Freshwater Milyeringidae
Typhleotris madagascariensis KY981391 Andriamaniloky Freshwater Milyeringidae
Typhleotris madagascariensis KY981402 Andriamaniloky Freshwater Milyeringidae
Typhleotris madagascariensis KY981456 Vintany Nord Freshwater Milyeringidae
Typhleotris madagascariensis KY981454 Vintany Nord Freshwater Milyeringidae
Typhleotris madagascariensis KY981431 Mitoho Freshwater Milyeringidae
Typhleotris madagascariensis KY981401 Andriamaniloky Freshwater Milyeringidae
Typhleotris madagascariensis KY981400 Andriamaniloky Freshwater Milyeringidae
Typhleotris madagascariensis KY981389 Andranoilovy Freshwater Milyeringidae
Typhleotris madagascariensis KY981380 Andranoilovy Freshwater Milyeringidae
Typhleotris madagascariensis KY981394 Andriamaniloky Freshwater Milyeringidae
Typhleotris madagascariensis KY981392 Andriamaniloky Freshwater Milyeringidae
97
Typhleotris madagascariensis KY981395 Andriamaniloky Freshwater Milyeringidae
Typhleotris madagascariensis KY981396 Andriamaniloky Freshwater Milyeringidae
Typhleotris madagascariensis KY981397 Andriamaniloky Freshwater Milyeringidae
Typhleotris madagascariensis KY981399 Andriamaniloky Freshwater Milyeringidae
Typhleotris madagascariensis KY981424 Mitoho Freshwater Milyeringidae
Typhleotris madagascariensis KY981425 Mitoho Freshwater Milyeringidae
Typhleotris madagascariensis KY981429 Mitoho Freshwater Milyeringidae
Typhleotris madagascariensis KY981430 Mitoho Freshwater Milyeringidae
Typhleotris madagascariensis KY981453 Vintany Nord Freshwater Milyeringidae
Typhleotris madagascariensis KY981459 Vintany Nord Freshwater Milyeringidae
Typhleotris madagascariensis KY981457 Vintany Nord Freshwater Milyeringidae
90
Typhleotris madagascariensis RDR1171 Vintane Nord Freshwater Milyeringidae
Typhleotris madagascariensis RDR1170 Vintane Nord Freshwater Milyeringidae
Typhleotris madagascariensis KY981423 Mitoho Freshwater Milyeringidae
Typhleotris madagascariensis KY981393 Andriamaniloky Freshwater Milyeringidae
Typhleotris madagascariensis KY981421 Mitoho Freshwater Milyeringidae
Typhleotris madagascariensis KY981398 Andriamaniloky Freshwater Milyeringidae
Typhleotris madagascariensis KY981422 Mitoho Freshwater Milyeringidae
Typhleotris madagascariensis KY981426 Mitoho Freshwater Milyeringidae
Typhleotris madagascariensis KY981427 Mitoho Freshwater Milyeringidae
77
Typhleotris madagascariensis KY981428 Mitoho Freshwater Milyeringidae
Typhleotris madagascariensis JQ619666 Madagascar Freshwater Milyeringidae
92
Typhleotris madagascariensis KY981452 Vintany Nord Freshwater Milyeringidae
Typhleotris madagascariensis KY981451 Vintany Nord Freshwater Milyeringidae
Typhleotris madagascariensis KY981387 Andranoilovy Freshwater Milyeringidae
Typhleotris madagascariensis KY981386 Andranoilovy Freshwater Milyeringidae
Typhleotris madagascariensis KY981382 Andranoilovy Freshwater Milyeringidae
Typhleotris madagascariensis KY981381 Andranoilovy Freshwater Milyeringidae
Typhleotris madagascariensis KY981458 Vintany Nord Freshwater Milyeringidae
Typhleotris madagascariensis RDR1168 Vintane Nord Freshwater Milyeringidae
Typhleotris madagascariensis KY981455 Vintany Nord Freshwater Milyeringidae
Typhleotris madagascariensis RDR1169 Vintane Nord Freshwater Milyeringidae
Typhleotris madagascariensis RDR1167 Vintane Nord Freshwater Milyeringidae
Typhleotris madagascariensis RDR1143 Mitoho Freshwater Milyeringidae
Typhleotris madagascariensis RDR1127 Andranoilove Freshwater Milyeringidae

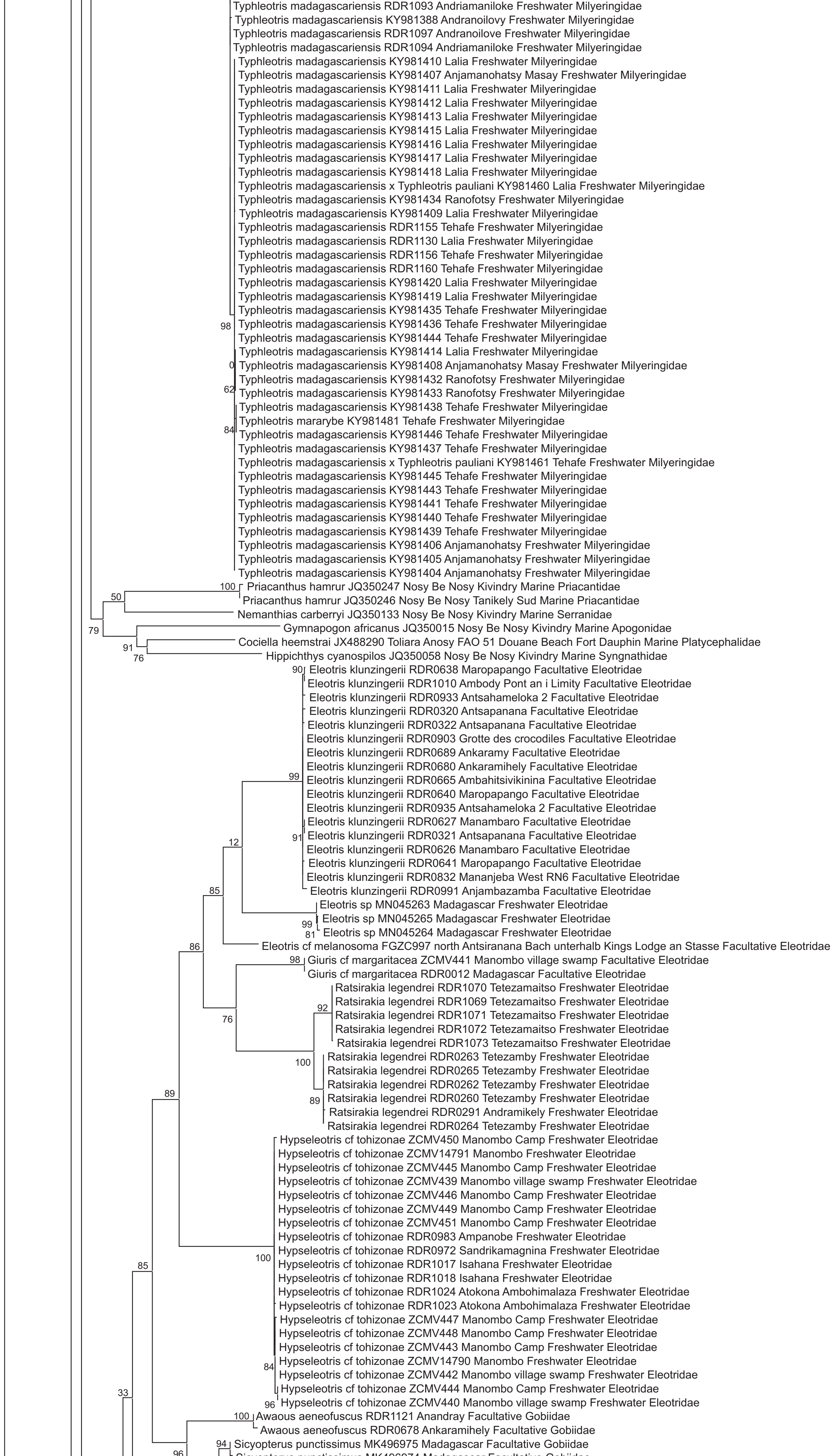

Typhleotris madagascariensis RDR1093 Andriamaniloke Freshwater Milyeringidae
Typhleotris madagascariensis KY981388 Andranoilovy Freshwater Milyeringidae
Typhleotris madagascariensis RDR1097 Andranoilove Freshwater Milyeringidae
Typhleotris madagascariensis RDR1094 Andriamaniloke Freshwater Milyeringidae
Typhleotris madagascariensis KY981410 Lalia Freshwater Milyeringidae
Typhleotris madagascariensis KY981407 Anjamanohatsy Masay Freshwater Milyeringidae
Typhleotris madagascariensis KY981411 Lalia Freshwater Milyeringidae
Typhleotris madagascariensis KY981412 Lalia Freshwater Milyeringidae
Typhleotris madagascariensis KY981413 Lalia Freshwater Milyeringidae
Typhleotris madagascariensis KY981415 Lalia Freshwater Milyeringidae
Typhleotris madagascariensis KY981416 Lalia Freshwater Milyeringidae
Typhleotris madagascariensis KY981417 Lalia Freshwater Milyeringidae
Typhleotris madagascariensis KY981418 Lalia Freshwater Milyeringidae
Typhleotris madagascariensis x Typhleotris pauliani KY981460 Lalia Freshwater Milyeringidae
Typhleotris madagascariensis KY981434 Ranofotsy Freshwater Milyeringidae
Typhleotris madagascariensis KY981409 Lalia Freshwater Milyeringidae
Typhleotris madagascariensis RDR1155 Tehafe Freshwater Milyeringidae
Typhleotris madagascariensis RDR1130 Lalia Freshwater Milyeringidae
Typhleotris madagascariensis RDR1156 Tehafe Freshwater Milyeringidae
Typhleotris madagascariensis RDR1160 Tehafe Freshwater Milyeringidae
Typhleotris madagascariensis KY981420 Lalia Freshwater Milyeringidae
Typhleotris madagascariensis KY981419 Lalia Freshwater Milyeringidae
Typhleotris madagascariensis KY981435 Tehafe Freshwater Milyeringidae
Typhleotris madagascariensis KY981436 Tehafe Freshwater Milyeringidae
Typhleotris madagascariensis KY981444 Tehafe Freshwater Milyeringidae
Typhleotris madagascariensis KY981414 Lalia Freshwater Milyeringidae
Typhleotris madagascariensis KY981408 Anjamanohatsy Masay Freshwater Milyeringidae
Typhleotris madagascariensis KY981432 Ranofotsy Freshwater Milyeringidae
Typhleotris madagascariensis KY981433 Ranofotsy Freshwater Milyeringidae
Typhleotris madagascariensis KY981438 Tehafe Freshwater Milyeringidae
Typhleotris mararybe KY981481 Tehafe Freshwater Milyeringidae
Typhleotris madagascariensis KY981446 Tehafe Freshwater Milyeringidae
Typhleotris madagascariensis KY981437 Tehafe Freshwater Milyeringidae
Typhleotris madagascariensis x Typhleotris pauliani KY981461 Tehafe Freshwater Milyeringidae
Typhleotris madagascariensis KY981445 Tehafe Freshwater Milyeringidae
Typhleotris madagascariensis KY981443 Tehafe Freshwater Milyeringidae
Typhleotris madagascariensis KY981441 Tehafe Freshwater Milyeringidae
Typhleotris madagascariensis KY981440 Tehafe Freshwater Milyeringidae
Typhleotris madagascariensis KY981439 Tehafe Freshwater Milyeringidae
Typhleotris madagascariensis KY981406 Anjamanohatsy Freshwater Milyeringidae
Typhleotris madagascariensis KY981405 Anjamanohatsy Freshwater Milyeringidae
Typhleotris madagascariensis KY981404 Anjamanohatsy Freshwater Milyeringidae
Priacanthus hamrur JQ350247 Nosy Be Nosy Kivindry Marine Priacantidae
Priacanthus hamrur JQ350246 Nosy Be Nosy Tanikely Sud Marine Priacantidae
Nemanthias carberryi JQ350133 Nosy Be Nosy Kivindry Marine Serranidae
Gymnapogon africanus JQ350015 Nosy Be Nosy Kivindry Marine Apogonidae
Cociella heemstrai JX488290 Toliara Anosy FAO 51 Douane Beach Fort Dauphin Marine Platycephalidae
Hippichthys cyanospilos JQ350058 Nosy Be Nosy Kivindry Marine Syngnathidae
Eleotris klunzingerii RDR0638 Maropapango Facultative Eleotridae
Eleotris klunzingerii RDR1010 Ambody Pont an i Limity Facultative Eleotridae
Eleotris klunzingerii RDR0933 Antsahameloka 2 Facultative Eleotridae
Eleotris klunzingerii RDR0320 Antsapanana Facultative Eleotridae
Eleotris klunzingerii RDR0322 Antsapanana Facultative Eleotridae
Eleotris klunzingerii RDR0903 Grotte des crocodiles Facultative Eleotridae
Eleotris klunzingerii RDR0689 Ankaramy Facultative Eleotridae
Eleotris klunzingerii RDR0680 Ankaramihely Facultative Eleotridae
Eleotris klunzingerii RDR0665 Ambahitsivikinina Facultative Eleotridae
Eleotris klunzingerii RDR0640 Maropapango Facultative Eleotridae
Eleotris klunzingerii RDR0935 Antsahameloka 2 Facultative Eleotridae
Eleotris klunzingerii RDR0627 Manambaro Facultative Eleotridae
Eleotris klunzingerii RDR0321 Antsapanana Facultative Eleotridae
Eleotris klunzingerii RDR0626 Manambaro Facultative Eleotridae
Eleotris klunzingerii RDR0641 Maropapango Facultative Eleotridae
Eleotris klunzingerii RDR0832 Mananjeba West RN6 Facultative Eleotridae
Eleotris klunzingerii RDR0991 Anjambazamba Facultative Eleotridae
Eleotris sp MN045263 Madagascar Freshwater Eleotridae
Eleotris sp MN045265 Madagascar Freshwater Eleotridae
Eleotris sp MN045264 Madagascar Freshwater Eleotridae
Eleotris cf melanosoma FGZC997 north Antsiranana Bach unterhalb Kings Lodge an Stasse Facultative Eleotridae
Giuris cf margaritacea ZCMV441 Manombo village swamp Facultative Eleotridae
Giuris cf margaritacea RDR0012 Madagascar Facultative Eleotridae
Ratsirakia legendrei RDR1070 Tetezamaitso Freshwater Eleotridae
Ratsirakia legendrei RDR1069 Tetezamaitso Freshwater Eleotridae
Ratsirakia legendrei RDR1071 Tetezamaitso Freshwater Eleotridae
Ratsirakia legendrei RDR1072 Tetezamaitso Freshwater Eleotridae
Ratsirakia legendrei RDR1073 Tetezamaitso Freshwater Eleotridae
Ratsirakia legendrei RDR0263 Tetezamby Freshwater Eleotridae
Ratsirakia legendrei RDR0265 Tetezamby Freshwater Eleotridae
Ratsirakia legendrei RDR0262 Tetezamby Freshwater Eleotridae
Ratsirakia legendrei RDR0260 Tetezamby Freshwater Eleotridae
Ratsirakia legendrei RDR0291 Andramikely Freshwater Eleotridae
Ratsirakia legendrei RDR0264 Tetezamby Freshwater Eleotridae
Hypseleotris cf tohizonae ZCMV450 Manombo Camp Freshwater Eleotridae
Hypseleotris cf tohizonae ZCMV14791 Manombo Freshwater Eleotridae
Hypseleotris cf tohizonae ZCMV445 Manombo Camp Freshwater Eleotridae
Hypseleotris cf tohizonae ZCMV439 Manombo village swamp Freshwater Eleotridae
Hypseleotris cf tohizonae ZCMV446 Manombo Camp Freshwater Eleotridae
Hypseleotris cf tohizonae ZCMV449 Manombo Camp Freshwater Eleotridae
Hypseleotris cf tohizonae ZCMV451 Manombo Camp Freshwater Eleotridae
Hypseleotris cf tohizonae RDR0983 Ampanobe Freshwater Eleotridae
Hypseleotris cf tohizonae RDR0972 Sandrikamagnina Freshwater Eleotridae
Hypseleotris cf tohizonae RDR1017 Isahana Freshwater Eleotridae
Hypseleotris cf tohizonae RDR1018 Isahana Freshwater Eleotridae
Hypseleotris cf tohizonae RDR1024 Atokona Ambohimalaza Freshwater Eleotridae
Hypseleotris cf tohizonae RDR1023 Atokona Ambohimalaza Freshwater Eleotridae
Hypseleotris cf tohizonae ZCMV447 Manombo Camp Freshwater Eleotridae
Hypseleotris cf tohizonae ZCMV448 Manombo Camp Freshwater Eleotridae
Hypseleotris cf tohizonae ZCMV443 Manombo Camp Freshwater Eleotridae
Hypseleotris cf tohizonae ZCMV14790 Manombo Freshwater Eleotridae
Hypseleotris cf tohizonae ZCMV442 Manombo village swamp Freshwater Eleotridae
Hypseleotris cf tohizonae ZCMV444 Manombo Camp Freshwater Eleotridae
Hypseleotris cf tohizonae ZCMV440 Manombo village swamp Freshwater Eleotridae
Awaous aeneofuscus RDR1121 Anandray Facultative Gobiidae
Awaous aeneofuscus RDR0678 Ankaramihely Facultative Gobiidae
Sicyopterus punctissimus MK496975 Madagascar Facultative Gobiidae

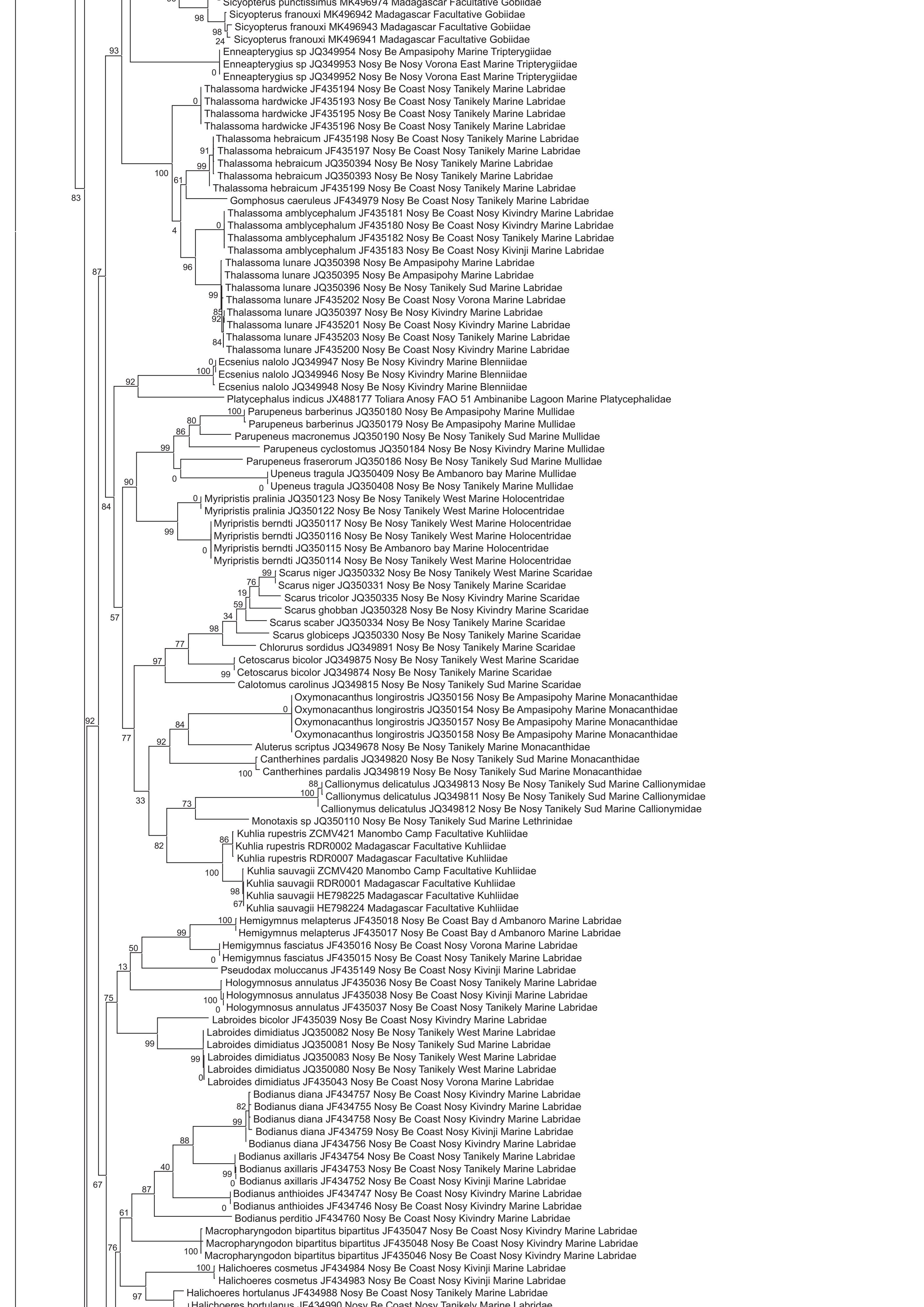
Sicyopterus punctissimus MK496974 Madagascar Facultative Gobiidae
Sicyopterus franouxi MK496942 Madagascar Facultative Gobiidae
Sicyopterus franouxi MK496943 Madagascar Facultative Gobiidae
Sicyopterus franouxi MK496941 Madagascar Facultative Gobiidae
Enneapterygius sp JQ349954 Nosy Be Ampasipohy Marine Tripterygiidae
Enneapterygius sp JQ349953 Nosy Be Nosy Vorona East Marine Tripterygiidae
Enneapterygius sp JQ349952 Nosy Be Nosy Vorona East Marine Tripterygiidae
Thalassoma hardwicke JF435194 Nosy Be Coast Nosy Tanikely Marine Labridae
Thalassoma hardwicke JF435193 Nosy Be Coast Nosy Tanikely Marine Labridae
Thalassoma hardwicke JF435195 Nosy Be Coast Nosy Tanikely Marine Labridae
Thalassoma hardwicke JF435196 Nosy Be Coast Nosy Tanikely Marine Labridae
Thalassoma hebraicum JF435198 Nosy Be Coast Nosy Tanikely Marine Labridae
Thalassoma hebraicum JF435197 Nosy Be Coast Nosy Tanikely Marine Labridae
Thalassoma hebraicum JQ350394 Nosy Be Nosy Tanikely Marine Labridae
Thalassoma hebraicum JQ350393 Nosy Be Nosy Tanikely Marine Labridae
Thalassoma hebraicum JF435199 Nosy Be Coast Nosy Tanikely Marine Labridae
Gomphosus caeruleus JF434979 Nosy Be Coast Nosy Tanikely Marine Labridae
Thalassoma amblycephalum JF435181 Nosy Be Coast Nosy Kivindry Marine Labridae
Thalassoma amblycephalum JF435180 Nosy Be Coast Nosy Kivindry Marine Labridae
Thalassoma amblycephalum JF435182 Nosy Be Coast Nosy Tanikely Marine Labridae
Thalassoma amblycephalum JF435183 Nosy Be Coast Nosy Kivinji Marine Labridae
Thalassoma lunare JQ350398 Nosy Be Ampasipohy Marine Labridae
Thalassoma lunare JQ350395 Nosy Be Ampasipohy Marine Labridae
Thalassoma lunare JQ350396 Nosy Be Nosy Tanikely Sud Marine Labridae
Thalassoma lunare JF435202 Nosy Be Coast Nosy Vorona Marine Labridae
Thalassoma lunare JQ350397 Nosy Be Nosy Kivindry Marine Labridae
Thalassoma lunare JF435201 Nosy Be Coast Nosy Kivindry Marine Labridae
Thalassoma lunare JF435203 Nosy Be Coast Nosy Tanikely Marine Labridae
Thalassoma lunare JF435200 Nosy Be Coast Nosy Kivindry Marine Labridae
Ecsenius nalolo JQ349947 Nosy Be Nosy Kivindry Marine Blenniidae
Ecsenius nalolo JQ349946 Nosy Be Nosy Kivindry Marine Blenniidae
Ecsenius nalolo JQ349948 Nosy Be Nosy Kivindry Marine Blenniidae
Platycephalus indicus JX488177 Toliara Anosy FAO 51 Ambinanibe Lagoon Marine Platycephalidae
Parupeneus barberinus JQ350180 Nosy Be Ampasipohy Marine Mullidae
Parupeneus barberinus JQ350179 Nosy Be Ampasipohy Marine Mullidae
Parupeneus macronemus JQ350190 Nosy Be Nosy Tanikely Sud Marine Mullidae
Parupeneus cyclostomus JQ350184 Nosy Be Nosy Kivindry Marine Mullidae
Parupeneus fraserorum JQ350186 Nosy Be Nosy Tanikely Sud Marine Mullidae
Upeneus tragula JQ350409 Nosy Be Ambanoro bay Marine Mullidae
Upeneus tragula JQ350408 Nosy Be Nosy Tanikely Marine Mullidae
Myripristis pralinia JQ350123 Nosy Be Nosy Tanikely West Marine Holocentridae
Myripristis pralinia JQ350122 Nosy Be Nosy Tanikely West Marine Holocentridae
Myripristis berndti JQ350117 Nosy Be Nosy Tanikely West Marine Holocentridae
Myripristis berndti JQ350116 Nosy Be Nosy Tanikely West Marine Holocentridae
Myripristis berndti JQ350115 Nosy Be Ambanoro bay Marine Holocentridae
Myripristis berndti JQ350114 Nosy Be Nosy Tanikely West Marine Holocentridae
Scarus niger JQ350332 Nosy Be Nosy Tanikely West Marine Scaridae
Scarus niger JQ350331 Nosy Be Nosy Tanikely Marine Scaridae
Scarus tricolor JQ350335 Nosy Be Nosy Kivindry Marine Scaridae
Scarus ghobban JQ350328 Nosy Be Nosy Kivindry Marine Scaridae
Scarus scaber JQ350334 Nosy Be Nosy Tanikely Marine Scaridae
Scarus globiceps JQ350330 Nosy Be Nosy Tanikely Marine Scaridae
Chlorurus sordidus JQ349891 Nosy Be Nosy Tanikely Marine Scaridae
Cetoscarus bicolor JQ349875 Nosy Be Nosy Tanikely West Marine Scaridae
Cetoscarus bicolor JQ349874 Nosy Be Nosy Tanikely Marine Scaridae
Calotomus carolinus JQ349815 Nosy Be Nosy Tanikely Sud Marine Scaridae
Oxymonacanthus longirostris JQ350156 Nosy Be Ampasipohy Marine Monacanthidae
Oxymonacanthus longirostris JQ350154 Nosy Be Ampasipohy Marine Monacanthidae
Oxymonacanthus longirostris JQ350157 Nosy Be Ampasipohy Marine Monacanthidae
Oxymonacanthus longirostris JQ350158 Nosy Be Ampasipohy Marine Monacanthidae
Aluterus scriptus JQ349678 Nosy Be Nosy Tanikely Marine Monacanthidae
Cantherhines pardalis JQ349820 Nosy Be Nosy Tanikely Sud Marine Monacanthidae
Cantherhines pardalis JQ349819 Nosy Be Nosy Tanikely Sud Marine Monacanthidae
Callionymus delicatulus JQ349813 Nosy Be Nosy Tanikely Sud Marine Callionymidae
Callionymus delicatulus JQ349811 Nosy Be Nosy Tanikely Sud Marine Callionymidae
Callionymus delicatulus JQ349812 Nosy Be Nosy Tanikely Sud Marine Callionymidae
Monotaxis sp JQ350110 Nosy Be Nosy Tanikely Sud Marine Lethrinidae
Kuhlia rupestris ZCMV421 Manombo Camp Facultative Kuhliidae
Kuhlia rupestris RDR0002 Madagascar Facultative Kuhliidae
Kuhlia rupestris RDR0007 Madagascar Facultative Kuhliidae
Kuhlia sauvagii ZCMV420 Manombo Camp Facultative Kuhliidae
Kuhlia sauvagii RDR0001 Madagascar Facultative Kuhliidae
Kuhlia sauvagii HE798225 Madagascar Facultative Kuhliidae
Kuhlia sauvagii HE798224 Madagascar Facultative Kuhliidae
Hemigymnus melapterus JF435018 Nosy Be Coast Bay d Ambanoro Marine Labridae
Hemigymnus melapterus JF435017 Nosy Be Coast Bay d Ambanoro Marine Labridae
Hemigymnus fasciatus JF435016 Nosy Be Coast Nosy Vorona Marine Labridae
Hemigymnus fasciatus JF435015 Nosy Be Coast Nosy Tanikely Marine Labridae
Pseudodax moluccanus JF435149 Nosy Be Coast Nosy Kivinji Marine Labridae
Hologymnosus annulatus JF435036 Nosy Be Coast Nosy Tanikely Marine Labridae
Hologymnosus annulatus JF435038 Nosy Be Coast Nosy Kivinji Marine Labridae
Hologymnosus annulatus JF435037 Nosy Be Coast Nosy Tanikely Marine Labridae
Labroides bicolor JF435039 Nosy Be Coast Nosy Kivindry Marine Labridae
Labroides dimidiatus JQ350082 Nosy Be Nosy Tanikely West Marine Labridae
Labroides dimidiatus JQ350081 Nosy Be Nosy Tanikely Sud Marine Labridae
Labroides dimidiatus JQ350083 Nosy Be Nosy Tanikely West Marine Labridae
Labroides dimidiatus JQ350080 Nosy Be Nosy Tanikely West Marine Labridae
Labroides dimidiatus JF435043 Nosy Be Coast Nosy Vorona Marine Labridae
Bodianus diana JF434757 Nosy Be Coast Nosy Kivindry Marine Labridae
Bodianus diana JF434755 Nosy Be Coast Nosy Kivindry Marine Labridae
Bodianus diana JF434758 Nosy Be Coast Nosy Kivindry Marine Labridae
Bodianus diana JF434759 Nosy Be Coast Nosy Kivinji Marine Labridae
Bodianus diana JF434756 Nosy Be Coast Nosy Kivindry Marine Labridae
Bodianus axillaris JF434754 Nosy Be Coast Nosy Tanikely Marine Labridae
Bodianus axillaris JF434753 Nosy Be Coast Nosy Tanikely Marine Labridae
Bodianus axillaris JF434752 Nosy Be Coast Nosy Kivinji Marine Labridae
Bodianus anthioides JF434747 Nosy Be Coast Nosy Kivindry Marine Labridae
Bodianus anthioides JF434746 Nosy Be Coast Nosy Kivindry Marine Labridae
Bodianus perditio JF434760 Nosy Be Coast Nosy Kivindry Marine Labridae
Macropharyngodon bipartitus bipartitus JF435047 Nosy Be Coast Nosy Kivindry Marine Labridae
Macropharyngodon bipartitus bipartitus JF435048 Nosy Be Coast Nosy Kivindry Marine Labridae
Macropharyngodon bipartitus bipartitus JF435046 Nosy Be Coast Nosy Kivindry Marine Labridae
Halichoeres cosmetus JF434984 Nosy Be Coast Nosy Kivinji Marine Labridae
Halichoeres cosmetus JF434983 Nosy Be Coast Nosy Kivinji Marine Labridae
Halichoeres hortulanus JF434988 Nosy Be Coast Nosy Tanikely Marine Labridae
Halichoeres hortulanus JF434990 Nosy Be Coast Nosy Tanikely Marine Labridae

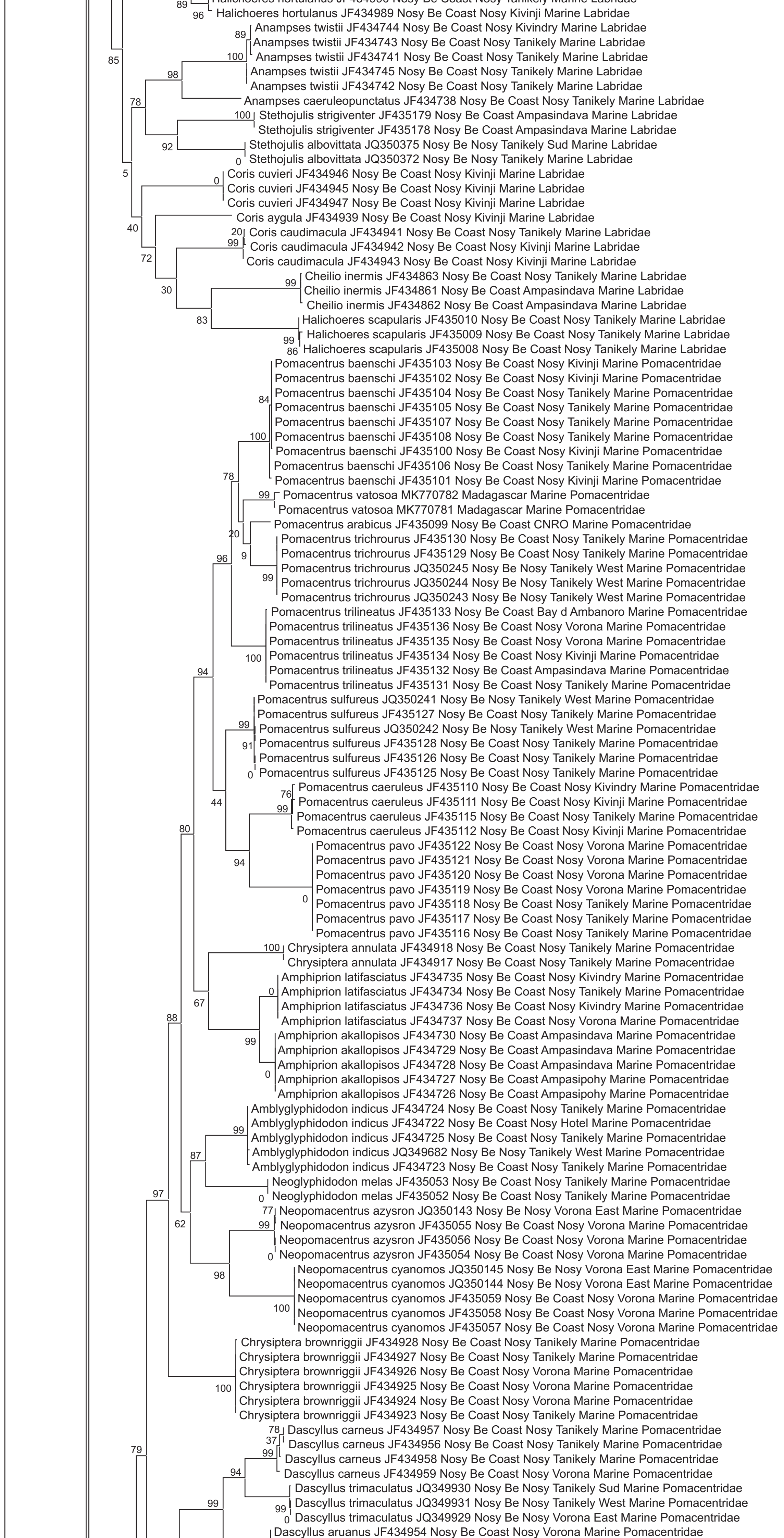
Halichoeres hortulanus JF434989 Nosy Be Coast Nosy Kivinji Marine Labridae
Anampses twistii JF434744 Nosy Be Coast Nosy Kivindry Marine Labridae
Anampses twistii JF434743 Nosy Be Coast Nosy Tanikely Marine Labridae
Anampses twistii JF434741 Nosy Be Coast Nosy Tanikely Marine Labridae
Anampses twistii JF434745 Nosy Be Coast Nosy Tanikely Marine Labridae
Anampses twistii JF434742 Nosy Be Coast Nosy Tanikely Marine Labridae
Anampses caeruleopunctatus JF434738 Nosy Be Coast Nosy Tanikely Marine Labridae
Stethojulis strigiventer JF435179 Nosy Be Coast Ampasindava Marine Labridae
Stethojulis strigiventer JF435178 Nosy Be Coast Ampasindava Marine Labridae
Stethojulis albovittata JQ350375 Nosy Be Nosy Tanikely Sud Marine Labridae
Stethojulis albovittata JQ350372 Nosy Be Nosy Tanikely Marine Labridae
Coris cuvieri JF434946 Nosy Be Coast Nosy Kivinji Marine Labridae
Coris cuvieri JF434945 Nosy Be Coast Nosy Kivinji Marine Labridae
Coris cuvieri JF434947 Nosy Be Coast Nosy Kivinji Marine Labridae
Coris aygula JF434939 Nosy Be Coast Nosy Kivinji Marine Labridae
Coris caudimacula JF434941 Nosy Be Coast Nosy Tanikely Marine Labridae
Coris caudimacula JF434942 Nosy Be Coast Nosy Kivinji Marine Labridae
Coris caudimacula JF434943 Nosy Be Coast Nosy Kivinji Marine Labridae
Cheilio inermis JF434863 Nosy Be Coast Nosy Tanikely Marine Labridae
Cheilio inermis JF434861 Nosy Be Coast Ampasindava Marine Labridae
Cheilio inermis JF434862 Nosy Be Coast Ampasindava Marine Labridae
Halichoeres scapularis JF435010 Nosy Be Coast Nosy Tanikely Marine Labridae
Halichoeres scapularis JF435009 Nosy Be Coast Nosy Tanikely Marine Labridae
Halichoeres scapularis JF435008 Nosy Be Coast Nosy Tanikely Marine Labridae
Pomacentrus baenschi JF435103 Nosy Be Coast Nosy Kivinji Marine Pomacentridae
Pomacentrus baenschi JF435102 Nosy Be Coast Nosy Kivinji Marine Pomacentridae
Pomacentrus baenschi JF435104 Nosy Be Coast Nosy Tanikely Marine Pomacentridae
Pomacentrus baenschi JF435105 Nosy Be Coast Nosy Tanikely Marine Pomacentridae
Pomacentrus baenschi JF435107 Nosy Be Coast Nosy Tanikely Marine Pomacentridae
Pomacentrus baenschi JF435108 Nosy Be Coast Nosy Tanikely Marine Pomacentridae
Pomacentrus baenschi JF435100 Nosy Be Coast Nosy Kivinji Marine Pomacentridae
Pomacentrus baenschi JF435106 Nosy Be Coast Nosy Tanikely Marine Pomacentridae
Pomacentrus baenschi JF435101 Nosy Be Coast Nosy Kivinji Marine Pomacentridae
Pomacentrus vatosoa MK770782 Madagascar Marine Pomacentridae
Pomacentrus vatosoa MK770781 Madagascar Marine Pomacentridae
Pomacentrus arabicus JF435099 Nosy Be Coast CNRO Marine Pomacentridae
Pomacentrus trichrourus JF435130 Nosy Be Coast Nosy Tanikely Marine Pomacentridae
Pomacentrus trichrourus JF435129 Nosy Be Coast Nosy Tanikely Marine Pomacentridae
Pomacentrus trichrourus JQ350245 Nosy Be Nosy Tanikely West Marine Pomacentridae
Pomacentrus trichrourus JQ350244 Nosy Be Nosy Tanikely West Marine Pomacentridae
Pomacentrus trichrourus JQ350243 Nosy Be Nosy Tanikely West Marine Pomacentridae
Pomacentrus trilineatus JF435133 Nosy Be Coast Bay d Ambanoro Marine Pomacentridae
Pomacentrus trilineatus JF435136 Nosy Be Coast Nosy Vorona Marine Pomacentridae
Pomacentrus trilineatus JF435135 Nosy Be Coast Nosy Vorona Marine Pomacentridae
Pomacentrus trilineatus JF435134 Nosy Be Coast Nosy Kivinji Marine Pomacentridae
Pomacentrus trilineatus JF435132 Nosy Be Coast Ampasindava Marine Pomacentridae
Pomacentrus trilineatus JF435131 Nosy Be Coast Nosy Tanikely Marine Pomacentridae
Pomacentrus sulfureus JQ350241 Nosy Be Nosy Tanikely West Marine Pomacentridae
Pomacentrus sulfureus JF435127 Nosy Be Coast Nosy Tanikely Marine Pomacentridae
Pomacentrus sulfureus JQ350242 Nosy Be Nosy Tanikely West Marine Pomacentridae
Pomacentrus sulfureus JF435128 Nosy Be Coast Nosy Tanikely Marine Pomacentridae
Pomacentrus sulfureus JF435126 Nosy Be Coast Nosy Tanikely Marine Pomacentridae
Pomacentrus sulfureus JF435125 Nosy Be Coast Nosy Tanikely Marine Pomacentridae
Pomacentrus caeruleus JF435110 Nosy Be Coast Nosy Kivindry Marine Pomacentridae
Pomacentrus caeruleus JF435111 Nosy Be Coast Nosy Kivinji Marine Pomacentridae
Pomacentrus caeruleus JF435115 Nosy Be Coast Nosy Tanikely Marine Pomacentridae
Pomacentrus caeruleus JF435112 Nosy Be Coast Nosy Kivinji Marine Pomacentridae
Pomacentrus pavo JF435122 Nosy Be Coast Nosy Vorona Marine Pomacentridae
Pomacentrus pavo JF435121 Nosy Be Coast Nosy Vorona Marine Pomacentridae
Pomacentrus pavo JF435120 Nosy Be Coast Nosy Vorona Marine Pomacentridae
Pomacentrus pavo JF435119 Nosy Be Coast Nosy Vorona Marine Pomacentridae
Pomacentrus pavo JF435118 Nosy Be Coast Nosy Tanikely Marine Pomacentridae
Pomacentrus pavo JF435117 Nosy Be Coast Nosy Tanikely Marine Pomacentridae
Pomacentrus pavo JF435116 Nosy Be Coast Nosy Tanikely Marine Pomacentridae
Chrysiptera annulata JF434918 Nosy Be Coast Nosy Tanikely Marine Pomacentridae
Chrysiptera annulata JF434917 Nosy Be Coast Nosy Tanikely Marine Pomacentridae
Amphiprion latifasciatus JF434735 Nosy Be Coast Nosy Kivindry Marine Pomacentridae
Amphiprion latifasciatus JF434734 Nosy Be Coast Nosy Tanikely Marine Pomacentridae
Amphiprion latifasciatus JF434736 Nosy Be Coast Nosy Kivindry Marine Pomacentridae
Amphiprion latifasciatus JF434737 Nosy Be Coast Nosy Vorona Marine Pomacentridae
Amphiprion akallopisos JF434730 Nosy Be Coast Ampasindava Marine Pomacentridae
Amphiprion akallopisos JF434729 Nosy Be Coast Ampasindava Marine Pomacentridae
Amphiprion akallopisos JF434728 Nosy Be Coast Ampasindava Marine Pomacentridae
Amphiprion akallopisos JF434727 Nosy Be Coast Ampasipohy Marine Pomacentridae
Amphiprion akallopisos JF434726 Nosy Be Coast Ampasipohy Marine Pomacentridae
Amblyglyphidodon indicus JF434724 Nosy Be Coast Nosy Tanikely Marine Pomacentridae
Amblyglyphidodon indicus JF434722 Nosy Be Coast Nosy Hotel Marine Pomacentridae
Amblyglyphidodon indicus JF434725 Nosy Be Coast Nosy Tanikely Marine Pomacentridae
Amblyglyphidodon indicus JQ349682 Nosy Be Nosy Tanikely West Marine Pomacentridae
Amblyglyphidodon indicus JF434723 Nosy Be Coast Nosy Tanikely Marine Pomacentridae
Neoglyphidodon melas JF435053 Nosy Be Coast Nosy Tanikely Marine Pomacentridae
Neoglyphidodon melas JF435052 Nosy Be Coast Nosy Tanikely Marine Pomacentridae
Neopomacentrus azysron JQ350143 Nosy Be Nosy Vorona East Marine Pomacentridae
Neopomacentrus azysron JF435055 Nosy Be Coast Nosy Vorona Marine Pomacentridae
Neopomacentrus azysron JF435056 Nosy Be Coast Nosy Vorona Marine Pomacentridae
Neopomacentrus azysron JF435054 Nosy Be Coast Nosy Vorona Marine Pomacentridae
Neopomacentrus cyanomos JQ350145 Nosy Be Nosy Vorona East Marine Pomacentridae
Neopomacentrus cyanomos JQ350144 Nosy Be Nosy Vorona East Marine Pomacentridae
Neopomacentrus cyanomos JF435059 Nosy Be Coast Nosy Vorona Marine Pomacentridae
Neopomacentrus cyanomos JF435058 Nosy Be Coast Nosy Vorona Marine Pomacentridae
Neopomacentrus cyanomos JF435057 Nosy Be Coast Nosy Vorona Marine Pomacentridae
Chrysiptera brownriggii JF434928 Nosy Be Coast Nosy Tanikely Marine Pomacentridae
Chrysiptera brownriggii JF434927 Nosy Be Coast Nosy Tanikely Marine Pomacentridae
Chrysiptera brownriggii JF434926 Nosy Be Coast Nosy Vorona Marine Pomacentridae
Chrysiptera brownriggii JF434925 Nosy Be Coast Nosy Vorona Marine Pomacentridae
Chrysiptera brownriggii JF434924 Nosy Be Coast Nosy Vorona Marine Pomacentridae
Chrysiptera brownriggii JF434923 Nosy Be Coast Nosy Tanikely Marine Pomacentridae
Dascyllus carneus JF434957 Nosy Be Coast Nosy Tanikely Marine Pomacentridae
Dascyllus carneus JF434956 Nosy Be Coast Nosy Tanikely Marine Pomacentridae
Dascyllus carneus JF434958 Nosy Be Coast Nosy Tanikely Marine Pomacentridae
Dascyllus carneus JF434959 Nosy Be Coast Nosy Vorona Marine Pomacentridae
Dascyllus trimaculatus JQ349930 Nosy Be Nosy Tanikely Sud Marine Pomacentridae
Dascyllus trimaculatus JQ349931 Nosy Be Nosy Tanikely West Marine Pomacentridae
Dascyllus trimaculatus JQ349929 Nosy Be Nosy Vorona East Marine Pomacentridae
Dascyllus aruanus JF434954 Nosy Be Coast Nosy Vorona Marine Pomacentridae

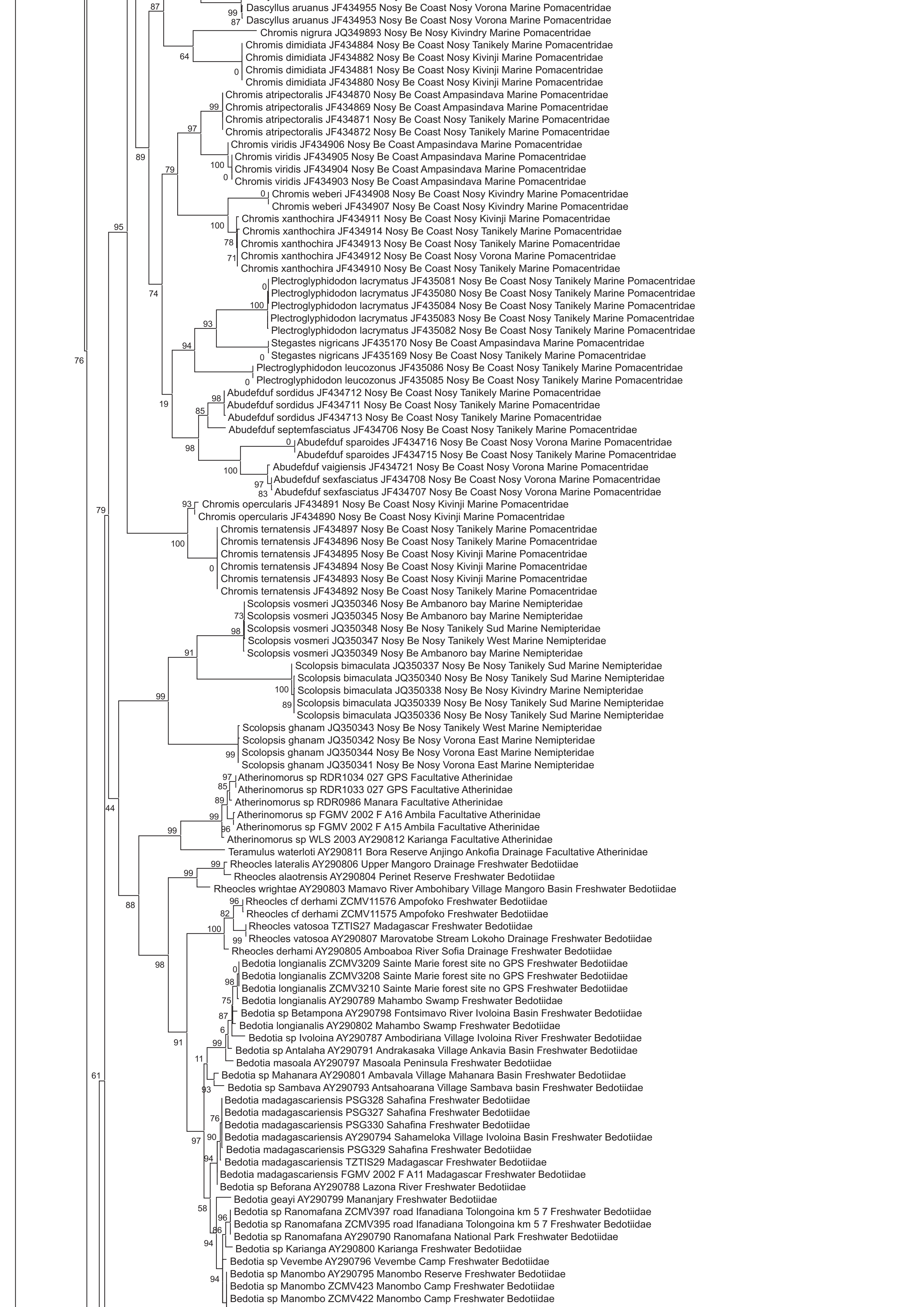
Dascyllus aruanus JF434955 Nosy Be Coast Nosy Vorona Marine Pomacentridae
Dascyllus aruanus JF434953 Nosy Be Coast Nosy Vorona Marine Pomacentridae
Chromis nigrura JQ349893 Nosy Be Nosy Kivindry Marine Pomacentridae
Chromis dimidiata JF434884 Nosy Be Coast Nosy Tanikely Marine Pomacentridae
Chromis dimidiata JF434882 Nosy Be Coast Nosy Kivinji Marine Pomacentridae
Chromis dimidiata JF434881 Nosy Be Coast Nosy Kivinji Marine Pomacentridae
Chromis dimidiata JF434880 Nosy Be Coast Nosy Kivinji Marine Pomacentridae
Chromis atripectoralis JF434870 Nosy Be Coast Ampasindava Marine Pomacentridae
Chromis atripectoralis JF434869 Nosy Be Coast Ampasindava Marine Pomacentridae
Chromis atripectoralis JF434871 Nosy Be Coast Nosy Tanikely Marine Pomacentridae
Chromis atripectoralis JF434872 Nosy Be Coast Nosy Tanikely Marine Pomacentridae
Chromis viridis JF434906 Nosy Be Coast Ampasindava Marine Pomacentridae
Chromis viridis JF434905 Nosy Be Coast Ampasindava Marine Pomacentridae
Chromis viridis JF434904 Nosy Be Coast Ampasindava Marine Pomacentridae
Chromis viridis JF434903 Nosy Be Coast Ampasindava Marine Pomacentridae
Chromis weberi JF434908 Nosy Be Coast Nosy Kivindry Marine Pomacentridae
Chromis weberi JF434907 Nosy Be Coast Nosy Kivindry Marine Pomacentridae
Chromis xanthochira JF434911 Nosy Be Coast Nosy Kivinji Marine Pomacentridae
Chromis xanthochira JF434914 Nosy Be Coast Nosy Tanikely Marine Pomacentridae
Chromis xanthochira JF434913 Nosy Be Coast Nosy Tanikely Marine Pomacentridae
Chromis xanthochira JF434912 Nosy Be Coast Nosy Vorona Marine Pomacentridae
Chromis xanthochira JF434910 Nosy Be Coast Nosy Tanikely Marine Pomacentridae
Plectroglyphidodon lacrymatus JF435081 Nosy Be Coast Nosy Tanikely Marine Pomacentridae
Plectroglyphidodon lacrymatus JF435080 Nosy Be Coast Nosy Tanikely Marine Pomacentridae
Plectroglyphidodon lacrymatus JF435084 Nosy Be Coast Nosy Tanikely Marine Pomacentridae
Plectroglyphidodon lacrymatus JF435083 Nosy Be Coast Nosy Tanikely Marine Pomacentridae
Plectroglyphidodon lacrymatus JF435082 Nosy Be Coast Nosy Tanikely Marine Pomacentridae
Stegastes nigricans JF435170 Nosy Be Coast Ampasindava Marine Pomacentridae
Stegastes nigricans JF435169 Nosy Be Coast Nosy Tanikely Marine Pomacentridae
Plectroglyphidodon leucozonus JF435086 Nosy Be Coast Nosy Tanikely Marine Pomacentridae
Plectroglyphidodon leucozonus JF435085 Nosy Be Coast Nosy Tanikely Marine Pomacentridae
Abudefduf sordidus JF434712 Nosy Be Coast Nosy Tanikely Marine Pomacentridae
Abudefduf sordidus JF434711 Nosy Be Coast Nosy Tanikely Marine Pomacentridae
Abudefduf sordidus JF434713 Nosy Be Coast Nosy Tanikely Marine Pomacentridae
Abudefduf septemfasciatus JF434706 Nosy Be Coast Nosy Tanikely Marine Pomacentridae
Abudefduf sparoides JF434716 Nosy Be Coast Nosy Vorona Marine Pomacentridae
Abudefduf sparoides JF434715 Nosy Be Coast Nosy Tanikely Marine Pomacentridae
Abudefduf vaigiensis JF434721 Nosy Be Coast Nosy Vorona Marine Pomacentridae
Abudefduf sexfasciatus JF434708 Nosy Be Coast Nosy Vorona Marine Pomacentridae
Abudefduf sexfasciatus JF434707 Nosy Be Coast Nosy Vorona Marine Pomacentridae
Chromis opercularis JF434891 Nosy Be Coast Nosy Kivinji Marine Pomacentridae
Chromis opercularis JF434890 Nosy Be Coast Nosy Kivinji Marine Pomacentridae
Chromis ternatensis JF434897 Nosy Be Coast Nosy Tanikely Marine Pomacentridae
Chromis ternatensis JF434896 Nosy Be Coast Nosy Tanikely Marine Pomacentridae
Chromis ternatensis JF434895 Nosy Be Coast Nosy Kivinji Marine Pomacentridae
Chromis ternatensis JF434894 Nosy Be Coast Nosy Kivinji Marine Pomacentridae
Chromis ternatensis JF434893 Nosy Be Coast Nosy Kivinji Marine Pomacentridae
Chromis ternatensis JF434892 Nosy Be Coast Nosy Tanikely Marine Pomacentridae
Scolopsis vosmeri JQ350346 Nosy Be Ambanoro bay Marine Nemipteridae
Scolopsis vosmeri JQ350345 Nosy Be Ambanoro bay Marine Nemipteridae
Scolopsis vosmeri JQ350348 Nosy Be Nosy Tanikely Sud Marine Nemipteridae
Scolopsis vosmeri JQ350347 Nosy Be Nosy Tanikely West Marine Nemipteridae
Scolopsis vosmeri JQ350349 Nosy Be Ambanoro bay Marine Nemipteridae
Scolopsis bimaculata JQ350337 Nosy Be Nosy Tanikely Sud Marine Nemipteridae
Scolopsis bimaculata JQ350340 Nosy Be Nosy Tanikely Sud Marine Nemipteridae
Scolopsis bimaculata JQ350338 Nosy Be Nosy Kivindry Marine Nemipteridae
Scolopsis bimaculata JQ350339 Nosy Be Nosy Tanikely Sud Marine Nemipteridae
Scolopsis bimaculata JQ350336 Nosy Be Nosy Tanikely Sud Marine Nemipteridae
Scolopsis ghanam JQ350343 Nosy Be Nosy Tanikely West Marine Nemipteridae
Scolopsis ghanam JQ350342 Nosy Be Nosy Vorona East Marine Nemipteridae
Scolopsis ghanam JQ350344 Nosy Be Nosy Vorona East Marine Nemipteridae
Scolopsis ghanam JQ350341 Nosy Be Nosy Vorona East Marine Nemipteridae
Atherinomorus sp RDR1034 027 GPS Facultative Atherinidae
Atherinomorus sp RDR1033 027 GPS Facultative Atherinidae
Atherinomorus sp RDR0986 Manara Facultative Atherinidae
Atherinomorus sp FGMV 2002 F A16 Ambila Facultative Atherinidae
Atherinomorus sp FGMV 2002 F A15 Ambila Facultative Atherinidae
Atherinomorus sp WLS 2003 AY290812 Karianga Facultative Atherinidae
Teramulus waterloti AY290811 Bora Reserve Anjingo Ankofia Drainage Facultative Atherinidae
Rheocles lateralis AY290806 Upper Mangoro Drainage Freshwater Bedotiidae
Rheocles alaotrensis AY290804 Perinet Reserve Freshwater Bedotiidae
Rheocles wrightae AY290803 Mamavo River Ambohibary Village Mangoro Basin Freshwater Bedotiidae
Rheocles cf derhami ZCMV11576 Ampofoko Freshwater Bedotiidae
Rheocles cf derhami ZCMV11575 Ampofoko Freshwater Bedotiidae
Rheocles vatosoa TZTIS27 Madagascar Freshwater Bedotiidae
Rheocles vatosoa AY290807 Marovatobe Stream Lokoho Drainage Freshwater Bedotiidae
Rheocles derhami AY290805 Amboaboa River Sofia Drainage Freshwater Bedotiidae
Bedotia longianalis ZCMV3209 Sainte Marie forest site no GPS Freshwater Bedotiidae
Bedotia longianalis ZCMV3208 Sainte Marie forest site no GPS Freshwater Bedotiidae
Bedotia longianalis ZCMV3210 Sainte Marie forest site no GPS Freshwater Bedotiidae
Bedotia longianalis AY290789 Mahambo Swamp Freshwater Bedotiidae
Bedotia sp Betampona AY290798 Fontsimavo River Ivoloina Basin Freshwater Bedotiidae
Bedotia longianalis AY290802 Mahambo Swamp Freshwater Bedotiidae
Bedotia sp Ivoloina AY290787 Ambodiriana Village Ivoloina River Freshwater Bedotiidae
Bedotia sp Antalaha AY290791 Andrakasaka Village Ankavia Basin Freshwater Bedotiidae
Bedotia masoala AY290797 Masoala Peninsula Freshwater Bedotiidae
Bedotia sp Mahanara AY290801 Ambavala Village Mahanara Basin Freshwater Bedotiidae
Bedotia sp Sambava AY290793 Antsahoarana Village Sambava basin Freshwater Bedotiidae
Bedotia madagascariensis PSG328 Sahafina Freshwater Bedotiidae
Bedotia madagascariensis PSG327 Sahafina Freshwater Bedotiidae
Bedotia madagascariensis PSG330 Sahafina Freshwater Bedotiidae
Bedotia madagascariensis AY290794 Sahameloka Village Ivoloina Basin Freshwater Bedotiidae
Bedotia madagascariensis PSG329 Sahafina Freshwater Bedotiidae
Bedotia madagascariensis TZTIS29 Madagascar Freshwater Bedotiidae
Bedotia madagascariensis FGMV 2002 F A11 Madagascar Freshwater Bedotiidae
Bedotia sp Beforana AY290788 Lazona River Freshwater Bedotiidae
Bedotia geayi AY290799 Mananjary Freshwater Bedotiidae
Bedotia sp Ranomafana ZCMV397 road Ifanadiana Tolongoina km 5 7 Freshwater Bedotiidae
Bedotia sp Ranomafana ZCMV395 road Ifanadiana Tolongoina km 5 7 Freshwater Bedotiidae
Bedotia sp Ranomafana AY290790 Ranomafana National Park Freshwater Bedotiidae
Bedotia sp Karianga AY290800 Karianga Freshwater Bedotiidae
Bedotia sp Vevembe AY290796 Vevembe Camp Freshwater Bedotiidae
Bedotia sp Manombo AY290795 Manombo Reserve Freshwater Bedotiidae
Bedotia sp Manombo ZCMV423 Manombo Camp Freshwater Bedotiidae
Bedotia sp Manombo ZCMV422 Manombo Camp Freshwater Bedotiidae

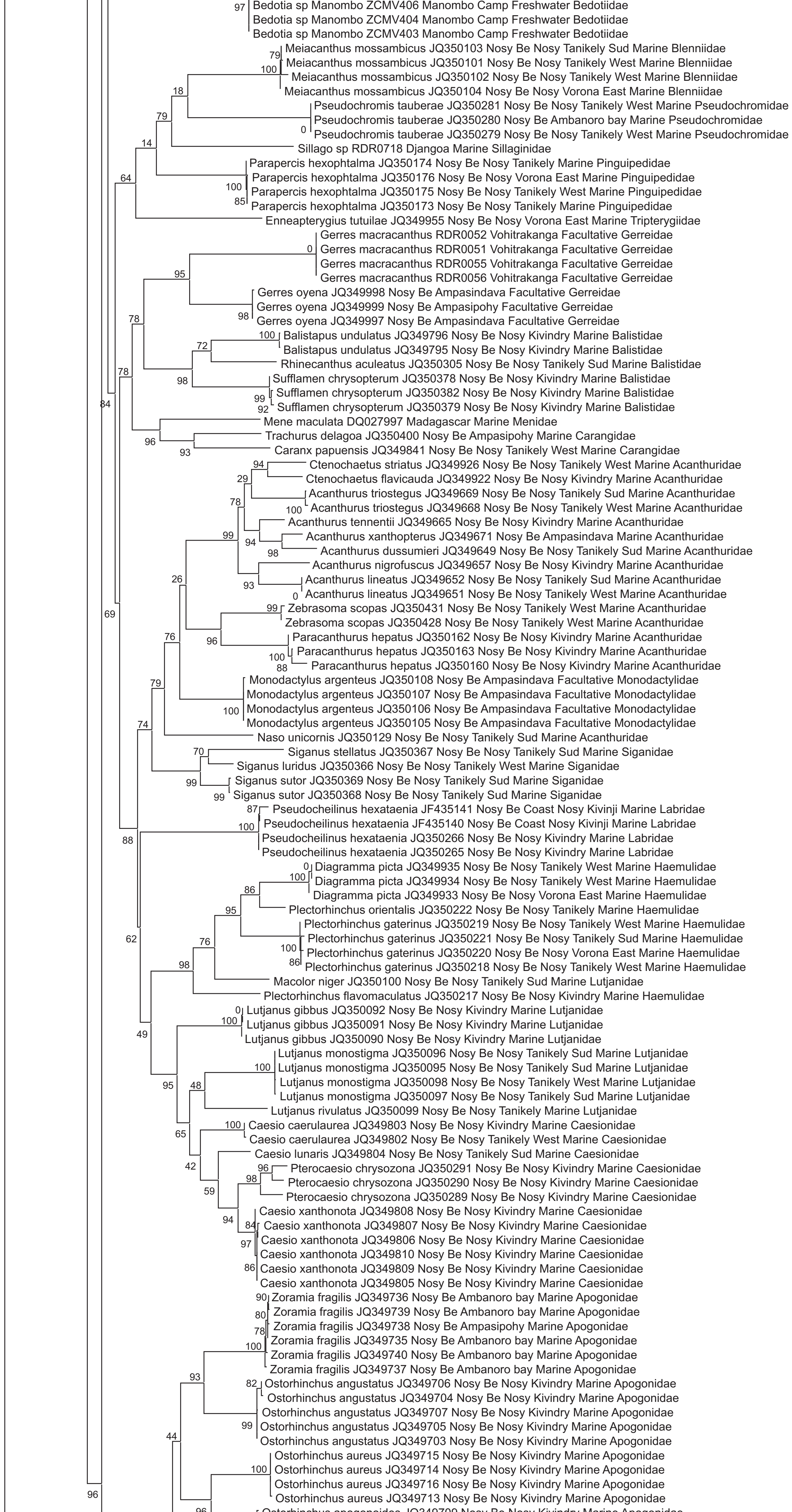

Bedotia sp Manombo ZCMV406 Manombo Camp Freshwater Bedotiidae
Bedotia sp Manombo ZCMV404 Manombo Camp Freshwater Bedotiidae
Bedotia sp Manombo ZCMV403 Manombo Camp Freshwater Bedotiidae
Meiacanthus mossambicus JQ350103 Nosy Be Nosy Tanikely Sud Marine Blenniidae
Meiacanthus mossambicus JQ350101 Nosy Be Nosy Tanikely West Marine Blenniidae
Meiacanthus mossambicus JQ350102 Nosy Be Nosy Tanikely West Marine Blenniidae
Meiacanthus mossambicus JQ350104 Nosy Be Nosy Vorona East Marine Blenniidae
Pseudochromis tauberae JQ350281 Nosy Be Nosy Tanikely West Marine Pseudochromidae
Pseudochromis tauberae JQ350280 Nosy Be Ambanoro bay Marine Pseudochromidae
Pseudochromis tauberae JQ350279 Nosy Be Nosy Tanikely West Marine Pseudochromidae
Sillago sp RDR0718 Djangoa Marine Sillaginidae
Parapercis hexophtalma JQ350174 Nosy Be Nosy Tanikely Marine Pinguipedidae
Parapercis hexophtalma JQ350176 Nosy Be Nosy Vorona East Marine Pinguipedidae
Parapercis hexophtalma JQ350175 Nosy Be Nosy Tanikely West Marine Pinguipedidae
Parapercis hexophtalma JQ350173 Nosy Be Nosy Tanikely Marine Pinguipedidae
Enneapterygius tutuilae JQ349955 Nosy Be Nosy Vorona East Marine Tripterygiidae
Gerres macracanthus RDR0052 Vohitrakanga Facultative Gerreidae
Gerres macracanthus RDR0051 Vohitrakanga Facultative Gerreidae
Gerres macracanthus RDR0055 Vohitrakanga Facultative Gerreidae
Gerres macracanthus RDR0056 Vohitrakanga Facultative Gerreidae
Gerres oyena JQ349998 Nosy Be Ampasindava Facultative Gerreidae
Gerres oyena JQ349999 Nosy Be Ampasipohy Facultative Gerreidae
Gerres oyena JQ349997 Nosy Be Ampasindava Facultative Gerreidae
Balistapus undulatus JQ349796 Nosy Be Nosy Kivindry Marine Balistidae
Balistapus undulatus JQ349795 Nosy Be Nosy Kivindry Marine Balistidae
Rhinecanthus aculeatus JQ350305 Nosy Be Nosy Tanikely Sud Marine Balistidae
Sufflamen chrysopterum JQ350378 Nosy Be Nosy Kivindry Marine Balistidae
Sufflamen chrysopterum JQ350382 Nosy Be Nosy Kivindry Marine Balistidae
Sufflamen chrysopterum JQ350379 Nosy Be Nosy Kivindry Marine Balistidae
Mene maculata DQ027997 Madagascar Marine Menidae
Trachurus delagoa JQ350400 Nosy Be Ampasipohy Marine Carangidae
Caranx papuensis JQ349841 Nosy Be Nosy Tanikely West Marine Carangidae
Ctenochaetus striatus JQ349926 Nosy Be Nosy Tanikely West Marine Acanthuridae
Ctenochaetus flavicauda JQ349922 Nosy Be Nosy Kivindry Marine Acanthuridae
Acanthurus triostegus JQ349669 Nosy Be Nosy Tanikely Sud Marine Acanthuridae
Acanthurus triostegus JQ349668 Nosy Be Nosy Tanikely West Marine Acanthuridae
Acanthurus tennentii JQ349665 Nosy Be Nosy Kivindry Marine Acanthuridae
Acanthurus xanthopterus JQ349671 Nosy Be Ampasindava Marine Acanthuridae
Acanthurus dussumieri JQ349649 Nosy Be Nosy Tanikely Sud Marine Acanthuridae
Acanthurus nigrofuscus JQ349657 Nosy Be Nosy Kivindry Marine Acanthuridae
Acanthurus lineatus JQ349652 Nosy Be Nosy Tanikely Sud Marine Acanthuridae
Acanthurus lineatus JQ349651 Nosy Be Nosy Tanikely West Marine Acanthuridae
Zebrasoma scopas JQ350431 Nosy Be Nosy Tanikely West Marine Acanthuridae
Zebrasoma scopas JQ350428 Nosy Be Nosy Tanikely West Marine Acanthuridae
Paracanthurus hepatus JQ350162 Nosy Be Nosy Kivindry Marine Acanthuridae
Paracanthurus hepatus JQ350163 Nosy Be Nosy Kivindry Marine Acanthuridae
Paracanthurus hepatus JQ350160 Nosy Be Nosy Kivindry Marine Acanthuridae
Monodactylus argenteus JQ350108 Nosy Be Ampasindava Facultative Monodactylidae
Monodactylus argenteus JQ350107 Nosy Be Ampasindava Facultative Monodactylidae
Monodactylus argenteus JQ350106 Nosy Be Ampasindava Facultative Monodactylidae
Monodactylus argenteus JQ350105 Nosy Be Ampasindava Facultative Monodactylidae
Naso unicornis JQ350129 Nosy Be Nosy Tanikely Sud Marine Acanthuridae
Siganus stellatus JQ350367 Nosy Be Nosy Tanikely Sud Marine Siganidae
Siganus luridus JQ350366 Nosy Be Nosy Tanikely West Marine Siganidae
Siganus sutor JQ350369 Nosy Be Nosy Tanikely Sud Marine Siganidae
Siganus sutor JQ350368 Nosy Be Nosy Tanikely Sud Marine Siganidae
Pseudocheilinus hexataenia JF435141 Nosy Be Coast Nosy Kivinji Marine Labridae
Pseudocheilinus hexataenia JF435140 Nosy Be Coast Nosy Kivinji Marine Labridae
Pseudocheilinus hexataenia JQ350266 Nosy Be Nosy Kivindry Marine Labridae
Pseudocheilinus hexataenia JQ350265 Nosy Be Nosy Kivindry Marine Labridae
Diagramma picta JQ349935 Nosy Be Nosy Tanikely West Marine Haemulidae
Diagramma picta JQ349934 Nosy Be Nosy Tanikely West Marine Haemulidae
Diagramma picta JQ349933 Nosy Be Nosy Vorona East Marine Haemulidae
Plectorhinchus orientalis JQ350222 Nosy Be Nosy Tanikely Marine Haemulidae
Plectorhinchus gaterinus JQ350219 Nosy Be Nosy Tanikely West Marine Haemulidae
Plectorhinchus gaterinus JQ350221 Nosy Be Nosy Tanikely Sud Marine Haemulidae
Plectorhinchus gaterinus JQ350220 Nosy Be Nosy Vorona East Marine Haemulidae
Plectorhinchus gaterinus JQ350218 Nosy Be Nosy Tanikely West Marine Haemulidae
Macolor niger JQ350100 Nosy Be Nosy Tanikely Sud Marine Lutjanidae
Plectorhinchus flavomaculatus JQ350217 Nosy Be Nosy Kivindry Marine Haemulidae
Lutjanus gibbus JQ350092 Nosy Be Nosy Kivindry Marine Lutjanidae
Lutjanus gibbus JQ350091 Nosy Be Nosy Kivindry Marine Lutjanidae
Lutjanus gibbus JQ350090 Nosy Be Nosy Kivindry Marine Lutjanidae
Lutjanus monostigma JQ350096 Nosy Be Nosy Tanikely Sud Marine Lutjanidae
Lutjanus monostigma JQ350095 Nosy Be Nosy Tanikely Sud Marine Lutjanidae
Lutjanus monostigma JQ350098 Nosy Be Nosy Tanikely West Marine Lutjanidae
Lutjanus monostigma JQ350097 Nosy Be Nosy Tanikely Sud Marine Lutjanidae
Lutjanus rivulatus JQ350099 Nosy Be Nosy Tanikely Marine Lutjanidae
Caesio caerulaurea JQ349803 Nosy Be Nosy Kivindry Marine Caesionidae
Caesio caerulaurea JQ349802 Nosy Be Nosy Tanikely West Marine Caesionidae
Caesio lunaris JQ349804 Nosy Be Nosy Tanikely Sud Marine Caesionidae
Pterocaesio chrysozona JQ350291 Nosy Be Nosy Kivindry Marine Caesionidae
Pterocaesio chrysozona JQ350290 Nosy Be Nosy Kivindry Marine Caesionidae
Pterocaesio chrysozona JQ350289 Nosy Be Nosy Kivindry Marine Caesionidae
Caesio xanthonota JQ349808 Nosy Be Nosy Kivindry Marine Caesionidae
Caesio xanthonota JQ349807 Nosy Be Nosy Kivindry Marine Caesionidae
Caesio xanthonota JQ349806 Nosy Be Nosy Kivindry Marine Caesionidae
Caesio xanthonota JQ349810 Nosy Be Nosy Kivindry Marine Caesionidae
Caesio xanthonota JQ349809 Nosy Be Nosy Kivindry Marine Caesionidae
Caesio xanthonota JQ349805 Nosy Be Nosy Kivindry Marine Caesionidae
Zoramia fragilis JQ349736 Nosy Be Ambanoro bay Marine Apogonidae
Zoramia fragilis JQ349739 Nosy Be Ambanoro bay Marine Apogonidae
Zoramia fragilis JQ349738 Nosy Be Ampasipohy Marine Apogonidae
Zoramia fragilis JQ349735 Nosy Be Ambanoro bay Marine Apogonidae
Zoramia fragilis JQ349740 Nosy Be Ambanoro bay Marine Apogonidae
Zoramia fragilis JQ349737 Nosy Be Ambanoro bay Marine Apogonidae
Ostorhinchus angustatus JQ349706 Nosy Be Nosy Kivindry Marine Apogonidae
Ostorhinchus angustatus JQ349704 Nosy Be Nosy Kivindry Marine Apogonidae
Ostorhinchus angustatus JQ349707 Nosy Be Nosy Kivindry Marine Apogonidae
Ostorhinchus angustatus JQ349705 Nosy Be Nosy Kivindry Marine Apogonidae
Ostorhinchus angustatus JQ349703 Nosy Be Nosy Kivindry Marine Apogonidae
Ostorhinchus aureus JQ349715 Nosy Be Nosy Kivindry Marine Apogonidae
Ostorhinchus aureus JQ349714 Nosy Be Nosy Kivindry Marine Apogonidae
Ostorhinchus aureus JQ349716 Nosy Be Nosy Kivindry Marine Apogonidae
Ostorhinchus aureus JQ349713 Nosy Be Nosy Kivindry Marine Apogonidae

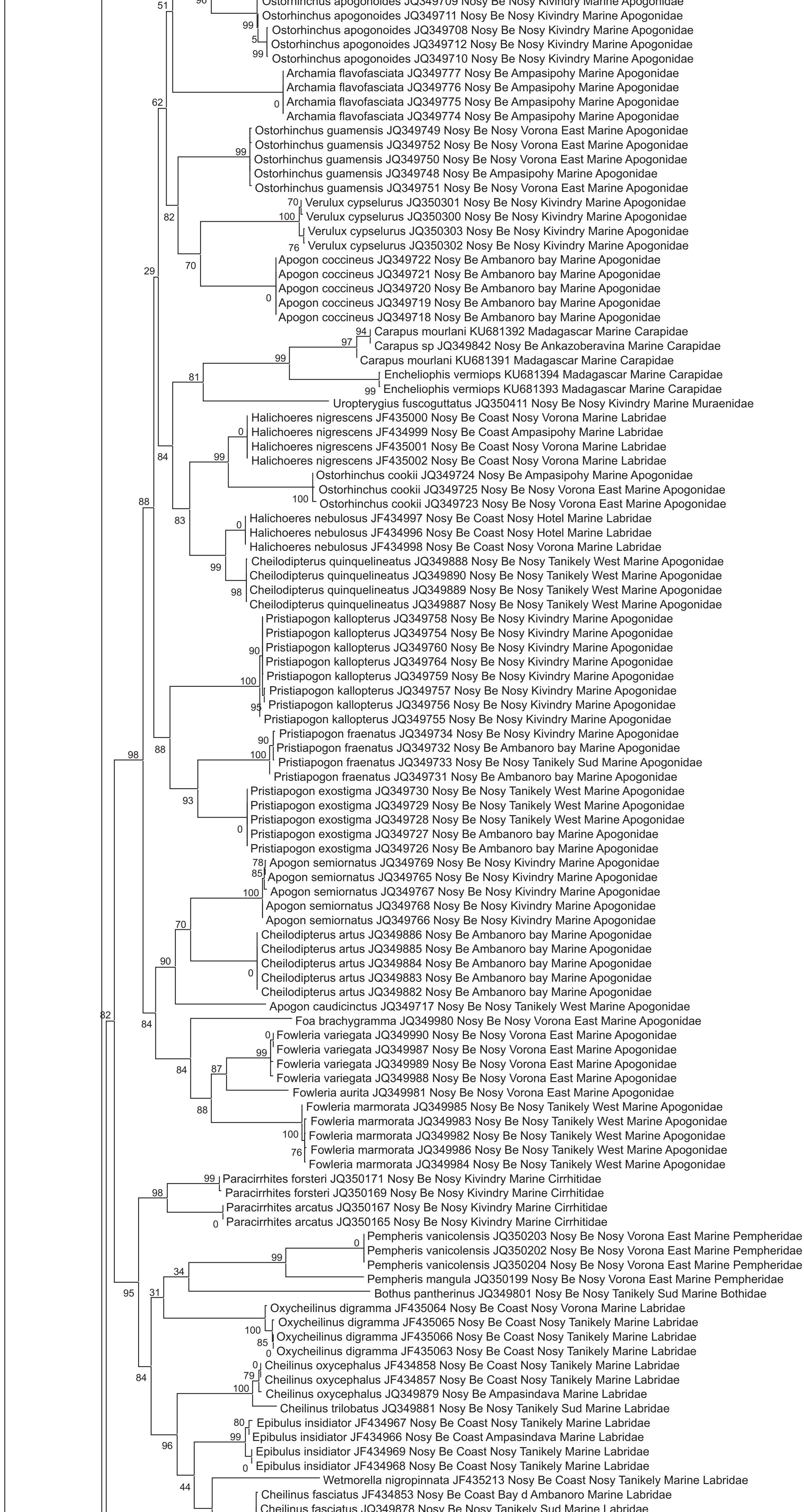
Ostorhinchus apogonoides JQ349709 Nosy Be Nosy Kivindry Marine Apogonidae
Ostorhinchus apogonoides JQ349711 Nosy Be Nosy Kivindry Marine Apogonidae
Ostorhinchus apogonoides JQ349708 Nosy Be Nosy Kivindry Marine Apogonidae
Ostorhinchus apogonoides JQ349712 Nosy Be Nosy Kivindry Marine Apogonidae
Ostorhinchus apogonoides JQ349710 Nosy Be Nosy Kivindry Marine Apogonidae
Archamia flavofasciata JQ349777 Nosy Be Ampasipohy Marine Apogonidae
Archamia flavofasciata JQ349776 Nosy Be Ampasipohy Marine Apogonidae
Archamia flavofasciata JQ349775 Nosy Be Ampasipohy Marine Apogonidae
Archamia flavofasciata JQ349774 Nosy Be Ampasipohy Marine Apogonidae
Ostorhinchus guamensis JQ349749 Nosy Be Nosy Vorona East Marine Apogonidae
Ostorhinchus guamensis JQ349752 Nosy Be Nosy Vorona East Marine Apogonidae
Ostorhinchus guamensis JQ349750 Nosy Be Nosy Vorona East Marine Apogonidae
Ostorhinchus guamensis JQ349748 Nosy Be Ampasipohy Marine Apogonidae
Ostorhinchus guamensis JQ349751 Nosy Be Nosy Vorona East Marine Apogonidae
Verulux cypselurus JQ350301 Nosy Be Nosy Kivindry Marine Apogonidae
Verulux cypselurus JQ350300 Nosy Be Nosy Kivindry Marine Apogonidae
Verulux cypselurus JQ350303 Nosy Be Nosy Kivindry Marine Apogonidae
Verulux cypselurus JQ350302 Nosy Be Nosy Kivindry Marine Apogonidae
Apogon coccineus JQ349722 Nosy Be Ambanoro bay Marine Apogonidae
Apogon coccineus JQ349721 Nosy Be Ambanoro bay Marine Apogonidae
Apogon coccineus JQ349720 Nosy Be Ambanoro bay Marine Apogonidae
Apogon coccineus JQ349719 Nosy Be Ambanoro bay Marine Apogonidae
Apogon coccineus JQ349718 Nosy Be Ambanoro bay Marine Apogonidae
Carapus mourlani KU681392 Madagascar Marine Carapidae
Carapus sp JQ349842 Nosy Be Ankazoberavina Marine Carapidae
Carapus mourlani KU681391 Madagascar Marine Carapidae
Encheliophis vermiops KU681394 Madagascar Marine Carapidae
Encheliophis vermiops KU681393 Madagascar Marine Carapidae
Uropterygius fuscoguttatus JQ350411 Nosy Be Nosy Kivindry Marine Muraenidae
Halichoeres nigrescens JF435000 Nosy Be Coast Nosy Vorona Marine Labridae
Halichoeres nigrescens JF434999 Nosy Be Coast Ampasipohy Marine Labridae
Halichoeres nigrescens JF435001 Nosy Be Coast Nosy Vorona Marine Labridae
Halichoeres nigrescens JF435002 Nosy Be Coast Nosy Vorona Marine Labridae
Ostorhinchus cookii JQ349724 Nosy Be Ampasipohy Marine Apogonidae
Ostorhinchus cookii JQ349725 Nosy Be Nosy Vorona East Marine Apogonidae
Ostorhinchus cookii JQ349723 Nosy Be Nosy Vorona East Marine Apogonidae
Halichoeres nebulosus JF434997 Nosy Be Coast Nosy Hotel Marine Labridae
Halichoeres nebulosus JF434996 Nosy Be Coast Nosy Hotel Marine Labridae
Halichoeres nebulosus JF434998 Nosy Be Coast Nosy Vorona Marine Labridae
Cheilodipterus quinquelineatus JQ349888 Nosy Be Nosy Tanikely West Marine Apogonidae
Cheilodipterus quinquelineatus JQ349890 Nosy Be Nosy Tanikely West Marine Apogonidae
Cheilodipterus quinquelineatus JQ349889 Nosy Be Nosy Tanikely West Marine Apogonidae
Cheilodipterus quinquelineatus JQ349887 Nosy Be Nosy Tanikely West Marine Apogonidae
Pristiapogon kallopterus JQ349758 Nosy Be Nosy Kivindry Marine Apogonidae
Pristiapogon kallopterus JQ349754 Nosy Be Nosy Kivindry Marine Apogonidae
Pristiapogon kallopterus JQ349760 Nosy Be Nosy Kivindry Marine Apogonidae
Pristiapogon kallopterus JQ349764 Nosy Be Nosy Kivindry Marine Apogonidae
Pristiapogon kallopterus JQ349759 Nosy Be Nosy Kivindry Marine Apogonidae
Pristiapogon kallopterus JQ349757 Nosy Be Nosy Kivindry Marine Apogonidae
Pristiapogon kallopterus JQ349756 Nosy Be Nosy Kivindry Marine Apogonidae
Pristiapogon kallopterus JQ349755 Nosy Be Nosy Kivindry Marine Apogonidae
Pristiapogon fraenatus JQ349734 Nosy Be Nosy Kivindry Marine Apogonidae
Pristiapogon fraenatus JQ349732 Nosy Be Ambanoro bay Marine Apogonidae
Pristiapogon fraenatus JQ349733 Nosy Be Nosy Tanikely Sud Marine Apogonidae
Pristiapogon fraenatus JQ349731 Nosy Be Ambanoro bay Marine Apogonidae
Pristiapogon exostigma JQ349730 Nosy Be Nosy Tanikely West Marine Apogonidae
Pristiapogon exostigma JQ349729 Nosy Be Nosy Tanikely West Marine Apogonidae
Pristiapogon exostigma JQ349728 Nosy Be Nosy Tanikely West Marine Apogonidae
Pristiapogon exostigma JQ349727 Nosy Be Ambanoro bay Marine Apogonidae
Pristiapogon exostigma JQ349726 Nosy Be Ambanoro bay Marine Apogonidae
Apogon semiornatus JQ349769 Nosy Be Nosy Kivindry Marine Apogonidae
Apogon semiornatus JQ349765 Nosy Be Nosy Kivindry Marine Apogonidae
Apogon semiornatus JQ349767 Nosy Be Nosy Kivindry Marine Apogonidae
Apogon semiornatus JQ349768 Nosy Be Nosy Kivindry Marine Apogonidae
Apogon semiornatus JQ349766 Nosy Be Nosy Kivindry Marine Apogonidae
Cheilodipterus artus JQ349886 Nosy Be Ambanoro bay Marine Apogonidae
Cheilodipterus artus JQ349885 Nosy Be Ambanoro bay Marine Apogonidae
Cheilodipterus artus JQ349884 Nosy Be Ambanoro bay Marine Apogonidae
Cheilodipterus artus JQ349883 Nosy Be Ambanoro bay Marine Apogonidae
Cheilodipterus artus JQ349882 Nosy Be Ambanoro bay Marine Apogonidae
Apogon caudicinctus JQ349717 Nosy Be Nosy Tanikely West Marine Apogonidae
Foa brachygramma JQ349980 Nosy Be Nosy Vorona East Marine Apogonidae
Fowleria variegata JQ349990 Nosy Be Nosy Vorona East Marine Apogonidae
Fowleria variegata JQ349987 Nosy Be Nosy Vorona East Marine Apogonidae
Fowleria variegata JQ349989 Nosy Be Nosy Vorona East Marine Apogonidae
Fowleria variegata JQ349988 Nosy Be Nosy Vorona East Marine Apogonidae
Fowleria aurita JQ349981 Nosy Be Nosy Vorona East Marine Apogonidae
Fowleria marmorata JQ349985 Nosy Be Nosy Tanikely West Marine Apogonidae
Fowleria marmorata JQ349983 Nosy Be Nosy Tanikely West Marine Apogonidae
Fowleria marmorata JQ349982 Nosy Be Nosy Tanikely West Marine Apogonidae
Fowleria marmorata JQ349986 Nosy Be Nosy Tanikely West Marine Apogonidae
Fowleria marmorata JQ349984 Nosy Be Nosy Tanikely West Marine Apogonidae
Paracirrhites forsteri JQ350171 Nosy Be Nosy Kivindry Marine Cirrhitidae
Paracirrhites forsteri JQ350169 Nosy Be Nosy Kivindry Marine Cirrhitidae
Paracirrhites arcatus JQ350167 Nosy Be Nosy Kivindry Marine Cirrhitidae
Paracirrhites arcatus JQ350165 Nosy Be Nosy Kivindry Marine Cirrhitidae
Pempheris vanicolensis JQ350203 Nosy Be Nosy Vorona East Marine Pempheridae
Pempheris vanicolensis JQ350202 Nosy Be Nosy Vorona East Marine Pempheridae
Pempheris vanicolensis JQ350204 Nosy Be Nosy Vorona East Marine Pempheridae
Pempheris mangula JQ350199 Nosy Be Nosy Vorona East Marine Pempheridae
Bothus pantherinus JQ349801 Nosy Be Nosy Tanikely Sud Marine Bothidae
Oxycheilinus digramma JF435064 Nosy Be Coast Nosy Vorona Marine Labridae
Oxycheilinus digramma JF435065 Nosy Be Coast Nosy Tanikely Marine Labridae
Oxycheilinus digramma JF435066 Nosy Be Coast Nosy Tanikely Marine Labridae
Oxycheilinus digramma JF435063 Nosy Be Coast Nosy Tanikely Marine Labridae
Cheilinus oxycephalus JF434858 Nosy Be Coast Nosy Tanikely Marine Labridae
Cheilinus oxycephalus JF434857 Nosy Be Coast Nosy Tanikely Marine Labridae
Cheilinus oxycephalus JQ349879 Nosy Be Ampasindava Marine Labridae
Cheilinus trilobatus JQ349881 Nosy Be Nosy Tanikely Sud Marine Labridae
Epibulus insidiator JF434967 Nosy Be Coast Nosy Tanikely Marine Labridae
Epibulus insidiator JF434966 Nosy Be Coast Ampasindava Marine Labridae
Epibulus insidiator JF434969 Nosy Be Coast Nosy Tanikely Marine Labridae
Epibulus insidiator JF434968 Nosy Be Coast Nosy Tanikely Marine Labridae
Wetmorella nigropinnata JF435213 Nosy Be Coast Nosy Tanikely Marine Labridae
Cheilinus fasciatus JF434853 Nosy Be Coast Bay d Ambanoro Marine Labridae
Cheilinus fasciatus JQ349878 Nosy Be Nosy Tanikely Sud Marine Labridae

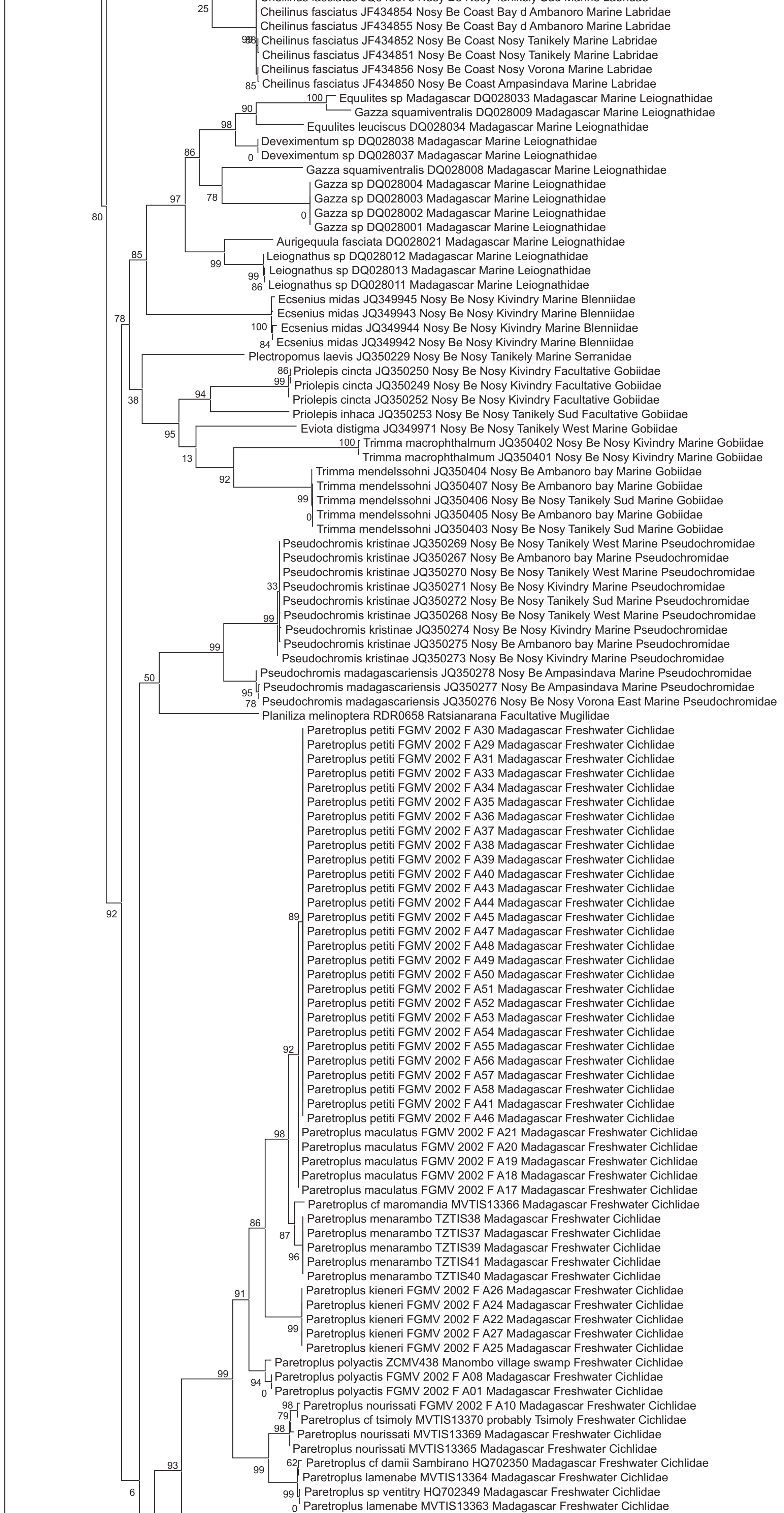
Cheilinus fasciatus JF434854 Nosy Be Coast Bay d Ambanoro Marine Labridae
Cheilinus fasciatus JF434855 Nosy Be Coast Bay d Ambanoro Marine Labridae
Cheilinus fasciatus JF434852 Nosy Be Coast Nosy Tanikely Marine Labridae
Cheilinus fasciatus JF434851 Nosy Be Coast Nosy Tanikely Marine Labridae
Cheilinus fasciatus JF434856 Nosy Be Coast Nosy Vorona Marine Labridae
Cheilinus fasciatus JF434850 Nosy Be Coast Ampasindava Marine Labridae
Equulites sp Madagascar DQ028033 Madagascar Marine Leiognathidae
Gazza squamiventralis DQ028009 Madagascar Marine Leiognathidae
Equulites leuciscus DQ028034 Madagascar Marine Leiognathidae
Deveximentum sp DQ028038 Madagascar Marine Leiognathidae
Deveximentum sp DQ028037 Madagascar Marine Leiognathidae
Gazza squamiventralis DQ028008 Madagascar Marine Leiognathidae
Gazza sp DQ028004 Madagascar Marine Leiognathidae
Gazza sp DQ028003 Madagascar Marine Leiognathidae
Gazza sp DQ028002 Madagascar Marine Leiognathidae
Gazza sp DQ028001 Madagascar Marine Leiognathidae
Aurigequula fasciata DQ028021 Madagascar Marine Leiognathidae
Leiognathus sp DQ028012 Madagascar Marine Leiognathidae
Leiognathus sp DQ028013 Madagascar Marine Leiognathidae
Leiognathus sp DQ028011 Madagascar Marine Leiognathidae
Ecsenius midas JQ349945 Nosy Be Nosy Kivindry Marine Blenniidae
Ecsenius midas JQ349943 Nosy Be Nosy Kivindry Marine Blenniidae
Ecsenius midas JQ349944 Nosy Be Nosy Kivindry Marine Blenniidae
Ecsenius midas JQ349942 Nosy Be Nosy Kivindry Marine Blenniidae
Plectropomus laevis JQ350229 Nosy Be Nosy Tanikely Marine Serranidae
Priolepis cincta JQ350250 Nosy Be Nosy Kivindry Facultative Gobiidae
Priolepis cincta JQ350249 Nosy Be Nosy Kivindry Facultative Gobiidae
Priolepis cincta JQ350252 Nosy Be Nosy Kivindry Facultative Gobiidae
Priolepis inhaca JQ350253 Nosy Be Nosy Tanikely Sud Facultative Gobiidae
Eviota distigma JQ349971 Nosy Be Nosy Tanikely West Marine Gobiidae
Trimma macrophthalmum JQ350402 Nosy Be Nosy Kivindry Marine Gobiidae
Trimma macrophthalmum JQ350401 Nosy Be Nosy Kivindry Marine Gobiidae
Trimma mendelssohni JQ350404 Nosy Be Ambanoro bay Marine Gobiidae
Trimma mendelssohni JQ350407 Nosy Be Ambanoro bay Marine Gobiidae
Trimma mendelssohni JQ350406 Nosy Be Nosy Tanikely Sud Marine Gobiidae
Trimma mendelssohni JQ350405 Nosy Be Ambanoro bay Marine Gobiidae
Trimma mendelssohni JQ350403 Nosy Be Nosy Tanikely Sud Marine Gobiidae
Pseudochromis kristinae JQ350269 Nosy Be Nosy Tanikely West Marine Pseudochromidae
Pseudochromis kristinae JQ350267 Nosy Be Ambanoro bay Marine Pseudochromidae
Pseudochromis kristinae JQ350270 Nosy Be Nosy Tanikely West Marine Pseudochromidae
Pseudochromis kristinae JQ350271 Nosy Be Nosy Kivindry Marine Pseudochromidae
Pseudochromis kristinae JQ350272 Nosy Be Nosy Tanikely Sud Marine Pseudochromidae
Pseudochromis kristinae JQ350268 Nosy Be Nosy Tanikely West Marine Pseudochromidae
Pseudochromis kristinae JQ350274 Nosy Be Nosy Kivindry Marine Pseudochromidae
Pseudochromis kristinae JQ350275 Nosy Be Ambanoro bay Marine Pseudochromidae
Pseudochromis kristinae JQ350273 Nosy Be Nosy Kivindry Marine Pseudochromidae
Pseudochromis madagascariensis JQ350278 Nosy Be Ampasindava Marine Pseudochromidae
Pseudochromis madagascariensis JQ350277 Nosy Be Ampasindava Marine Pseudochromidae
Pseudochromis madagascariensis JQ350276 Nosy Be Nosy Vorona East Marine Pseudochromidae
Planiliza melinoptera RDR0658 Ratsianarana Facultative Mugilidae
Paretroplus petiti FGMV 2002 F A30 Madagascar Freshwater Cichlidae
Paretroplus petiti FGMV 2002 F A29 Madagascar Freshwater Cichlidae
Paretroplus petiti FGMV 2002 F A31 Madagascar Freshwater Cichlidae
Paretroplus petiti FGMV 2002 F A33 Madagascar Freshwater Cichlidae
Paretroplus petiti FGMV 2002 F A34 Madagascar Freshwater Cichlidae
Paretroplus petiti FGMV 2002 F A35 Madagascar Freshwater Cichlidae
Paretroplus petiti FGMV 2002 F A36 Madagascar Freshwater Cichlidae
Paretroplus petiti FGMV 2002 F A37 Madagascar Freshwater Cichlidae
Paretroplus petiti FGMV 2002 F A38 Madagascar Freshwater Cichlidae
Paretroplus petiti FGMV 2002 F A39 Madagascar Freshwater Cichlidae
Paretroplus petiti FGMV 2002 F A40 Madagascar Freshwater Cichlidae
Paretroplus petiti FGMV 2002 F A43 Madagascar Freshwater Cichlidae
Paretroplus petiti FGMV 2002 F A44 Madagascar Freshwater Cichlidae
Paretroplus petiti FGMV 2002 F A45 Madagascar Freshwater Cichlidae
Paretroplus petiti FGMV 2002 F A47 Madagascar Freshwater Cichlidae
Paretroplus petiti FGMV 2002 F A48 Madagascar Freshwater Cichlidae
Paretroplus petiti FGMV 2002 F A49 Madagascar Freshwater Cichlidae
Paretroplus petiti FGMV 2002 F A50 Madagascar Freshwater Cichlidae
Paretroplus petiti FGMV 2002 F A51 Madagascar Freshwater Cichlidae
Paretroplus petiti FGMV 2002 F A52 Madagascar Freshwater Cichlidae
Paretroplus petiti FGMV 2002 F A53 Madagascar Freshwater Cichlidae
Paretroplus petiti FGMV 2002 F A54 Madagascar Freshwater Cichlidae
Paretroplus petiti FGMV 2002 F A55 Madagascar Freshwater Cichlidae
Paretroplus petiti FGMV 2002 F A56 Madagascar Freshwater Cichlidae
Paretroplus petiti FGMV 2002 F A57 Madagascar Freshwater Cichlidae
Paretroplus petiti FGMV 2002 F A58 Madagascar Freshwater Cichlidae
Paretroplus petiti FGMV 2002 F A41 Madagascar Freshwater Cichlidae
Paretroplus petiti FGMV 2002 F A46 Madagascar Freshwater Cichlidae
Paretroplus maculatus FGMV 2002 F A21 Madagascar Freshwater Cichlidae
Paretroplus maculatus FGMV 2002 F A20 Madagascar Freshwater Cichlidae
Paretroplus maculatus FGMV 2002 F A19 Madagascar Freshwater Cichlidae
Paretroplus maculatus FGMV 2002 F A18 Madagascar Freshwater Cichlidae
Paretroplus maculatus FGMV 2002 F A17 Madagascar Freshwater Cichlidae
Paretroplus cf maromandia MVTIS13366 Madagascar Freshwater Cichlidae
Paretroplus menarambo TZTIS38 Madagascar Freshwater Cichlidae
Paretroplus menarambo TZTIS37 Madagascar Freshwater Cichlidae
Paretroplus menarambo TZTIS39 Madagascar Freshwater Cichlidae
Paretroplus menarambo TZTIS41 Madagascar Freshwater Cichlidae
Paretroplus menarambo TZTIS40 Madagascar Freshwater Cichlidae
Paretroplus kieneri FGMV 2002 F A26 Madagascar Freshwater Cichlidae
Paretroplus kieneri FGMV 2002 F A24 Madagascar Freshwater Cichlidae
Paretroplus kieneri FGMV 2002 F A22 Madagascar Freshwater Cichlidae
Paretroplus kieneri FGMV 2002 F A27 Madagascar Freshwater Cichlidae
Paretroplus kieneri FGMV 2002 F A25 Madagascar Freshwater Cichlidae
Paretroplus polyactis ZCMV438 Manombo village swamp Freshwater Cichlidae
Paretroplus polyactis FGMV 2002 F A08 Madagascar Freshwater Cichlidae
Paretroplus polyactis FGMV 2002 F A01 Madagascar Freshwater Cichlidae
Paretroplus nourissati FGMV 2002 F A10 Madagascar Freshwater Cichlidae
Paretroplus cf tsimoly MVTIS13370 probably Tsimoly Freshwater Cichlidae
Paretroplus nourissati MVTIS13369 Madagascar Freshwater Cichlidae
Paretroplus nourissati MVTIS13365 Madagascar Freshwater Cichlidae
Paretroplus cf damii Sambirano HQ702350 Madagascar Freshwater Cichlidae
Paretroplus lamenabe MVTIS13364 Madagascar Freshwater Cichlidae
Paretroplus sp ventitry HQ702349 Madagascar Freshwater Cichlidae
Paretroplus lamenabe MVTIS13363 Madagascar Freshwater Cichlidae

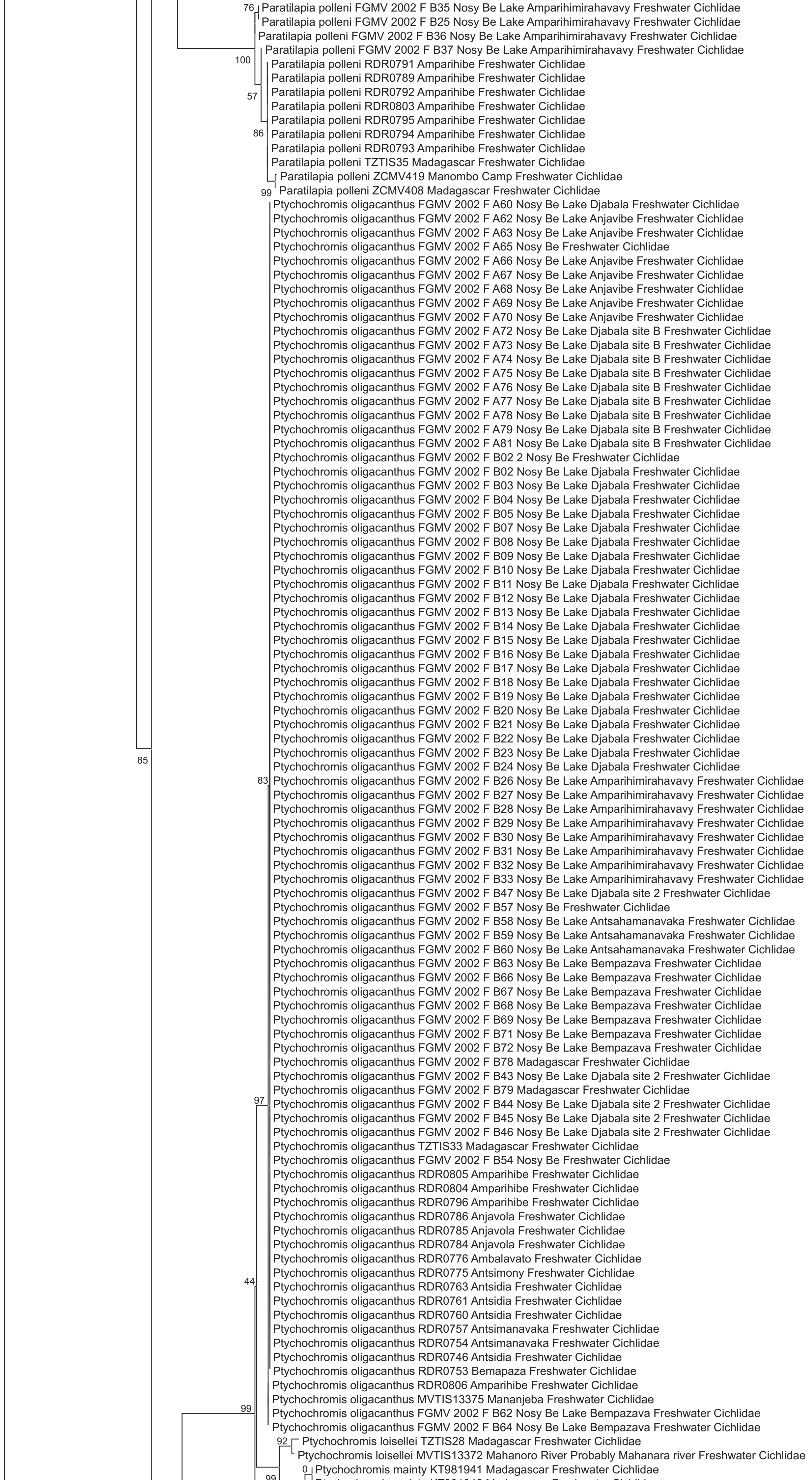

76 Paratilapia polleni FGMV 2002 F B35 Nosy Be Lake Amparihimirahavavy Freshwater Cichlidae
Paratilapia polleni FGMV 2002 F B25 Nosy Be Lake Amparihimirahavavy Freshwater Cichlidae
Paratilapia polleni FGMV 2002 F B36 Nosy Be Lake Amparihimirahavavy Freshwater Cichlidae
Paratilapia polleni FGMV 2002 F B37 Nosy Be Lake Amparihimirahavavy Freshwater Cichlidae
100 Paratilapia polleni RDR0791 Amparihibe Freshwater Cichlidae
Paratilapia polleni RDR0789 Amparihibe Freshwater Cichlidae
57 Paratilapia polleni RDR0792 Amparihibe Freshwater Cichlidae
Paratilapia polleni RDR0803 Amparihibe Freshwater Cichlidae
Paratilapia polleni RDR0795 Amparihibe Freshwater Cichlidae
86 Paratilapia polleni RDR0794 Amparihibe Freshwater Cichlidae
Paratilapia polleni RDR0793 Amparihibe Freshwater Cichlidae
Paratilapia polleni TZTIS35 Madagascar Freshwater Cichlidae
Paratilapia polleni ZCMV419 Manombo Camp Freshwater Cichlidae
99 Paratilapia polleni ZCMV408 Madagascar Freshwater Cichlidae
Ptychochromis oligacanthus FGMV 2002 F A60 Nosy Be Lake Djabala Freshwater Cichlidae
Ptychochromis oligacanthus FGMV 2002 F A62 Nosy Be Lake Anjavibe Freshwater Cichlidae
Ptychochromis oligacanthus FGMV 2002 F A63 Nosy Be Lake Anjavibe Freshwater Cichlidae
Ptychochromis oligacanthus FGMV 2002 F A65 Nosy Be Freshwater Cichlidae
Ptychochromis oligacanthus FGMV 2002 F A66 Nosy Be Lake Anjavibe Freshwater Cichlidae
Ptychochromis oligacanthus FGMV 2002 F A67 Nosy Be Lake Anjavibe Freshwater Cichlidae
Ptychochromis oligacanthus FGMV 2002 F A68 Nosy Be Lake Anjavibe Freshwater Cichlidae
Ptychochromis oligacanthus FGMV 2002 F A69 Nosy Be Lake Anjavibe Freshwater Cichlidae
Ptychochromis oligacanthus FGMV 2002 F A70 Nosy Be Lake Anjavibe Freshwater Cichlidae
Ptychochromis oligacanthus FGMV 2002 F A72 Nosy Be Lake Djabala site B Freshwater Cichlidae
Ptychochromis oligacanthus FGMV 2002 F A73 Nosy Be Lake Djabala site B Freshwater Cichlidae
Ptychochromis oligacanthus FGMV 2002 F A74 Nosy Be Lake Djabala site B Freshwater Cichlidae
Ptychochromis oligacanthus FGMV 2002 F A75 Nosy Be Lake Djabala site B Freshwater Cichlidae
Ptychochromis oligacanthus FGMV 2002 F A76 Nosy Be Lake Djabala site B Freshwater Cichlidae
Ptychochromis oligacanthus FGMV 2002 F A77 Nosy Be Lake Djabala site B Freshwater Cichlidae
Ptychochromis oligacanthus FGMV 2002 F A78 Nosy Be Lake Djabala site B Freshwater Cichlidae
Ptychochromis oligacanthus FGMV 2002 F A79 Nosy Be Lake Djabala site B Freshwater Cichlidae
Ptychochromis oligacanthus FGMV 2002 F A81 Nosy Be Lake Djabala site B Freshwater Cichlidae
Ptychochromis oligacanthus FGMV 2002 F B02 2 Nosy Be Freshwater Cichlidae
Ptychochromis oligacanthus FGMV 2002 F B02 Nosy Be Lake Djabala Freshwater Cichlidae
Ptychochromis oligacanthus FGMV 2002 F B03 Nosy Be Lake Djabala Freshwater Cichlidae
Ptychochromis oligacanthus FGMV 2002 F B04 Nosy Be Lake Djabala Freshwater Cichlidae
Ptychochromis oligacanthus FGMV 2002 F B05 Nosy Be Lake Djabala Freshwater Cichlidae
Ptychochromis oligacanthus FGMV 2002 F B07 Nosy Be Lake Djabala Freshwater Cichlidae
Ptychochromis oligacanthus FGMV 2002 F B08 Nosy Be Lake Djabala Freshwater Cichlidae
Ptychochromis oligacanthus FGMV 2002 F B09 Nosy Be Lake Djabala Freshwater Cichlidae
Ptychochromis oligacanthus FGMV 2002 F B10 Nosy Be Lake Djabala Freshwater Cichlidae
Ptychochromis oligacanthus FGMV 2002 F B11 Nosy Be Lake Djabala Freshwater Cichlidae
Ptychochromis oligacanthus FGMV 2002 F B12 Nosy Be Lake Djabala Freshwater Cichlidae
Ptychochromis oligacanthus FGMV 2002 F B13 Nosy Be Lake Djabala Freshwater Cichlidae
Ptychochromis oligacanthus FGMV 2002 F B14 Nosy Be Lake Djabala Freshwater Cichlidae
Ptychochromis oligacanthus FGMV 2002 F B15 Nosy Be Lake Djabala Freshwater Cichlidae
Ptychochromis oligacanthus FGMV 2002 F B16 Nosy Be Lake Djabala Freshwater Cichlidae
Ptychochromis oligacanthus FGMV 2002 F B17 Nosy Be Lake Djabala Freshwater Cichlidae
Ptychochromis oligacanthus FGMV 2002 F B18 Nosy Be Lake Djabala Freshwater Cichlidae
Ptychochromis oligacanthus FGMV 2002 F B19 Nosy Be Lake Djabala Freshwater Cichlidae
Ptychochromis oligacanthus FGMV 2002 F B20 Nosy Be Lake Djabala Freshwater Cichlidae
Ptychochromis oligacanthus FGMV 2002 F B21 Nosy Be Lake Djabala Freshwater Cichlidae
Ptychochromis oligacanthus FGMV 2002 F B22 Nosy Be Lake Djabala Freshwater Cichlidae
Ptychochromis oligacanthus FGMV 2002 F B23 Nosy Be Lake Djabala Freshwater Cichlidae
85
Ptychochromis oligacanthus FGMV 2002 F B24 Nosy Be Lake Djabala Freshwater Cichlidae
83 Ptychochromis oligacanthus FGMV 2002 F B26 Nosy Be Lake Amparihimirahavavy Freshwater Cichlidae
Ptychochromis oligacanthus FGMV 2002 F B27 Nosy Be Lake Amparihimirahavavy Freshwater Cichlidae
Ptychochromis oligacanthus FGMV 2002 F B28 Nosy Be Lake Amparihimirahavavy Freshwater Cichlidae
Ptychochromis oligacanthus FGMV 2002 F B29 Nosy Be Lake Amparihimirahavavy Freshwater Cichlidae
Ptychochromis oligacanthus FGMV 2002 F B30 Nosy Be Lake Amparihimirahavavy Freshwater Cichlidae
Ptychochromis oligacanthus FGMV 2002 F B31 Nosy Be Lake Amparihimirahavavy Freshwater Cichlidae
Ptychochromis oligacanthus FGMV 2002 F B32 Nosy Be Lake Amparihimirahavavy Freshwater Cichlidae
Ptychochromis oligacanthus FGMV 2002 F B33 Nosy Be Lake Amparihimirahavavy Freshwater Cichlidae
Ptychochromis oligacanthus FGMV 2002 F B47 Nosy Be Lake Djabala site 2 Freshwater Cichlidae
Ptychochromis oligacanthus FGMV 2002 F B57 Nosy Be Freshwater Cichlidae
Ptychochromis oligacanthus FGMV 2002 F B58 Nosy Be Lake Antsahamanavaka Freshwater Cichlidae
Ptychochromis oligacanthus FGMV 2002 F B59 Nosy Be Lake Antsahamanavaka Freshwater Cichlidae
Ptychochromis oligacanthus FGMV 2002 F B60 Nosy Be Lake Antsahamanavaka Freshwater Cichlidae
Ptychochromis oligacanthus FGMV 2002 F B63 Nosy Be Lake Bempazava Freshwater Cichlidae
Ptychochromis oligacanthus FGMV 2002 F B66 Nosy Be Lake Bempazava Freshwater Cichlidae
Ptychochromis oligacanthus FGMV 2002 F B67 Nosy Be Lake Bempazava Freshwater Cichlidae
Ptychochromis oligacanthus FGMV 2002 F B68 Nosy Be Lake Bempazava Freshwater Cichlidae
Ptychochromis oligacanthus FGMV 2002 F B69 Nosy Be Lake Bempazava Freshwater Cichlidae
Ptychochromis oligacanthus FGMV 2002 F B71 Nosy Be Lake Bempazava Freshwater Cichlidae
Ptychochromis oligacanthus FGMV 2002 F B72 Nosy Be Lake Bempazava Freshwater Cichlidae
Ptychochromis oligacanthus FGMV 2002 F B78 Madagascar Freshwater Cichlidae
Ptychochromis oligacanthus FGMV 2002 F B43 Nosy Be Lake Djabala site 2 Freshwater Cichlidae
Ptychochromis oligacanthus FGMV 2002 F B79 Madagascar Freshwater Cichlidae
97 Ptychochromis oligacanthus FGMV 2002 F B44 Nosy Be Lake Djabala site 2 Freshwater Cichlidae
Ptychochromis oligacanthus FGMV 2002 F B45 Nosy Be Lake Djabala site 2 Freshwater Cichlidae
Ptychochromis oligacanthus FGMV 2002 F B46 Nosy Be Lake Djabala site 2 Freshwater Cichlidae
Ptychochromis oligacanthus TZTIS33 Madagascar Freshwater Cichlidae
Ptychochromis oligacanthus FGMV 2002 F B54 Nosy Be Freshwater Cichlidae
Ptychochromis oligacanthus RDR0805 Amparihibe Freshwater Cichlidae
Ptychochromis oligacanthus RDR0804 Amparihibe Freshwater Cichlidae
Ptychochromis oligacanthus RDR0796 Amparihibe Freshwater Cichlidae
Ptychochromis oligacanthus RDR0786 Anjavola Freshwater Cichlidae
Ptychochromis oligacanthus RDR0785 Anjavola Freshwater Cichlidae
Ptychochromis oligacanthus RDR0784 Anjavola Freshwater Cichlidae
Ptychochromis oligacanthus RDR0776 Ambalavato Freshwater Cichlidae
Ptychochromis oligacanthus RDR0775 Antsimony Freshwater Cichlidae
44 Ptychochromis oligacanthus RDR0763 Antsidia Freshwater Cichlidae
Ptychochromis oligacanthus RDR0761 Antsidia Freshwater Cichlidae
Ptychochromis oligacanthus RDR0760 Antsidia Freshwater Cichlidae
Ptychochromis oligacanthus RDR0757 Antsimanavaka Freshwater Cichlidae
Ptychochromis oligacanthus RDR0754 Antsimanavaka Freshwater Cichlidae
Ptychochromis oligacanthus RDR0746 Antsidia Freshwater Cichlidae
Ptychochromis oligacanthus RDR0753 Bemapaza Freshwater Cichlidae
Ptychochromis oligacanthus RDR0806 Amparihibe Freshwater Cichlidae
Ptychochromis oligacanthus MVTIS13375 Mananjeba Freshwater Cichlidae
99 Ptychochromis oligacanthus FGMV 2002 F B62 Nosy Be Lake Bempazava Freshwater Cichlidae
Ptychochromis oligacanthus FGMV 2002 F B64 Nosy Be Lake Bempazava Freshwater Cichlidae
92 Ptychochromis loisellei TZTIS28 Madagascar Freshwater Cichlidae
Ptychochromis loisellei MVTIS13372 Mahanoro River Probably Mahanara river Freshwater Cichlidae
0 Ptychochromis mainty KT981941 Madagascar Freshwater Cichlidae
99

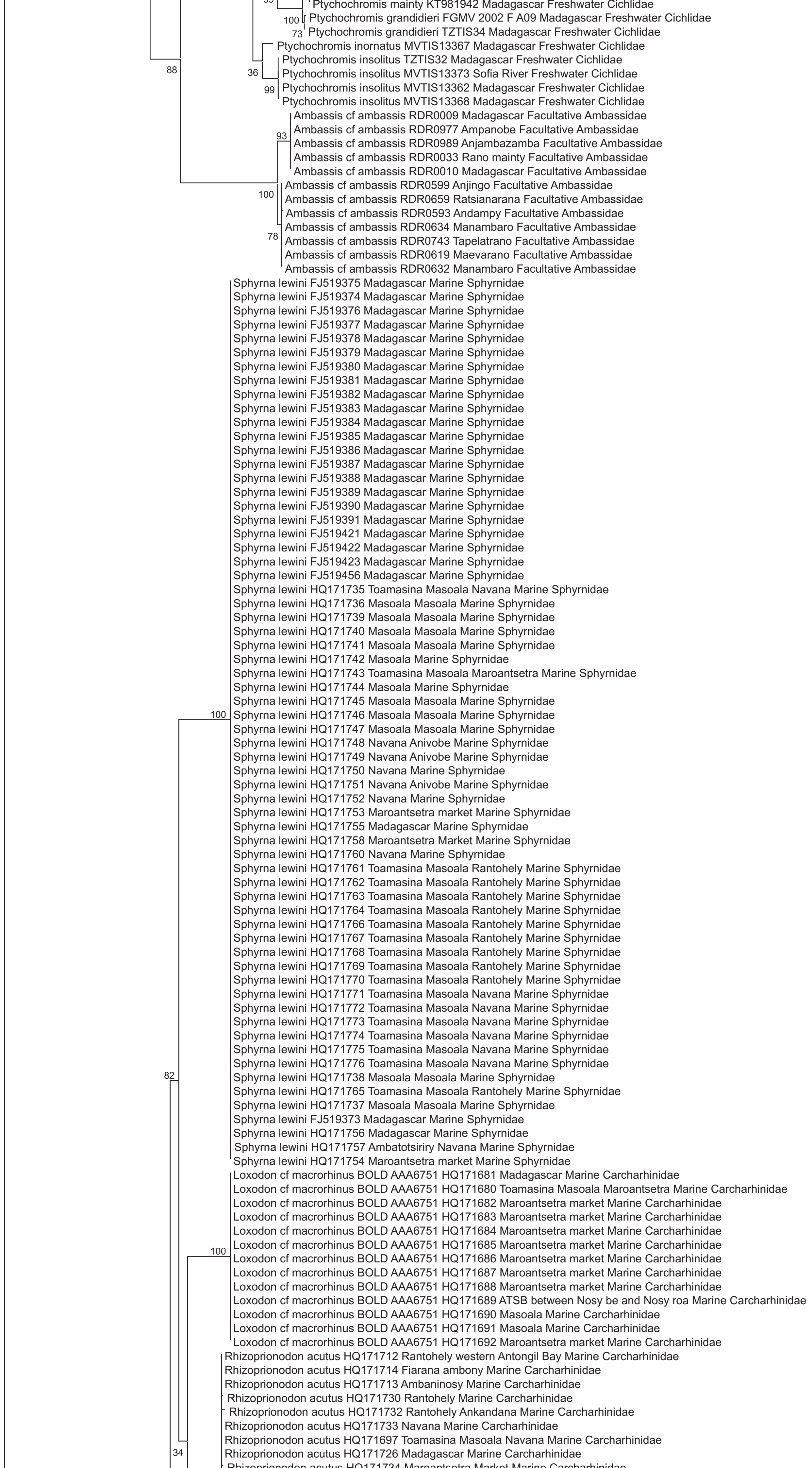
Ptychochromis mainty KT981942 Madagascar Freshwater Cichlidae
100
Ptychochromis grandidieri FGMV 2002 F A09 Madagascar Freshwater Cichlidae
73
Ptychochromis grandidieri TZTIS34 Madagascar Freshwater Cichlidae
Ptychochromis inornatus MVTIS13367 Madagascar Freshwater Cichlidae
88
Ptychochromis insolitus TZTIS32 Madagascar Freshwater Cichlidae
36
Ptychochromis insolitus MVTIS13373 Sofia River Freshwater Cichlidae
99
Ptychochromis insolitus MVTIS13362 Madagascar Freshwater Cichlidae
Ptychochromis insolitus MVTIS13368 Madagascar Freshwater Cichlidae
Ambassis cf ambassis RDR0009 Madagascar Facultative Ambassidae
Ambassis cf ambassis RDR0977 Ampanobe Facultative Ambassidae
93
Ambassis cf ambassis RDR0989 Anjambazamba Facultative Ambassidae
Ambassis cf ambassis RDR0033 Rano mainty Facultative Ambassidae
Ambassis cf ambassis RDR0010 Madagascar Facultative Ambassidae
Ambassis cf ambassis RDR0599 Anjingo Facultative Ambassidae
100
Ambassis cf ambassis RDR0659 Ratsianarana Facultative Ambassidae
Ambassis cf ambassis RDR0593 Andampy Facultative Ambassidae
Ambassis cf ambassis RDR0634 Manambaro Facultative Ambassidae
78
Ambassis cf ambassis RDR0743 Tapelatrano Facultative Ambassidae
Ambassis cf ambassis RDR0619 Maevarano Facultative Ambassidae
Ambassis cf ambassis RDR0632 Manambaro Facultative Ambassidae
Sphyrna lewini FJ519375 Madagascar Marine Sphyrnidae
Sphyrna lewini FJ519374 Madagascar Marine Sphyrnidae
Sphyrna lewini FJ519376 Madagascar Marine Sphyrnidae
Sphyrna lewini FJ519377 Madagascar Marine Sphyrnidae
Sphyrna lewini FJ519378 Madagascar Marine Sphyrnidae
Sphyrna lewini FJ519379 Madagascar Marine Sphyrnidae
Sphyrna lewini FJ519380 Madagascar Marine Sphyrnidae
Sphyrna lewini FJ519381 Madagascar Marine Sphyrnidae
Sphyrna lewini FJ519382 Madagascar Marine Sphyrnidae
Sphyrna lewini FJ519383 Madagascar Marine Sphyrnidae
Sphyrna lewini FJ519384 Madagascar Marine Sphyrnidae
Sphyrna lewini FJ519385 Madagascar Marine Sphyrnidae
Sphyrna lewini FJ519386 Madagascar Marine Sphyrnidae
Sphyrna lewini FJ519387 Madagascar Marine Sphyrnidae
Sphyrna lewini FJ519388 Madagascar Marine Sphyrnidae
Sphyrna lewini FJ519389 Madagascar Marine Sphyrnidae
Sphyrna lewini FJ519390 Madagascar Marine Sphyrnidae
Sphyrna lewini FJ519391 Madagascar Marine Sphyrnidae
Sphyrna lewini FJ519421 Madagascar Marine Sphyrnidae
Sphyrna lewini FJ519422 Madagascar Marine Sphyrnidae
Sphyrna lewini FJ519423 Madagascar Marine Sphyrnidae
Sphyrna lewini FJ519456 Madagascar Marine Sphyrnidae
Sphyrna lewini HQ171735 Toamasina Masoala Navana Marine Sphyrnidae
Sphyrna lewini HQ171736 Masoala Masoala Marine Sphyrnidae
Sphyrna lewini HQ171739 Masoala Masoala Marine Sphyrnidae
Sphyrna lewini HQ171740 Masoala Masoala Marine Sphyrnidae
Sphyrna lewini HQ171741 Masoala Masoala Marine Sphyrnidae
Sphyrna lewini HQ171742 Masoala Marine Sphyrnidae
Sphyrna lewini HQ171743 Toamasina Masoala Maroantsetra Marine Sphyrnidae
Sphyrna lewini HQ171744 Masoala Marine Sphyrnidae
Sphyrna lewini HQ171745 Masoala Masoala Marine Sphyrnidae
100
Sphyrna lewini HQ171746 Masoala Masoala Marine Sphyrnidae
Sphyrna lewini HQ171747 Masoala Masoala Marine Sphyrnidae
Sphyrna lewini HQ171748 Navana Anivobe Marine Sphyrnidae
Sphyrna lewini HQ171749 Navana Anivobe Marine Sphyrnidae
Sphyrna lewini HQ171750 Navana Marine Sphyrnidae
Sphyrna lewini HQ171751 Navana Anivobe Marine Sphyrnidae
Sphyrna lewini HQ171752 Navana Marine Sphyrnidae
Sphyrna lewini HQ171753 Maroantsetra market Marine Sphyrnidae
Sphyrna lewini HQ171755 Madagascar Marine Sphyrnidae
Sphyrna lewini HQ171758 Maroantsetra Market Marine Sphyrnidae
Sphyrna lewini HQ171760 Navana Marine Sphyrnidae
Sphyrna lewini HQ171761 Toamasina Masoala Rantohely Marine Sphyrnidae
Sphyrna lewini HQ171762 Toamasina Masoala Rantohely Marine Sphyrnidae
Sphyrna lewini HQ171763 Toamasina Masoala Rantohely Marine Sphyrnidae
Sphyrna lewini HQ171764 Toamasina Masoala Rantohely Marine Sphyrnidae
Sphyrna lewini HQ171766 Toamasina Masoala Rantohely Marine Sphyrnidae
Sphyrna lewini HQ171767 Toamasina Masoala Rantohely Marine Sphyrnidae
Sphyrna lewini HQ171768 Toamasina Masoala Rantohely Marine Sphyrnidae
Sphyrna lewini HQ171769 Toamasina Masoala Rantohely Marine Sphyrnidae
Sphyrna lewini HQ171770 Toamasina Masoala Rantohely Marine Sphyrnidae
Sphyrna lewini HQ171771 Toamasina Masoala Navana Marine Sphyrnidae
Sphyrna lewini HQ171772 Toamasina Masoala Navana Marine Sphyrnidae
Sphyrna lewini HQ171773 Toamasina Masoala Navana Marine Sphyrnidae
Sphyrna lewini HQ171774 Toamasina Masoala Navana Marine Sphyrnidae
Sphyrna lewini HQ171775 Toamasina Masoala Navana Marine Sphyrnidae
Sphyrna lewini HQ171776 Toamasina Masoala Navana Marine Sphyrnidae
82
Sphyrna lewini HQ171738 Masoala Masoala Marine Sphyrnidae
Sphyrna lewini HQ171765 Toamasina Masoala Rantohely Marine Sphyrnidae
Sphyrna lewini HQ171737 Masoala Masoala Marine Sphyrnidae
Sphyrna lewini FJ519373 Madagascar Marine Sphyrnidae
Sphyrna lewini HQ171756 Madagascar Marine Sphyrnidae
Sphyrna lewini HQ171757 Ambatotsiriry Navana Marine Sphyrnidae
Sphyrna lewini HQ171754 Maroantsetra market Marine Sphyrnidae
Loxodon cf macrorhinus BOLD AAA6751 HQ171681 Madagascar Marine Carcharhinidae
Loxodon cf macrorhinus BOLD AAA6751 HQ171680 Toamasina Masoala Maroantsetra Marine Carcharhinidae
Loxodon cf macrorhinus BOLD AAA6751 HQ171682 Maroantsetra market Marine Carcharhinidae
Loxodon cf macrorhinus BOLD AAA6751 HQ171683 Maroantsetra market Marine Carcharhinidae
Loxodon cf macrorhinus BOLD AAA6751 HQ171684 Maroantsetra market Marine Carcharhinidae
Loxodon cf macrorhinus BOLD AAA6751 HQ171685 Maroantsetra market Marine Carcharhinidae
100
Loxodon cf macrorhinus BOLD AAA6751 HQ171686 Maroantsetra market Marine Carcharhinidae
Loxodon cf macrorhinus BOLD AAA6751 HQ171687 Maroantsetra market Marine Carcharhinidae
Loxodon cf macrorhinus BOLD AAA6751 HQ171688 Maroantsetra market Marine Carcharhinidae
Loxodon cf macrorhinus BOLD AAA6751 HQ171689 ATSB between Nosy be and Nosy roa Marine Carcharhinidae
Loxodon cf macrorhinus BOLD AAA6751 HQ171690 Masoala Marine Carcharhinidae
Loxodon cf macrorhinus BOLD AAA6751 HQ171691 Masoala Marine Carcharhinidae
Loxodon cf macrorhinus BOLD AAA6751 HQ171692 Maroantsetra market Marine Carcharhinidae
Rhizoprionodon acutus HQ171712 Rantohely western Antongil Bay Marine Carcharhinidae
Rhizoprionodon acutus HQ171714 Fiarana ambony Marine Carcharhinidae
Rhizoprionodon acutus HQ171713 Ambaninosy Marine Carcharhinidae
Rhizoprionodon acutus HQ171730 Rantohely Marine Carcharhinidae
Rhizoprionodon acutus HQ171732 Rantohely Ankandana Marine Carcharhinidae
Rhizoprionodon acutus HQ171733 Navana Marine Carcharhinidae
Rhizoprionodon acutus HQ171697 Toamasina Masoala Navana Marine Carcharhinidae
34
Rhizoprionodon acutus HQ171726 Madagascar Marine Carcharhinidae
Rhizoprionodon acutus HQ171734 Maroantsetra Market Marine Carcharhinidae

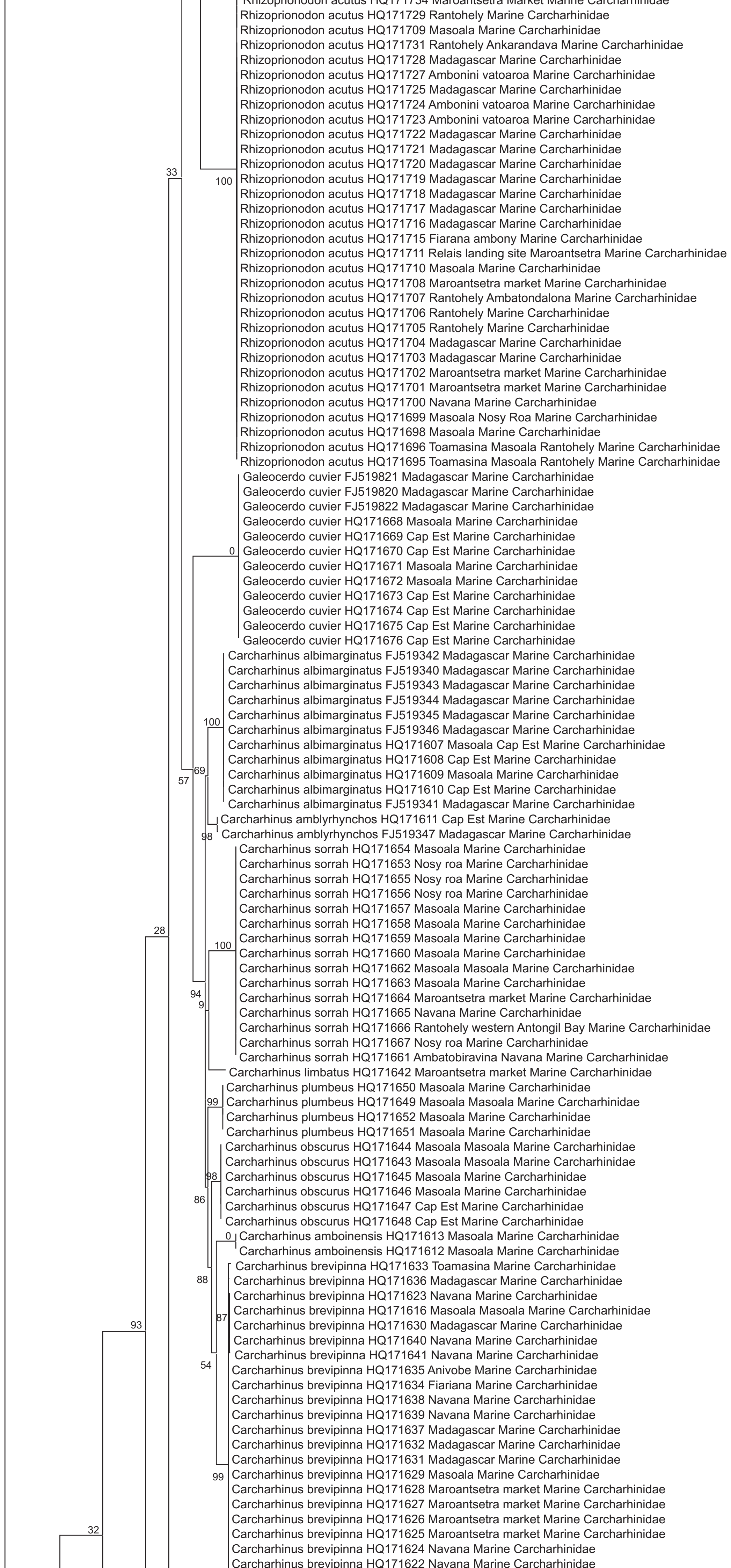
Rhizoprionodon acutus HQ171729 Rantohely Marine Carcharhinidae
Rhizoprionodon acutus HQ171709 Masoala Marine Carcharhinidae
Rhizoprionodon acutus HQ171731 Rantohely Ankarandava Marine Carcharhinidae
Rhizoprionodon acutus HQ171728 Madagascar Marine Carcharhinidae
Rhizoprionodon acutus HQ171727 Ambonini vatoaroa Marine Carcharhinidae
Rhizoprionodon acutus HQ171725 Madagascar Marine Carcharhinidae
Rhizoprionodon acutus HQ171724 Ambonini vatoaroa Marine Carcharhinidae
Rhizoprionodon acutus HQ171723 Ambonini vatoaroa Marine Carcharhinidae
Rhizoprionodon acutus HQ171722 Madagascar Marine Carcharhinidae
Rhizoprionodon acutus HQ171721 Madagascar Marine Carcharhinidae
Rhizoprionodon acutus HQ171720 Madagascar Marine Carcharhinidae
33
100
Rhizoprionodon acutus HQ171719 Madagascar Marine Carcharhinidae
Rhizoprionodon acutus HQ171718 Madagascar Marine Carcharhinidae
Rhizoprionodon acutus HQ171717 Madagascar Marine Carcharhinidae
Rhizoprionodon acutus HQ171716 Madagascar Marine Carcharhinidae
Rhizoprionodon acutus HQ171715 Fiarana ambony Marine Carcharhinidae
Rhizoprionodon acutus HQ171711 Relais landing site Maroantsetra Marine Carcharhinidae
Rhizoprionodon acutus HQ171710 Masoala Marine Carcharhinidae
Rhizoprionodon acutus HQ171708 Maroantsetra market Marine Carcharhinidae
Rhizoprionodon acutus HQ171707 Rantohely Ambatondalona Marine Carcharhinidae
Rhizoprionodon acutus HQ171706 Rantohely Marine Carcharhinidae
Rhizoprionodon acutus HQ171705 Rantohely Marine Carcharhinidae
Rhizoprionodon acutus HQ171704 Madagascar Marine Carcharhinidae
Rhizoprionodon acutus HQ171703 Madagascar Marine Carcharhinidae
Rhizoprionodon acutus HQ171702 Maroantsetra market Marine Carcharhinidae
Rhizoprionodon acutus HQ171701 Maroantsetra market Marine Carcharhinidae
Rhizoprionodon acutus HQ171700 Navana Marine Carcharhinidae
Rhizoprionodon acutus HQ171699 Masoala Nosy Roa Marine Carcharhinidae
Rhizoprionodon acutus HQ171698 Masoala Marine Carcharhinidae
Rhizoprionodon acutus HQ171696 Toamasina Masoala Rantohely Marine Carcharhinidae
Rhizoprionodon acutus HQ171695 Toamasina Masoala Rantohely Marine Carcharhinidae
Galeocerdo cuvier FJ519821 Madagascar Marine Carcharhinidae
Galeocerdo cuvier FJ519820 Madagascar Marine Carcharhinidae
Galeocerdo cuvier FJ519822 Madagascar Marine Carcharhinidae
Galeocerdo cuvier HQ171668 Masoala Marine Carcharhinidae
Galeocerdo cuvier HQ171669 Cap Est Marine Carcharhinidae
0
Galeocerdo cuvier HQ171670 Cap Est Marine Carcharhinidae
Galeocerdo cuvier HQ171671 Masoala Marine Carcharhinidae
Galeocerdo cuvier HQ171672 Masoala Marine Carcharhinidae
Galeocerdo cuvier HQ171673 Cap Est Marine Carcharhinidae
Galeocerdo cuvier HQ171674 Cap Est Marine Carcharhinidae
Galeocerdo cuvier HQ171675 Cap Est Marine Carcharhinidae
Galeocerdo cuvier HQ171676 Cap Est Marine Carcharhinidae
Carcharhinus albimarginatus FJ519342 Madagascar Marine Carcharhinidae
Carcharhinus albimarginatus FJ519340 Madagascar Marine Carcharhinidae
Carcharhinus albimarginatus FJ519343 Madagascar Marine Carcharhinidae
Carcharhinus albimarginatus FJ519344 Madagascar Marine Carcharhinidae
100
Carcharhinus albimarginatus FJ519345 Madagascar Marine Carcharhinidae
Carcharhinus albimarginatus FJ519346 Madagascar Marine Carcharhinidae
Carcharhinus albimarginatus HQ171607 Masoala Cap Est Marine Carcharhinidae
Carcharhinus albimarginatus HQ171608 Cap Est Marine Carcharhinidae
69
57
Carcharhinus albimarginatus HQ171609 Masoala Marine Carcharhinidae
Carcharhinus albimarginatus HQ171610 Cap Est Marine Carcharhinidae
Carcharhinus albimarginatus FJ519341 Madagascar Marine Carcharhinidae
Carcharhinus amblyrhynchos HQ171611 Cap Est Marine Carcharhinidae
98
Carcharhinus amblyrhynchos FJ519347 Madagascar Marine Carcharhinidae
Carcharhinus sorrah HQ171654 Masoala Marine Carcharhinidae
Carcharhinus sorrah HQ171653 Nosy roa Marine Carcharhinidae
Carcharhinus sorrah HQ171655 Nosy roa Marine Carcharhinidae
Carcharhinus sorrah HQ171656 Nosy roa Marine Carcharhinidae
Carcharhinus sorrah HQ171657 Masoala Marine Carcharhinidae
Carcharhinus sorrah HQ171658 Masoala Marine Carcharhinidae
28
Carcharhinus sorrah HQ171659 Masoala Marine Carcharhinidae
100
Carcharhinus sorrah HQ171660 Masoala Marine Carcharhinidae
Carcharhinus sorrah HQ171662 Masoala Masoala Marine Carcharhinidae
Carcharhinus sorrah HQ171663 Masoala Marine Carcharhinidae
94
9
Carcharhinus sorrah HQ171664 Maroantsetra market Marine Carcharhinidae
Carcharhinus sorrah HQ171665 Navana Marine Carcharhinidae
Carcharhinus sorrah HQ171666 Rantohely western Antongil Bay Marine Carcharhinidae
Carcharhinus sorrah HQ171667 Nosy roa Marine Carcharhinidae
Carcharhinus sorrah HQ171661 Ambatobiravina Navana Marine Carcharhinidae
Carcharhinus limbatus HQ171642 Maroantsetra market Marine Carcharhinidae
Carcharhinus plumbeus HQ171650 Masoala Marine Carcharhinidae
99
Carcharhinus plumbeus HQ171649 Masoala Masoala Marine Carcharhinidae
Carcharhinus plumbeus HQ171652 Masoala Marine Carcharhinidae
Carcharhinus plumbeus HQ171651 Masoala Marine Carcharhinidae
Carcharhinus obscurus HQ171644 Masoala Masoala Marine Carcharhinidae
Carcharhinus obscurus HQ171643 Masoala Masoala Marine Carcharhinidae
98
Carcharhinus obscurus HQ171645 Masoala Marine Carcharhinidae
Carcharhinus obscurus HQ171646 Masoala Marine Carcharhinidae
86
Carcharhinus obscurus HQ171647 Cap Est Marine Carcharhinidae
Carcharhinus obscurus HQ171648 Cap Est Marine Carcharhinidae
0
Carcharhinus amboinensis HQ171613 Masoala Marine Carcharhinidae
Carcharhinus amboinensis HQ171612 Masoala Marine Carcharhinidae
Carcharhinus brevipinna HQ171633 Toamasina Marine Carcharhinidae
88
Carcharhinus brevipinna HQ171636 Madagascar Marine Carcharhinidae
Carcharhinus brevipinna HQ171623 Navana Marine Carcharhinidae
87
Carcharhinus brevipinna HQ171616 Masoala Masoala Marine Carcharhinidae
93
Carcharhinus brevipinna HQ171630 Madagascar Marine Carcharhinidae
Carcharhinus brevipinna HQ171640 Navana Marine Carcharhinidae
Carcharhinus brevipinna HQ171641 Navana Marine Carcharhinidae
54
Carcharhinus brevipinna HQ171635 Anivobe Marine Carcharhinidae
Carcharhinus brevipinna HQ171634 Fiariana Marine Carcharhinidae
Carcharhinus brevipinna HQ171638 Navana Marine Carcharhinidae
Carcharhinus brevipinna HQ171639 Navana Marine Carcharhinidae
Carcharhinus brevipinna HQ171637 Madagascar Marine Carcharhinidae
Carcharhinus brevipinna HQ171632 Madagascar Marine Carcharhinidae
Carcharhinus brevipinna HQ171631 Madagascar Marine Carcharhinidae
99
Carcharhinus brevipinna HQ171629 Masoala Marine Carcharhinidae
Carcharhinus brevipinna HQ171628 Maroantsetra market Marine Carcharhinidae
Carcharhinus brevipinna HQ171627 Maroantsetra market Marine Carcharhinidae
Carcharhinus brevipinna HQ171626 Maroantsetra market Marine Carcharhinidae
32
Carcharhinus brevipinna HQ171625 Maroantsetra market Marine Carcharhinidae
Carcharhinus brevipinna HQ171624 Navana Marine Carcharhinidae
Carcharhinus brevipinna HQ171622 Navana Marine Carcharhinidae

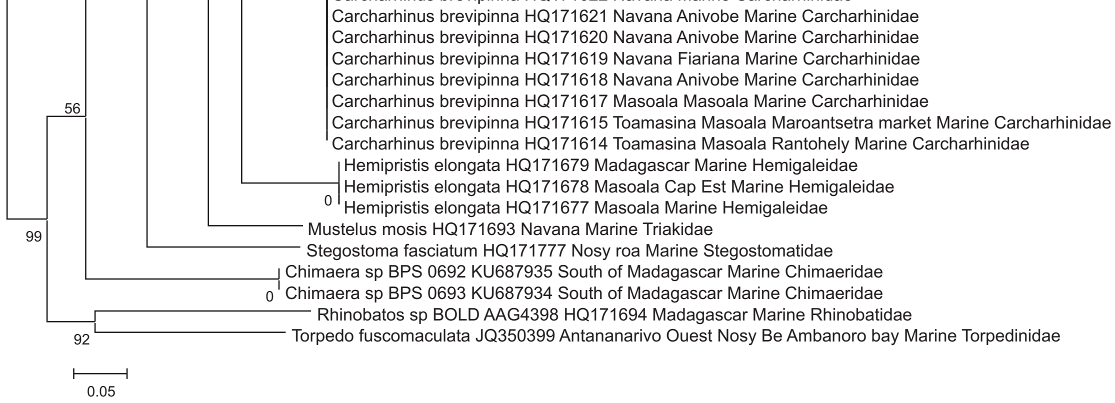
Carcharhinus brevipinna HQ171621 Navana Anivobe Marine Carcharhinidae
Carcharhinus brevipinna HQ171620 Navana Anivobe Marine Carcharhinidae
Carcharhinus brevipinna HQ171619 Navana Fiariana Marine Carcharhinidae
Carcharhinus brevipinna HQ171618 Navana Anivobe Marine Carcharhinidae
Carcharhinus brevipinna HQ171617 Masoala Masoala Marine Carcharhinidae
Carcharhinus brevipinna HQ171615 Toamasina Masoala Maroantsetra market Marine Carcharhinidae
Carcharhinus brevipinna HQ171614 Toamasina Masoala Rantohely Marine Carcharhinidae
Hemipristis elongata HQ171679 Madagascar Marine Hemigaleidae
Hemipristis elongata HQ171678 Masoala Cap Est Marine Hemigaleidae
Hemipristis elongata HQ171677 Masoala Marine Hemigaleidae
Mustelus mosis HQ171693 Navana Marine Triakidae
Stegostoma fasciatum HQ171777 Nosy roa Marine Stegostomatidae
Chimaera sp BPS 0692 KU687935 South of Madagascar Marine Chimaeridae
Chimaera sp BPS 0693 KU687934 South of Madagascar Marine Chimaeridae
Rhinobatos sp BOLD AAG4398 HQ171694 Madagascar Marine Rhinobatidae
Torpedo fuscomaculata JQ350399 Antananarivo Ouest Nosy Be Ambanoro bay Marine Torpedinidae
56
0
99
0
92
0.05
