# Supplementary figures and images for: Towards a DNA barcode library for Madagascar’s threatened ichthyofauna
Source: PLoS One. 2022 Aug 11;17(8):e0271400. doi: 10.1371/journal.pone.0271400 (PMC9371263; doi:10.1371/journal.pone.0271400)

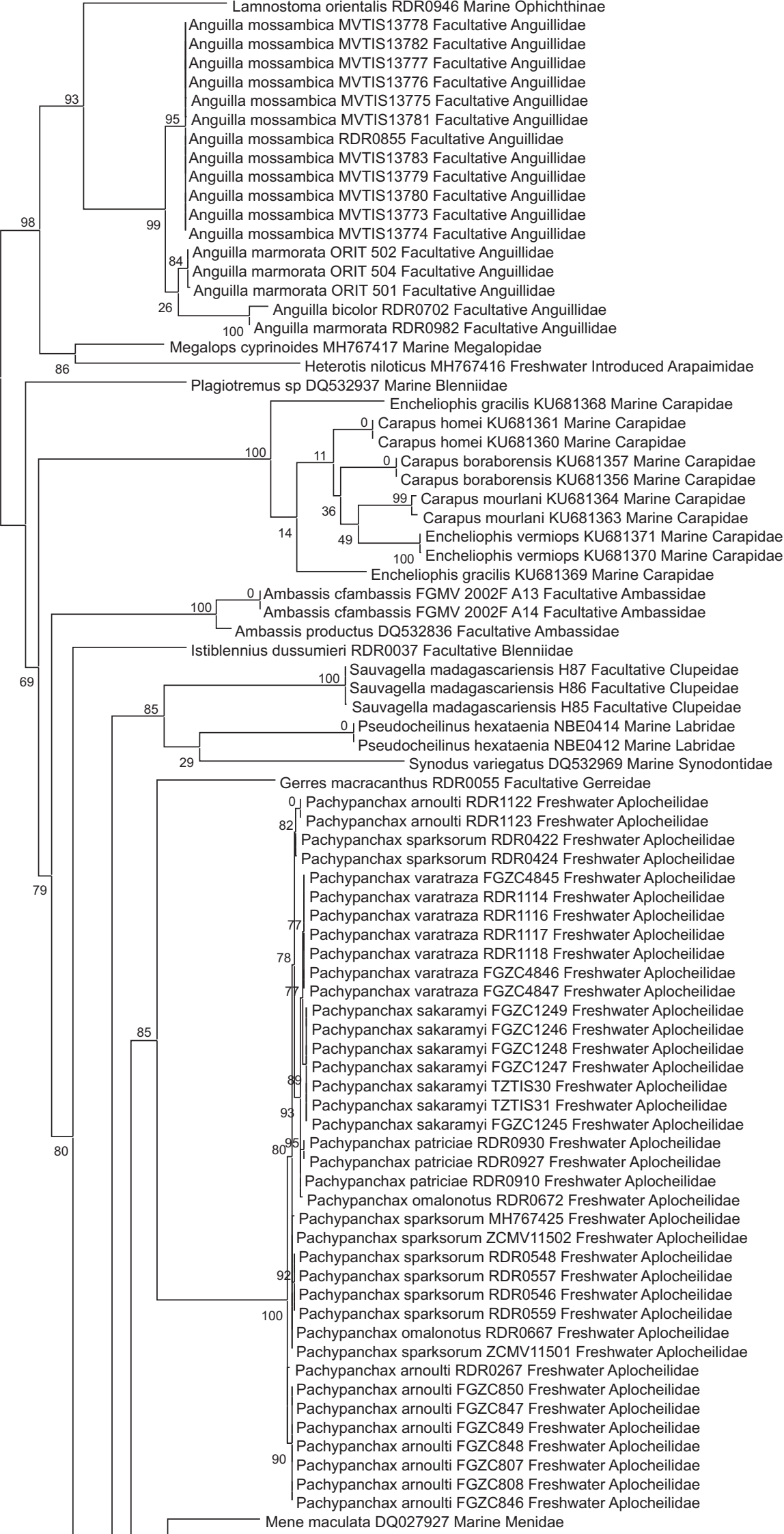

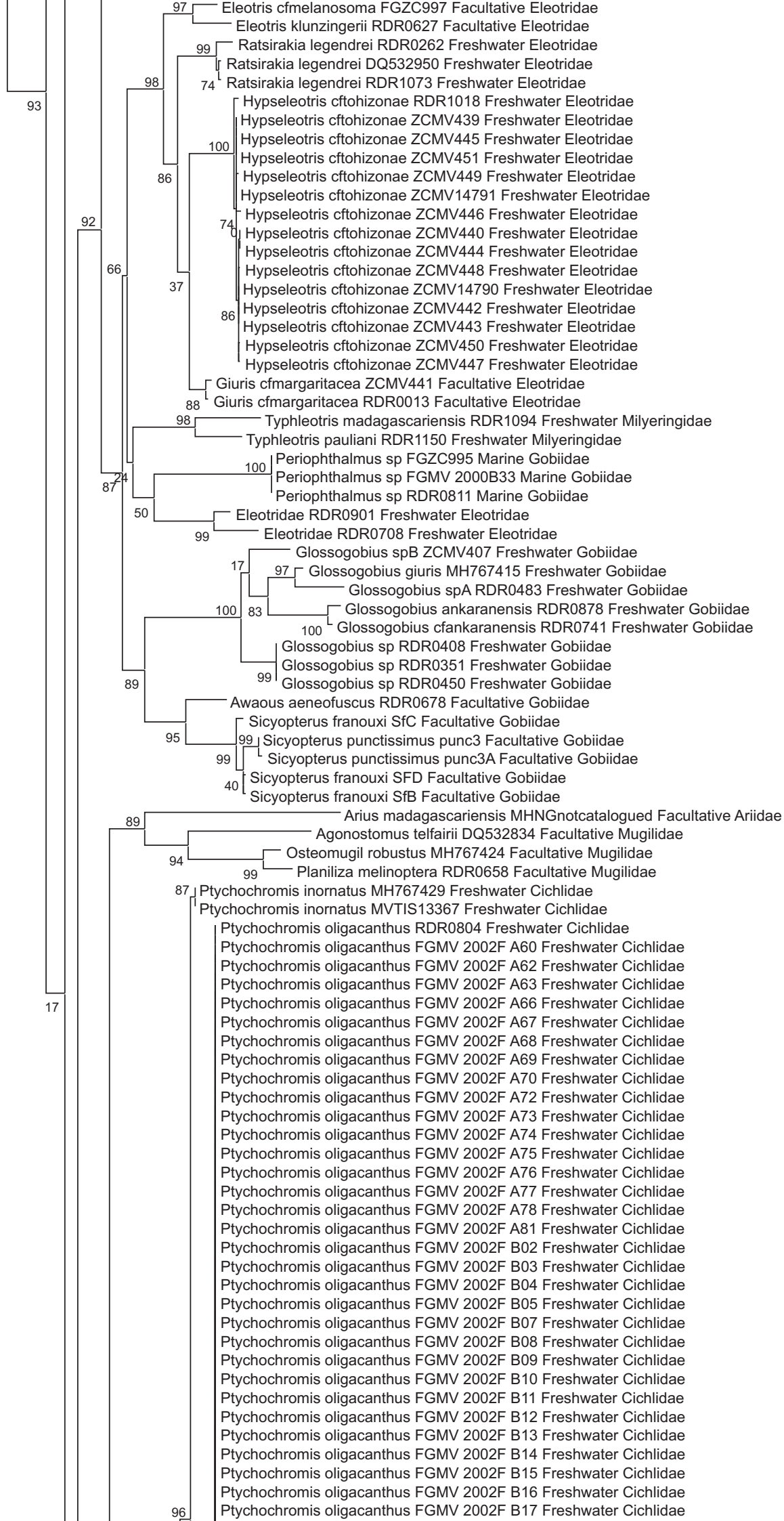

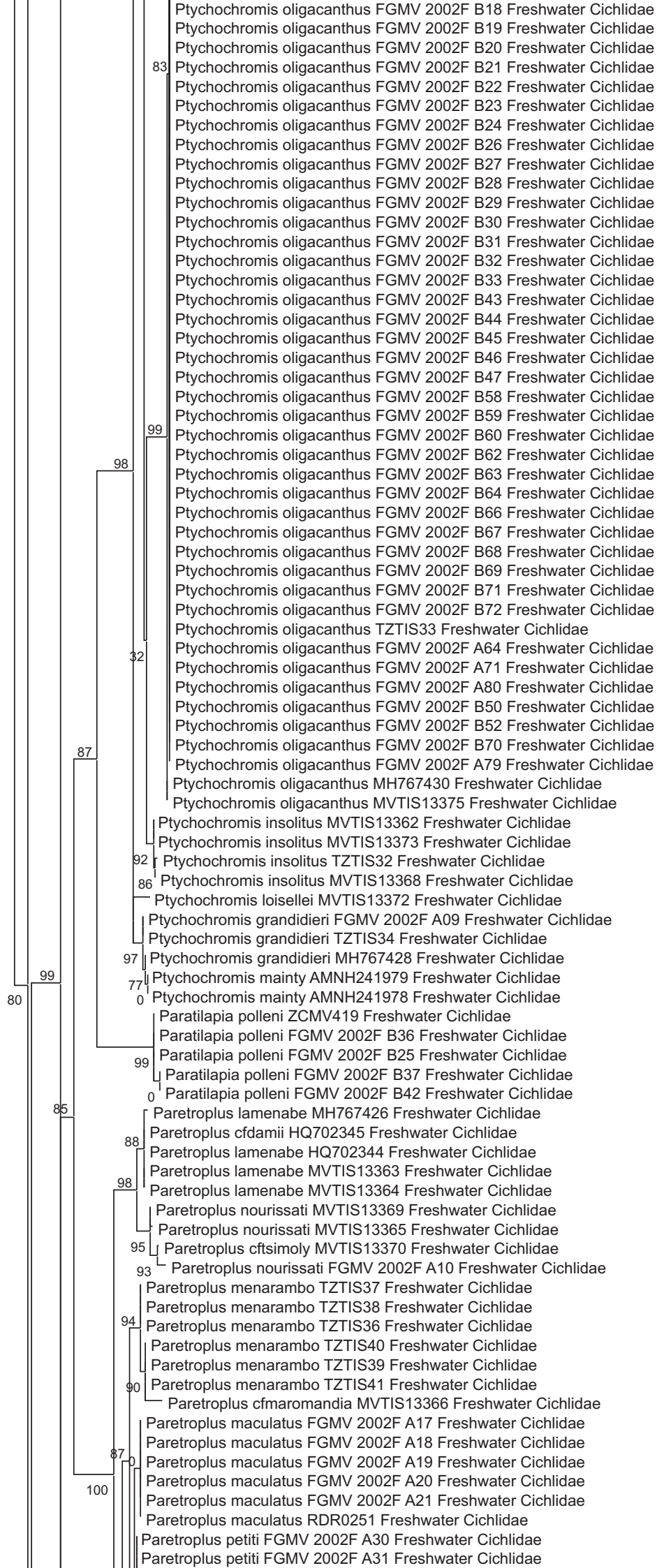

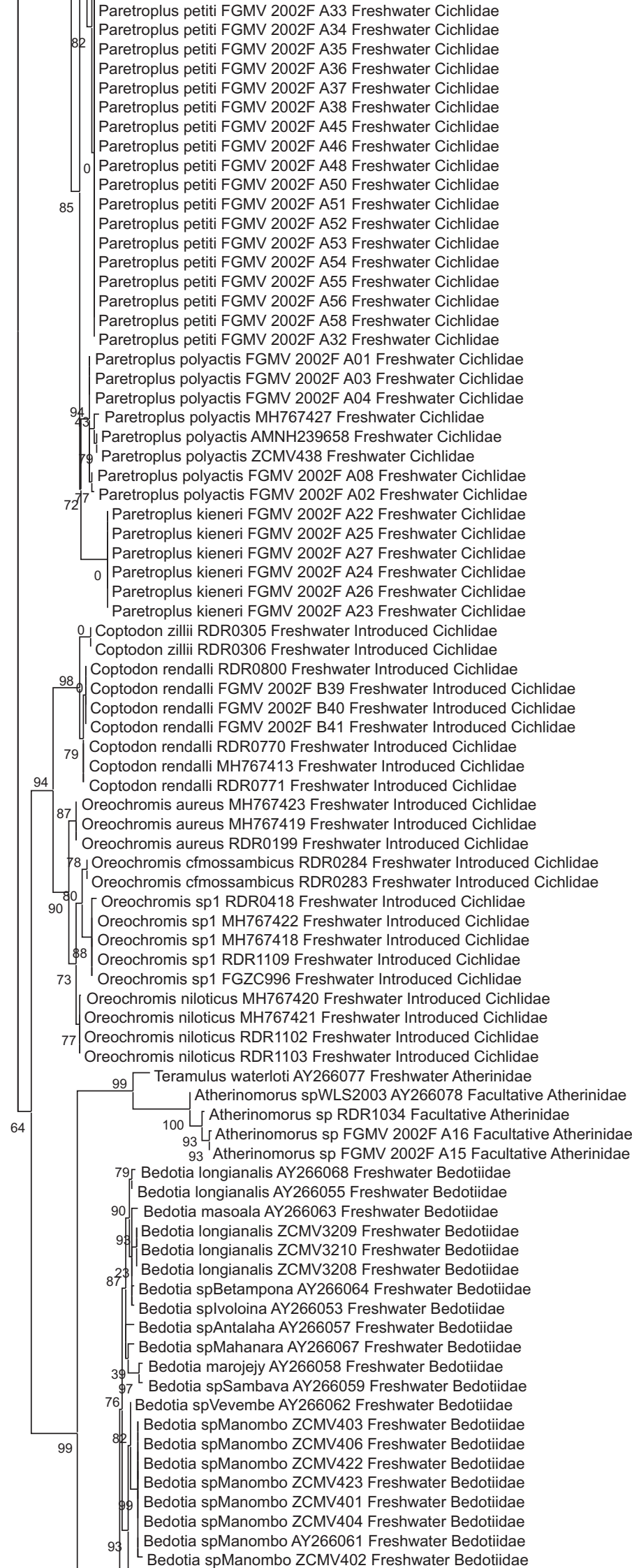

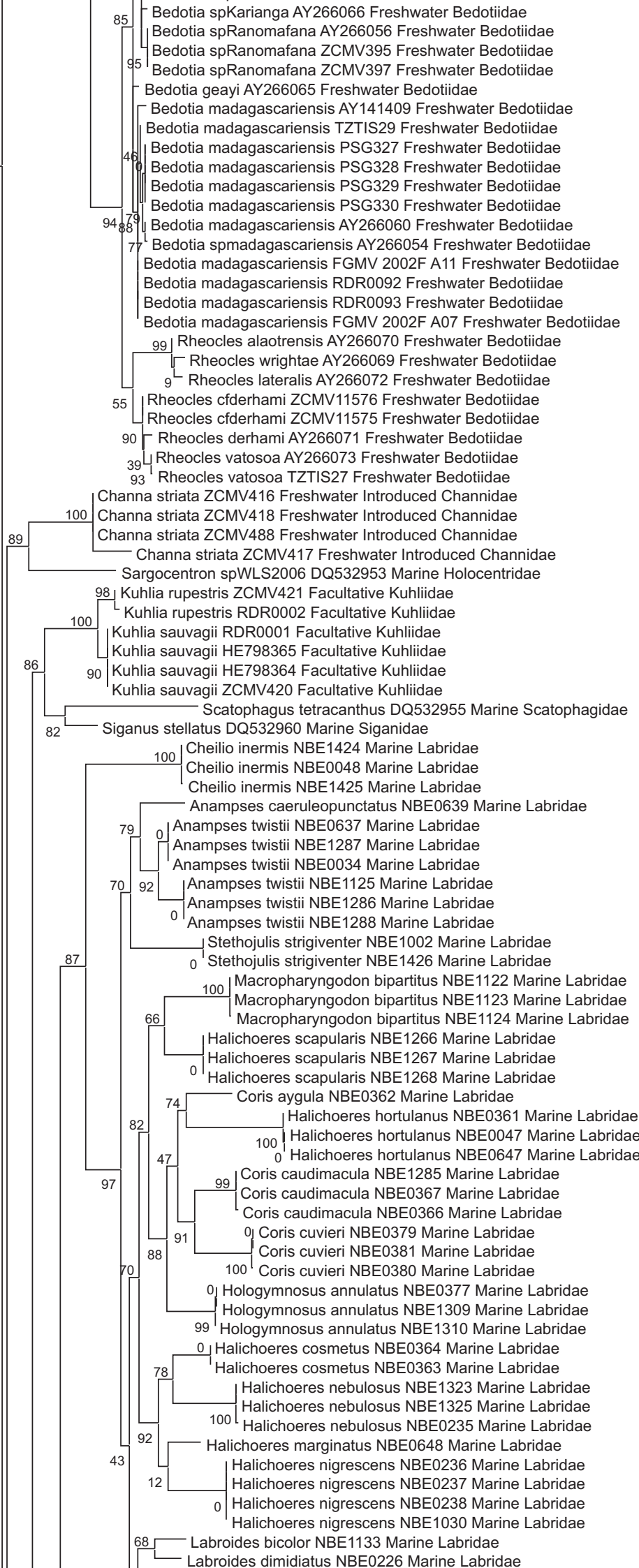

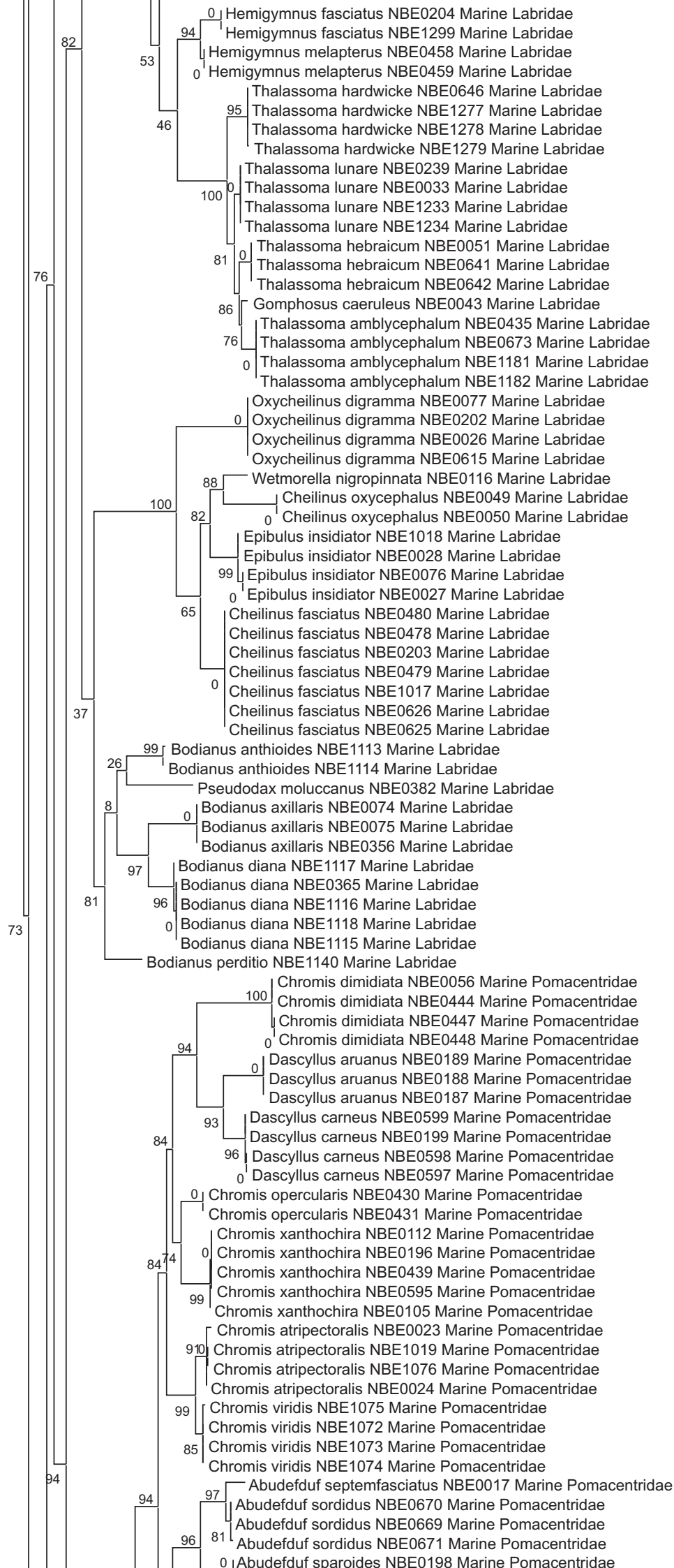

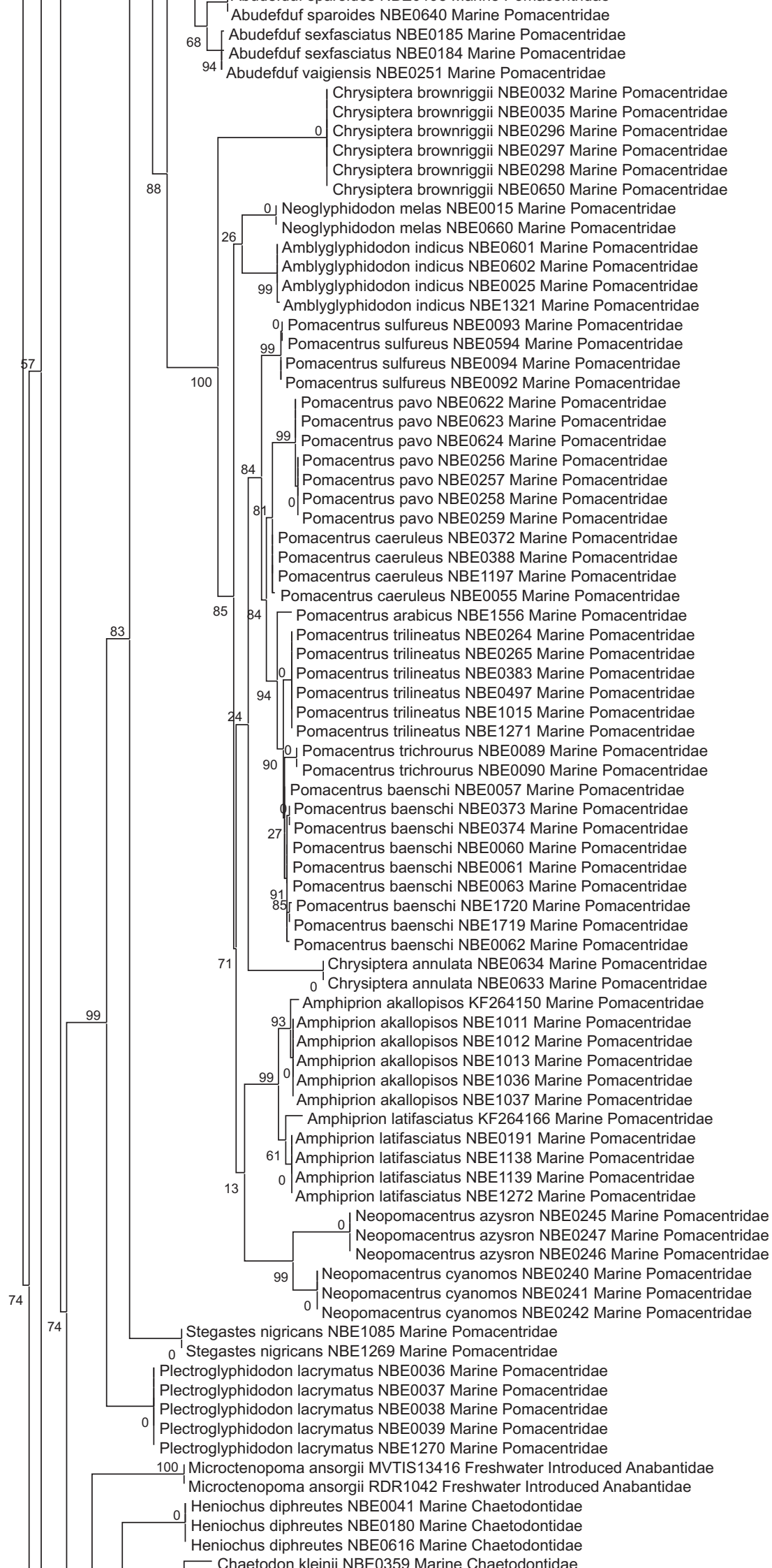

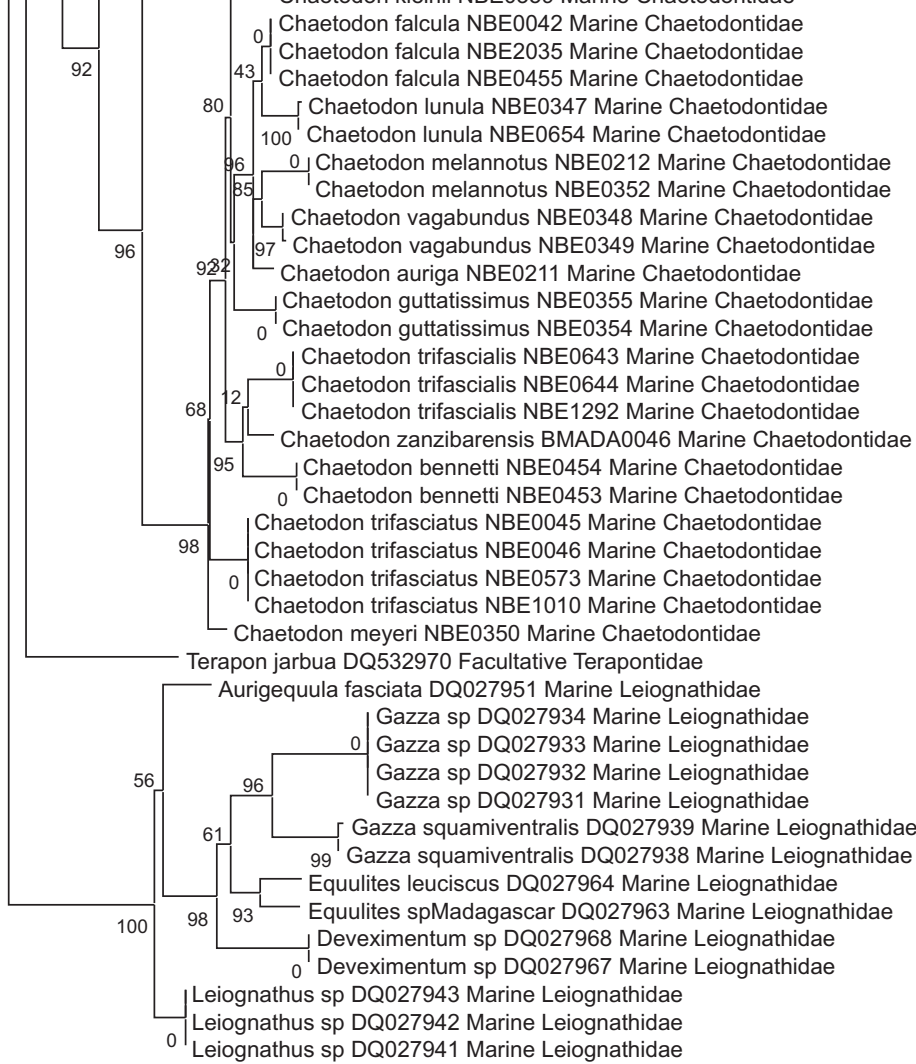

0.05

Supplement: S2 Fig — Approximate Maximum Likelihood tree of Madagascar fishes, calculated with FastTree from 605 partial sequences of the mitochondrial 16S gene, with full labels of all terminals. (PDF) [file pone.0271400.s002.pdf]

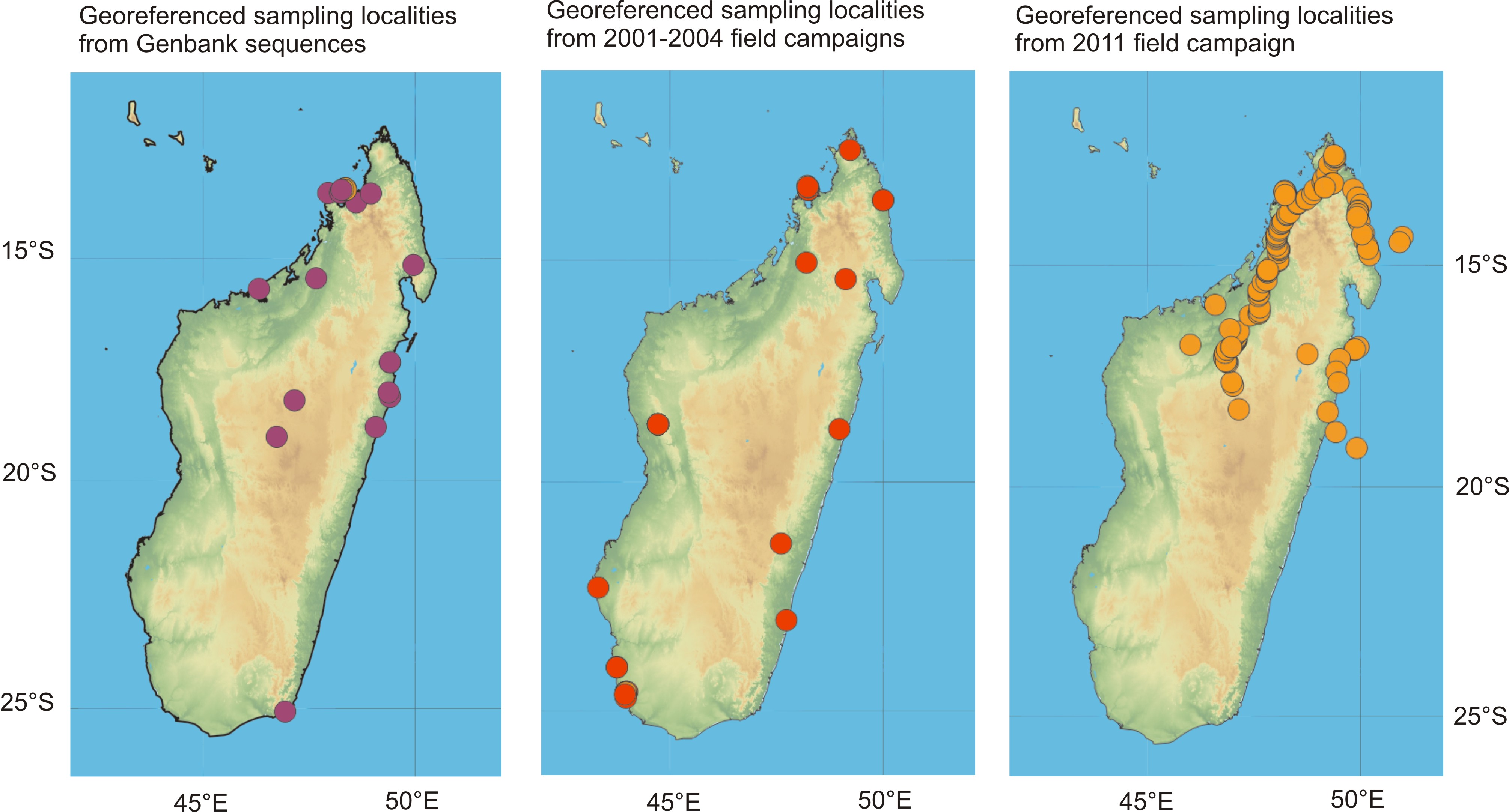

Supplement: S3 Fig — Maps show sampling locations of georeferenced samples in our DNA barcode library, separately from left to right for (i) sequences taken from Genbank, (ii) sequences derived from 2000–2004 field campaigns, and (iii) sequences derived from the 2011 field campaign. Map colors represent elevations; drawn with the open-source Python library matplotlib/basemap (https://github.com/matplotlib/basemap). (JPG) [file pone.0271400.s003.jpg]
